# Kinetically Arrested Twin-Domain State in Formamidinium Lead Iodide

**DOI:** 10.1021/jacs.6c09938

**Published:** 2026-08-12

**Authors:** Xia Liang, Milos Dubajic, Zezhu Zeng, Yang Lu, Johan Klarbring, Samuel D. Stranks, Aron Walsh

**Affiliations:** † Department of Materials, 4615 Imperial College London, South Kensington Campus, London SW7 2AZ, U.K.; ‡ School of Energy and Chemical Engineering, Ulsan National Institute of Science and Technology (UNIST), Ulsan 44919, Korea; § Department of Chemical Engineering and Biotechnology, 2152 University of Cambridge, Philippa Fawcett Drive, Cambridge CB3 0AS, U.K.; ∥ Physical and Theoretical Chemistry Laboratory, Department of Chemistry, 6396 University of Oxford, Oxford OX1 3QZ, U.K.; ⊥ Department of Physics, Chemistry and Biology (IFM), Linköping University, Linköping SE-581 83, Sweden

## Abstract

Hybrid lead halide perovskites exhibit a delicate interplay between average crystallographic symmetry, local structural disorder and A-site orientational dynamics, giving rise to unusual vibrational and electronic behavior. Here, we combine large-scale molecular dynamics with a density-functional-theory-accurate machine learning force field to resolve the structural dynamics of perovskites across mesoscopic length scales. In formamidinium lead iodide (FAPbI_3_), we identify a high-temperature α phase with dynamic local order and correlated tilt nanodomains, an ordered γ phase with long-range *a* ^+^ *a* ^+^ *a* ^+^ tilt coherence, and, below ∼100 K, a history-dependent γ′ state consisting of locally γ-like nanoscale regions separated by sharp twin-like boundaries. This low-temperature disordered state is not a distinct bulk polymorph, but a kinetically arrested metastable twin-domain network selected by the interplay between shallow tilt energetics and slowing FA reorientation. This picture is supported by our low-temperature X-ray diffuse scattering measurements and accounts for the broadened low-energy vibrational response found in the simulations. Furthermore, this unique structural landscape imprints a spatially varying electronic disorder with implications for macroscopic optoelectronic properties, reflected in substantial band-edge broadening retained at low temperature. Our results reconcile the debated low-temperature behavior of FAPbI_3_ in terms of competition between ordered and arrested structural states, and more broadly identify molecular reorientation as a kinetic selector of metastable framework topology in soft molecular crystals, placing thermal history on equal footing with composition as a determinant of structural and optoelectronic properties.

## Graphical abstract

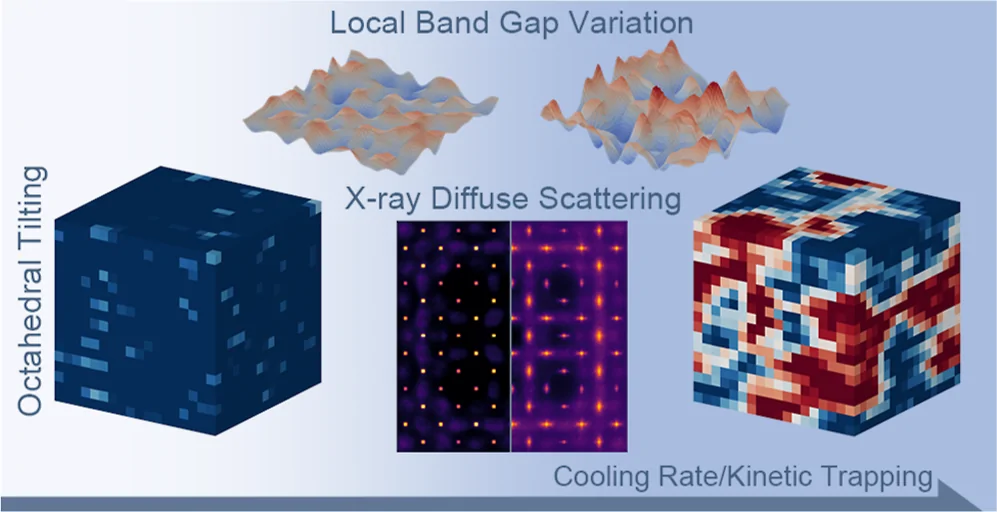

## 1 Introduction

Lead halide perovskites combine soft structure, strong anharmonicity and exceptional optoelectronic performance, making local structural disorder central to both their fundamental physics and functional behavior.^1,2^ In these materials, octahedral tilting, molecular motion and dynamic symmetry breaking are strongly coupled to carrier transport, band-edge broadening and phase stability, so understanding how local order forms, evolves and freezes is essential for connecting structure to function.^3,4^ Recent work has further shown that this disorder is often highly structured rather than random, with dynamic nanodomains providing a concrete real-space manifestation of local order in hybrid perovskites and revealing distinct local textures for different A-site cations.^5–7^ Machine learning force fields (MLFFs) now provide a practical route to near-first-principles molecular dynamics at length and time scales inaccessible to direct ab initio simulation.^8–10^ In perovskites, MLFF-based simulations have increasingly enabled atomistic studies of finite-temperature structural dynamics, local symmetry breaking and phase transitions.^11–13^

At the same time, the low-temperature structure of black-phase FAPbI_3_ remains unresolved. Despite its near-ideal band gap and technological relevance, its structural behavior is unusually delicate: the black perovskite phase is metastable, and its phase sequence is sensitive to thermal history and kinetic trapping.^14–16^ Prior studies have proposed multiple low-temperature structural descriptions, including tetragonal and orthorhombic assignments,^14,17^ while recent machine-learned-potential simulations have identified *a* ^–^ *b* ^–^ *b* ^–^ as the thermodynamic ground state but find that cooling instead leads to a metastable *a* ^–^ *a* ^–^ *c* ^+^ phase.^18^ No consensus has therefore emerged on whether the low-temperature black phase is best described as a unique ordered polymorph or as a history-dependent state shaped by local disorder.^14,15^ This low-temperature behavior is also relevant to extreme environment photovoltaics, where perovskite devices are being explored for space deployment and can experience repeated thermal cycling.^19^

This question is particularly acute in hybrid perovskites because the final structural state need not be determined by local octahedral tilting energetics alone. In FAPbI_3_, the inorganic tilt network is strongly coupled to the orientational degrees of freedom of the FA cation, so the locally preferred tilt motif does not necessarily determine the long-range ordered state that is ultimately reached on cooling. These coupled effects must therefore be investigated explicitly. Small-cell calculations and average structural probes may therefore favor apparently well-defined ordered phases while missing metastable mesoscale textures that emerge only at larger length scales and under specific thermal histories.^18,20,21^

Here, we combine large-scale molecular dynamics with X-ray diffuse scattering and vibrational analysis to resolve the low-temperature structural behavior of black-phase FAPbI_3_ across mesoscopic length scales. We show that FAPbI_3_ passes through a high-temperature α phase with dynamic local order, an ordered γ phase with long-range *a* ^+^ *a* ^+^ *a* ^+^ tilt coherence, and, below ∼100 K, a lower-temperature γ′ regime that remains locally γ-like but develops additional mesoscale structural heterogeneity. This picture is consistent with our measured X-ray diffuse scattering and the calculated low-energy vibrational signatures, and further reveals a spatially heterogeneous electronic landscape associated with the arrested low-temperature state. More broadly, it identifies a mechanism by which the temperature-dependent slowing of molecular reorientation can kinetically select the mesoscale topology adopted by a coupled inorganic framework.

## 2 Results

### 2.1 Large-Cell Dynamics Reveal a Heterogeneous Low-Temperature State in FAPbI_3_

Using large-scale MD simulations analyzed with PDynA,^22^ we resolve three distinct regimes in the structural dynamics of FAPbI_3_. The MLFF was validated against DFT across the relevant energetic, mechanical and finite-temperature structural observables (Figures S1–S4), supporting its use for large-scale dynamical simulations. In the high-temperature α phase (*a* ^0^ *a* ^0^ *a* ^0^; *Pm*3̅m), the inorganic framework exhibits dynamic local order, with correlated tilt nanodomains forming within an average cubic structure. On cooling into the γ phase (*a* ^+^ *a* ^+^ *a* ^+^), which forms below ∼200 K, system-spanning in-phase tilt coherence develops along all three crystallographic directions, consistent with the in-phase cubic Im3̅ framework, which we also verified experimentally by single crystal diffraction (Figure S21). Here, system-spanning coherence refers to fitted correlation lengths that reach or exceed the linear size of the simulation cell, so the correlation lengths fitted in the finite simulation cell need not diverge even for an almost fully ordered γ configuration.

Below approximately 100 K, the low-temperature structures obtained no longer show further growth of this global coherence. This low-temperature crossover is evident in the temperature dependence of the tilt correlation length and the underlying local tilt populations. The tilting correlation length (Figure 1a) increases on cooling through the α-to-γ transition, reaches a maximum near 100 K, and then decreases again at lower temperatures. The local tilt distributions (Figure 1b) show that the low-temperature state remains dominated by the in-phase motif characteristic of the γ phase, demonstrating that most octahedra still belong to locally γ-like environments. At the same time, a smaller but finite population of out-of-phase-correlated neighbors emerges, indicating that the low-temperature state is not uniformly ordered but contains competing local correlations within an otherwise predominantly *a* ^+^ *a* ^+^ *a* ^+^ framework.

**Figure 1:**
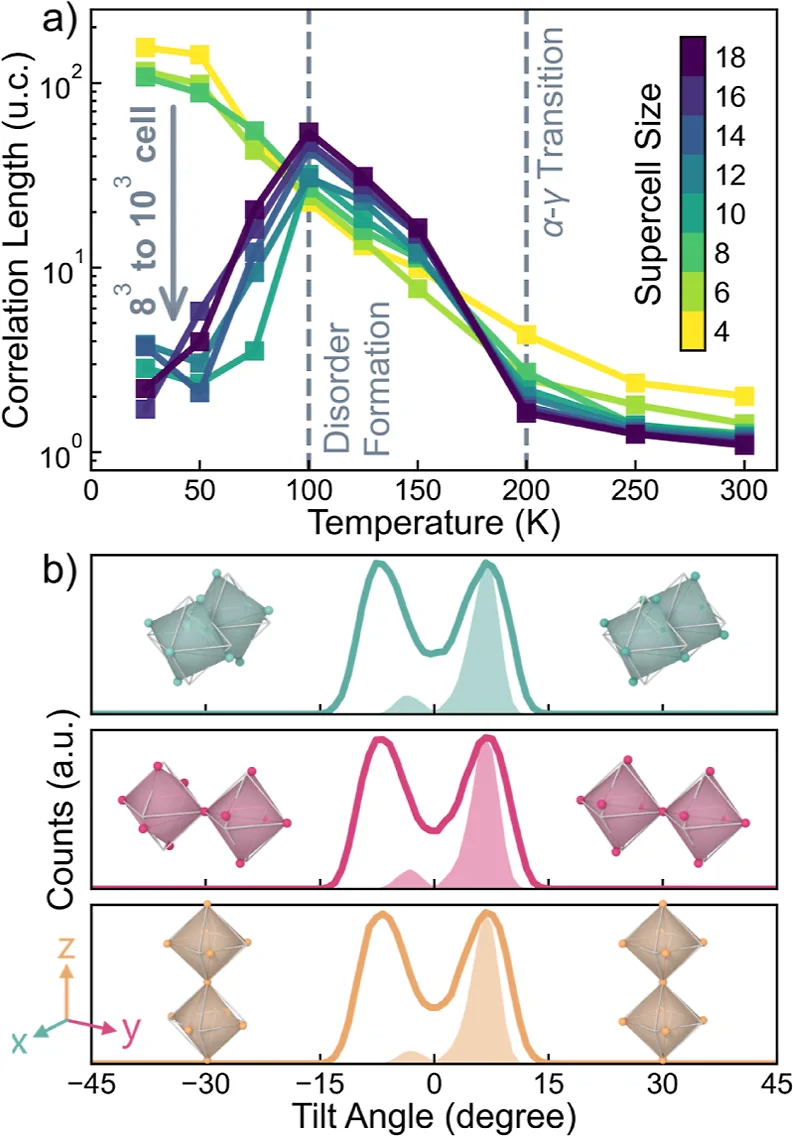
Large-cell simulations reveal a heterogeneous low-temperature state in FAPbI_3_. (a) Temperature dependence of the maximum octahedral tilt correlation length, showing a pronounced maximum near 100 K and strong supercell size dependence in the lower-temperature γ′ regime. (b) Direction-resolved distributions of apparent tilt angles and nearest-neighbor tilting correlations in a 50 K disordered γ′ structure. Solid lines show tilt angle distributions; shaded areas indicate the first–neighbor correlation product, where positive and negative values correspond to in-phase and out-of-phase correlations, respectively.

In the large-cell simulations, the low-temperature disordered structures formed in the 25–75 K range have inferred correlation lengths corresponding to characteristic domain sizes of roughly 2–20 unit cells (∼1.2–12 nm), indicating mesoscale domains whose characteristic sizes vary with temperature and configuration rather than a uniform bulk-ordered crystal. A pronounced finite-size effect further supports this interpretation: the γ′ state emerges only when the simulation cell exceeds approximately 10 × 10 × 10 pseudocubic units, whereas smaller cells remain trapped in a fully correlated γ-like configuration. These results therefore support a heterogeneous low-temperature state rather than a unique ordered low-temperature phase,^23^ a conclusion further corroborated below by the agreement between the simulated and measured single crystal X-ray diffuse scattering patterns.

### 2.2 Shallow Tilt Energetics and FA Dynamics Frustrate Long-Range Ordering

To rationalize the origin of the low-temperature heterogeneous state, we evaluated the tilt-dependent potential energy surface predicted by the MLFF in a 2 × 2 × 2 supercell with randomized molecular orientations, such that the resulting landscape probes the local energetic response to octahedral tilting without imposing long-range molecular order (noting that these are local potential energies rather than finite-temperature free energies; see Section S5 in the Supporting Information). Here we restrict the discussion to the subspace spanned by octahedral tilt amplitudes and idealized tilt patterns.^24^

The potential energy surfaces in Figure 2 reveal a strong A-site dependence of the local tilt energetics. In CsPbI_3_, the landscape is comparatively steep and well-defined, closer to a conventional lattice-dynamical picture. By contrast, the molecular perovskites, especially FAPbI_3_, exhibit a flatter and more frustrated manifold in which symmetry-distinct tilt modes are only weakly separated. Instead, the landscape is organized mainly by the number of active tilt axes: *a* ^0^ *a* ^0^ *c* ^–^ and *a* ^0^ *a* ^0^ *c* ^+^ remain close in energy, as do *a* ^+^ *a* ^+^ *a* ^+^ and *a* ^–^ *a* ^–^ *c* ^+^. This shows that the local energetic cost in the molecular systems is governed primarily by the magnitude and dimensionality of octahedral rotation, while the in-phase versus out-of-phase registry is only weakly differentiated locally and must therefore be selected cooperatively through longer-range distortions and coupling to the A-site degrees of freedom. Bond-length relaxation also modifies these potential energy surfaces (Figure S16); here we focus on the tilt-angle dependence to isolate how the local energetic hierarchy varies with A-site chemistry and tilt topology.

**Figure 2:**
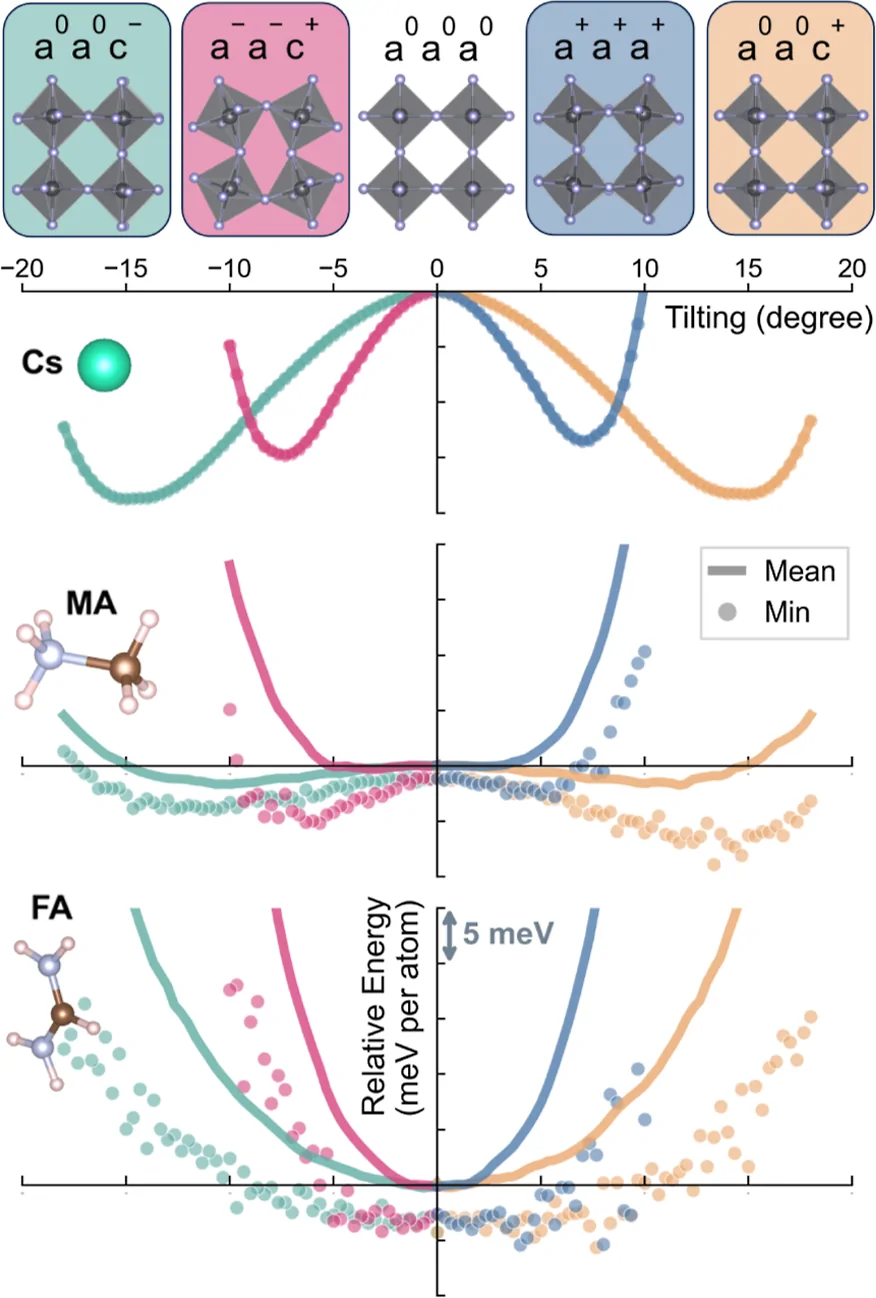
Tilt-dependent potential energy surfaces of iodide perovskites. Relative energies of CsPbI_3_, MAPbI_3_, and FAPbI_3_ are shown as a function of tilt angle for the idealized modes *a* ^0^ *a* ^0^ *c* ^–^, *a* ^–^ *a* ^–^ *c* ^+^, *a* ^+^ *a* ^+^ *a* ^+^, and *a* ^0^ *a* ^0^ *c* ^+^. The zero-tilt limit corresponds to the cubic *a* ^0^ *a* ^0^ *a* ^0^ structure. Solid lines show the mean over 50 independent runs, and points indicate the minimum-energy structures. The molecular perovskites display a shallow, frustrated tilt landscape in which the number of active tilt axes matters more than the in-phase versus out-of-phase registry alone.

This weak local energetic discrimination points to a kinetic contribution to the formation of the γ′ state. Rather than reflecting a fixed separation between tilt and molecular time scales, the relevant feature is the strong temperature-dependent slowing of the coupled molecular–framework dynamics. In the γ-formation window, approximately 100–200 K, the characteristic times for tilt decorrelation and FA reorientation are of the same broad order and remain sufficiently short for cooperative molecular–framework rearrangements to be sampled during the MD trajectories (Figures S11c and S14). Below this window, all of these characteristic times increase sharply relative to their values near 150 K, reaching a slowdown of approximately two to three orders of magnitude over the lowest temperatures considered (Figure S15). The cooperative rearrangements required to reach an ordered γ state containing a single coherent domain therefore become increasingly inaccessible on the MD time scale. Together with the lower static energy of the ordered γ phase relative to γ′ (Figure S20), this collective slowing indicates that the low-temperature heterogeneous state arises from kinetic arrest of the coupled ordering process rather than from a separate lower-energy static structure.^15^

To resolve the molecular ordering component of this kinetic process, we classify nearest-neighbor FA pairs by their relative molecular vector orientations. In this analysis, FA1 and FA2 denote the two orthogonal in-plane molecular vectors used to coarse-grain the orientation of each FA cation; their geometrical definition is shown in Figure 3a. This scheme is justified by independent tests in which fully randomized FA orientations relax back into a ⟨100⟩ orientational manifold after FA-only relaxation, with both FA1 and FA2 vectors concentrated near the six Cartesian ⟨100⟩ directions (Figure S17). The resulting FA-pair statistics reveal a clear change in molecular ordering across the three regimes (Figure 3b). In the α phase, the orientational correlation landscape remains close to a random benchmark, defined as the orientational correlation statistics expected in the absence of intermolecular orientational correlations, indicating that molecular orientations are only weakly correlated despite the presence of dynamic local tilt correlations. On entering the γ phase, however, a symmetry-selected anisotropic molecular ordering pattern emerges, consistent with long-range *a* ^+^ *a* ^+^ *a* ^+^ tilt coherence. In the γ′ state, by contrast, this anisotropic molecular order does not develop globally; instead, the FA sublattice freezes into disconnected orientational variants associated with locally γ-like framework regions.

**Figure 3:**
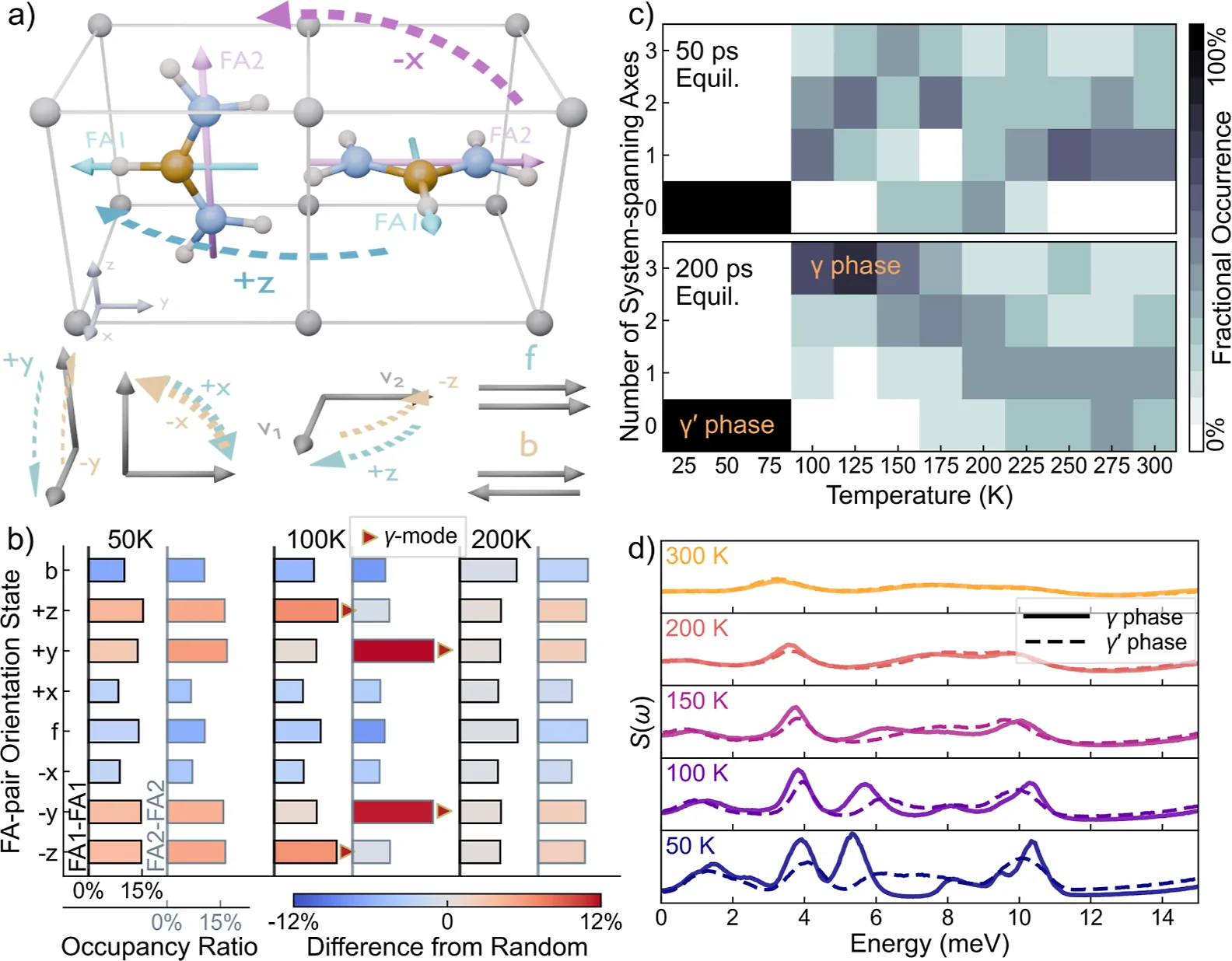
FA orientational correlations select the low-temperature structural outcome in FAPbI_3_. (a) Definition of the FA1 and FA2 molecular vectors and the discrete pair-correlation states used to classify nearest-neighbor molecular configurations. (b) Relative populations of FA1–FA1 and FA2–FA2 correlation states at selected temperatures along the *x* direction; colors indicate deviation from the random benchmark. The horizontal axis reports the occupancy ratio of each nearest-neighbor FA-pair orientation state. The FA1–FA1 and FA2–FA2 statistics are displayed on the same occupancy scale for direct comparison. (c) Fractional occurrence of low-temperature structural outcomes under controlled equilibration protocols, classified by the number of crystallographic directions with system-spanning tilt correlation length, defined as a fitted correlation length that reaches or exceeds the simulation-cell length. (d) Neutron *q*-integrated vibrational spectra *S*(ω) for structures initialized from ordered γ and disordered γ′ states. The ordered γ phase exhibits sharper low-energy features, whereas the γ′ spectra are broader and more reminiscent of the higher-temperature response, consistent with weaker long-range molecular order in the twin-domain state.

To test whether the final low-temperature structure is controlled by the prepared molecular state, we performed controlled MD protocols in which the FA orientations were equilibrated for different hold times before applying a temperature ramp to 50 K, where the structural dynamics are effectively frozen. The resulting phase map (Figure 3c) shows that the dependence on equilibration history is concentrated primarily in the approximate range 100–175 K. Below approximately 100 K, no substantial molecular reorganization occurs and both hold times consistently lead to the heterogeneous γ′ state. Within the 100–175 K window, increasing the hold time from 50 to 200 ps shifts the outcome toward the fully ordered γ topology, increasing the occurrence of configurations with three system-spanning axes. At higher equilibration temperatures, orientational correlations weaken and a broader distribution of low-temperature topologies becomes accessible. These orientation-control simulations therefore provide direct evidence for this kinetic interpretation, showing that molecular reorganization is a key kinetic constraint on the formation of the ordered γ state.

This crossover is captured in our simulations by the sharp rise in molecular reorientation times and by the broader calculated *q*-integrated neutron spectra of the γ′ state relative to the ordered γ phase (Figure 3d; integration range given in Methods). The corresponding Arrhenius analysis indicates an effective reorientational barrier for FA motion (Figure S18), such that at lower temperature the FA subsystem can no longer reorganize efficiently, consistent with previous reports of hindered FA dynamics,^14^ and the structural outcome becomes effectively fixed, whereas at higher temperature barrier crossing becomes possible and a wider range of low-temperature topologies can be accessed. The different outcome distributions obtained after 50 and 200 ps equilibration further show that, although both hold times are short on laboratory time scales, they are sufficient on the MD time scale to resolve these differences in kinetic accessibility. The low-temperature structural outcome is therefore selected by the interplay between a shallow local tilt manifold, the rapid slowing of coupled molecular–framework dynamics on cooling, and the kinetic accessibility of molecular reorganization.

### 2.3 Reciprocal-Space and Electronic Fingerprints of the Arrested Twin-Domain State

To connect the real-space analysis with experimentally accessible observables, we computed the dynamical structure factor *S*(**q**, ω) following our previous framework.^21^ The calculated diffuse scattering cleanly distinguishes the three structural regimes (Figure 4a): dynamic *M*-point intensity in the high-temperature α phase, strongly suppressed diffuse scattering in the ordered γ phase, and broad, configuration-dependent features near the Bragg peaks and *M* points in the low-temperature γ′ state. These low-temperature signatures are incompatible with both the dynamic nanodomain disorder of the α phase and the long-range coherence of the ordered γ phase, and instead point to frozen spatial heterogeneity within an otherwise locally γ-like framework.

**Figure 4:**
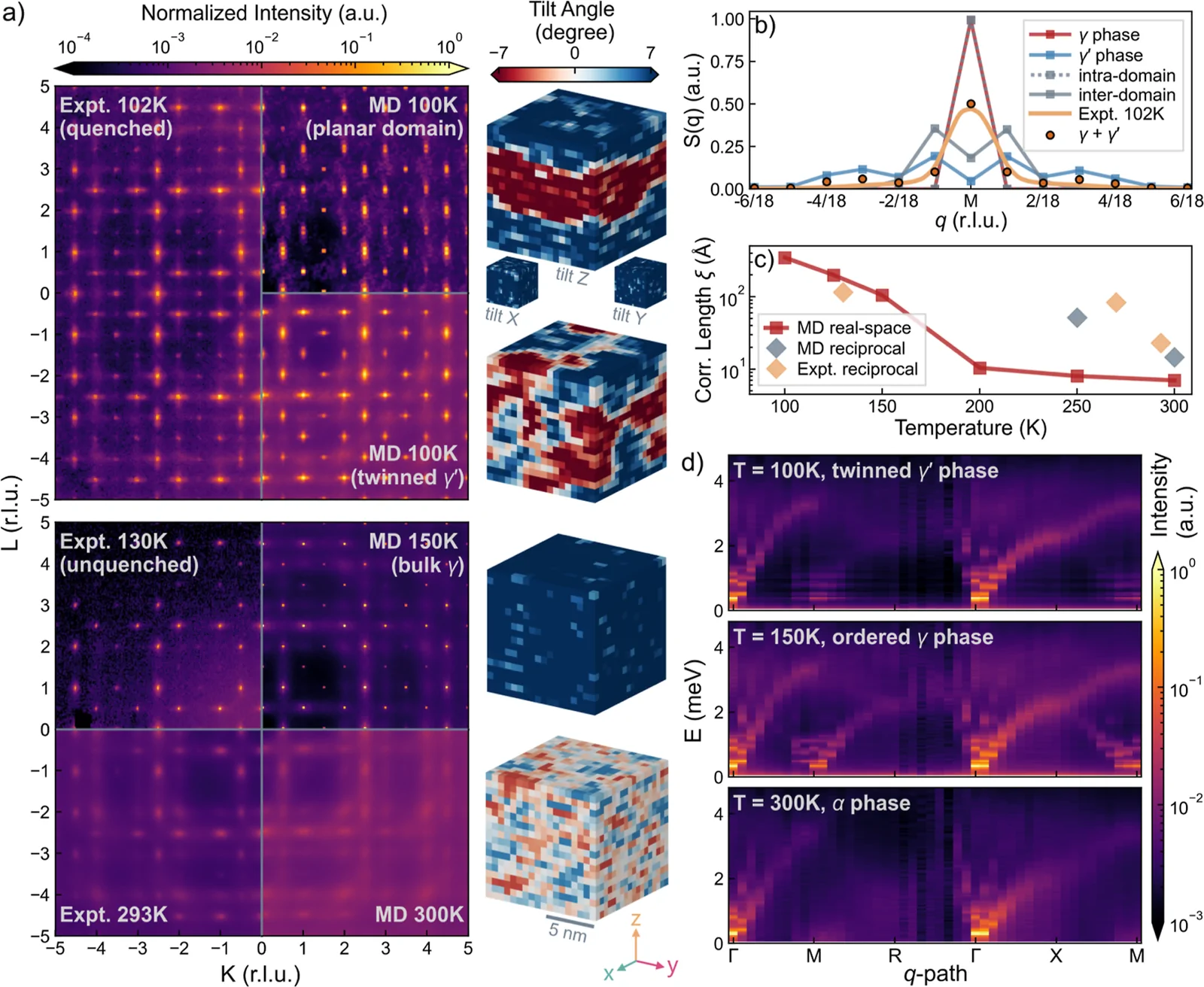
Reciprocal-space signatures of FAPbI_3_ under different regimes. (a) X-ray diffuse scattering on *L* = 1.5 r.l.u. half-integer reciprocal-space planes for experiment and simulation, together with one-to-one corresponding MD real-space maps of the local octahedral tilts. For the planar domain structure, additional maps of the *x*- and *y*-tilt components are shown to highlight its anisotropic domain topology. The top panels compare the quenched low-temperature experimental pattern with two simulated low-temperature limits, namely a planar twin-domain structure and the twinned γ′ state. The bottom panels compare the experiment with the bulk ordered γ phase at intermediate temperature and the dynamically disordered α phase at high temperature (intensity rescaled for display purposes). The simulated γ′ diffuse map is averaged over four independent trajectories and three symmetry-equivalent planes (12 patterns total; Figure S25). (b) Line profiles around symmetry-equivalent *M* points for the ordered γ phase, the twinned γ′ state, the planar domain structure, experiment, and a 50:50 γ + γ′ combination. (c) Correlation lengths extracted from real-space tilting correlations and reciprocal-space peak widths for simulation and experiment. (d) Calculated energy-resolved dynamical structure factor *S*(**q**, ω) along a high-symmetry path from MD trajectories for the twinned γ′, ordered γ, and α phases.

This assignment is supported directly by the real-space tilt maps and their experimental counterparts. The simulated γ′ structures break up into locally γ-like regions separated by sharp orientational discontinuities, and the corresponding diffuse scattering pattern reproduces the low-temperature features observed in the quenched sample, whereas the ordered γ and dynamically disordered α structures each match their respective experimental counterparts at intermediate and high temperature. This interpretation is also consistent with the thermal protocols: rapid quenching bypasses sustained equilibration in the intermediate γ regime, leaving insufficient time for the FA subsystem to develop the anisotropic alignment required for long-range γ-phase coherence.

To isolate the role of the interfaces themselves, we constructed an artificial planar domain structure containing a single twin boundary. This control structure preserves coherent in-domain tilts while introducing a single noncoherent twin boundary, across which the in-phase tilt coherence changes abruptly, allowing the reciprocal-space signature of the interface to be separated from that of the local *a* ^+^ *a* ^+^ *a* ^+^ motif. In this case, the reciprocal-space anisotropy becomes explicit: the *HK* half-integer plane remains essentially identical to the ordered γ phase, whereas the *HL* and *KL* planes develop pronounced satellite intensity around the *M* points, and the near-*M* profile acquires a characteristic double-peak line shape (Figure 4b). These results show that the additional diffuse weight in the γ′ state originates from twin boundaries rather than from a different local tilt motif. The low-temperature γ′ regime is therefore best understood as a kinetically arrested twin-domain state, consisting of locally γ-like regions separated by sharp interfaces. Neither idealized simulated limit, namely the fully ordered γ structure or the fully twinned γ′ structure, reproduces all experimental peak widths and relative intensities quantitatively. This is expected because the measured profile is affected by finite reciprocal-space resolution, experimental broadening, and the coexistence of differently ordered regions within the illuminated volume. A simple 50:50 combination of the two simulated signals nevertheless closely tracks the measured intensity, consistent with the experimental low-temperature sample containing both nearly ordered γ-like regions and twinned metastable domains. This comparison should be viewed as a simplified physical illustration of mixed structural contributions rather than as a unique decomposition or exact phase fraction of the experimental signal.

Correlation lengths extracted from real-space tilting correlations and from reciprocal-space peak widths track the same structural crossover but represent distinct, observable-dependent length scales (Figure 4c). The real-space quantity measures the decay of local tilt correlations in the MD configurations, whereas the reciprocal-space quantity is inferred from diffuse feature widths and additionally depends on the fitted line shape and finite reciprocal-space resolution; numerical equality between the two is therefore neither expected nor required. The most direct comparison with experiment is therefore the reciprocal-space comparison, where simulated and measured line profiles are analyzed using the same peak width procedure. Within this common reciprocal-space framework, the simulated and experimental correlation lengths exhibit good quantitative agreement across the phases for which well-defined peak widths can be extracted. In this representation, the dynamically disordered high-temperature phase has short correlations, whereas the ordered γ phase gives much sharper, often resolution-limited features (Figure S24). The lower-temperature γ′ regime is not assigned a single reciprocal-space correlation length here because its near-*M* line shape is non-Lorentzian and configuration dependent; its finite real-space domain sizes are instead shown in Figure 1a. In addition, the simulated transition temperature is depressed relative to experiment, a known limitation of current MLFF-based phase-transition modeling.^25,26^

The calculated energy-resolved dynamical structure factor highlights the same distinction (Figure 4d). The clearest contrast appears at the zone-boundary *M* point, which is dominated by octahedral tilt fluctuations. In the ordered γ phase, the low-temperature response remains comparatively sharp, consistent with coherent long-range *a* ^+^ *a* ^+^ *a* ^+^ order. By contrast, the twinned γ′ state exhibits a substantially broader low-energy response at the same wave vector. Because this broadening persists even at low temperature, it cannot be attributed to thermal disorder alone, but instead reflects the static structural heterogeneity introduced by the arrested twin-domain topology.^27,28^ This dynamical contrast is also consistent with the molecular behavior: the ordered γ phase, with its well-developed anisotropic FA correlation pattern, yields sharper low-energy spectral features, whereas the partially ordered γ′ state produces broader spectra (Figure 3d) that more closely resemble the anomalous low-temperature neutron response reported experimentally.^29^ In this sense, both the calculated dynamical structure factor and the neutron data support the same picture of the arrested low-temperature state with incomplete long-range molecular and tilt coherence.

The same arrested twin-domain texture is also electronically consequential. Structural heterogeneity in hybrid halide perovskites is known to modulate the electronic structure through coupling between lattice distortions, molecular motion and band-edge states.^30–32^ To probe this relationship, we partition each large MD supercell into 2 × 2 × 2 subcells and perform DFT calculations for each local environment, thereby constructing a spatially resolved electronic landscape tied to the mesoscale structural texture. Across these subcells, the local band gap varies substantially and correlates most strongly with the octahedron-averaged tilt norm (Figure S30), identifying local octahedral tilting as a robust descriptor of relative band gap variation. Because periodically repeated subcells introduce a defined spatial coarse-graining scale, we also repeated the analysis using 4 × 4 × 2 subcells extracted from the same parent configuration. Both subdivisions retain a positive tilt–gap correlation, while the larger subcells produce narrower distributions because they average over a greater local volume (Figure S29). The analysis therefore focuses on systematic electronic differences among local structural environments, while recognizing that the absolute distribution widths depend on the selected coarse-graining scale.

Representative unfolded band structures for strongly and weakly tilted subcells are shown in Figure 5a,b, with the corresponding SOC-inclusive band-edge states overlaid. SOC primarily lowers the conduction band edge, reducing the gap by approximately 1 eV, while the direct-gap character at *R* and the principal near-edge dispersion features are retained, consistent with previous calculations for FAPbI_3_.^33^ Importantly, the more weakly tilted subcell exhibits a smaller band gap than the strongly tilted subcell both with and without SOC, and a matched statistical comparison confirms that SOC preserves the relative ordering of low- and high-gap local environments (Figure S27). Across the full data set, this relation becomes especially clear when the local band gap is plotted against the octahedron-averaged tilt norm (Figure 5c), establishing a direct connection between the local structural motif and the local electronic response.

**Figure 5:**
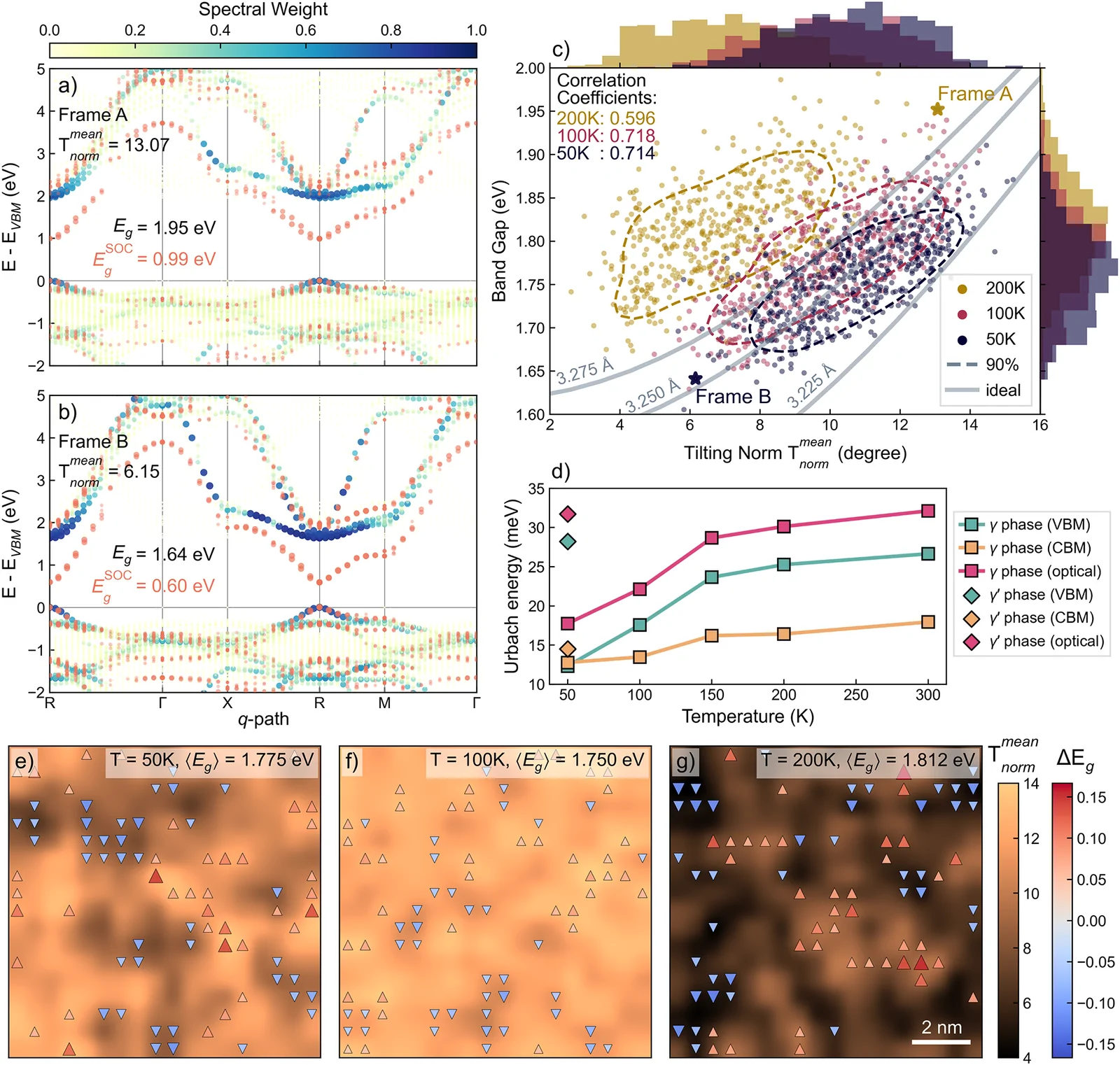
Electronic fingerprints of mesoscale tilt heterogeneity in FAPbI_3_. (a,b) Unfolded band structures for representative subcells with large and small local tilt magnitudes. The non-SOC states are colored by spectral weight, while the corresponding SOC-inclusive states are overlaid in orange with marker sizes proportional to their spectral weights. The SOC and non-SOC energies are independently referenced to their respective valence-band maxima. (c) Local band gap as a function of the subcell tilt metric for snapshots sampled at 50, 100, and 200 K; contours enclose 90% of the data and stars denote the two representative subcells in (a,b). The distributions of local band gaps and tilt metrics are shown at the right and top edges of the map, respectively. Gray lines indicate idealized reference relations with varying Pb–I bond lengths. (d) Urbach energies extracted from the distributions of local valence and conduction band-edge energies, together with the resulting effective optical Urbach energy. While the overall increase with temperature reflects growing thermal disorder, the low-temperature γ′ state retains substantial band-tail broadening due to static twin-domain heterogeneity. (e–g) Real-space maps of local tilt magnitude and local band gap deviation, showing that electronic extrema track the morphology of the tilt-domain network.

This relation is also visible directly in real space, as shown in Figure 5e–g. Planar slices through the supercell show that local band gap extrema track the morphology of the tilt-domain network: regions with different local electronic gaps are not randomly distributed, but are spatially organized by the twin-domain texture. In practice, the electronic landscape follows the mesoscale structural landscape, both in the dynamically fluctuating high-temperature regime and, more strikingly, in the low-temperature γ′ state where the domain pattern becomes quasi-static on the simulation time scale.

The temperature dependence of the extracted Urbach energies is shown in Figure 5d. As expected, the overall band-tail broadening increases with temperature as thermal disorder grows. The calculated values lie on the same approximate energy scale as the room-temperature Urbach energies typically reported for high-quality FAPbI_3_ films and devices, which are in the range of ∼14–22 meV depending on sample quality, processing route, and measurement protocol,^34,35^ although the present estimates are somewhat larger. This comparison is intended to establish the physical scale and qualitative temperature dependence of the calculated broadening rather than to imply direct quantitative reproduction of an equilibrium high-quality thin-film absorption edge. The low-temperature γ′ state is nevertheless anomalous: its Urbach broadening remains comparable to that of the 300 K structure despite the large reduction in thermal motion. This shows that if cooling drives the system into the arrested γ′ topology rather than the ordered γ phase, static twin-domain heterogeneity can replace thermal disorder as the dominant source of low-temperature band-tail broadening. The arrested twin-domain texture therefore explains not only the anomalous low-temperature reciprocal-space signatures of black-phase FAPbI_3_, but also the persistence of substantial electronic disorder deep into the low-temperature regime. The SOC sensitivity analysis (Figure S27) further shows that SOC redistributes the broadening between the valence and conduction edges, while retaining the same order of magnitude and temperature-dependent increase in the band-edge fluctuations.

## 3 Summary and Discussion

We have shown that the low-temperature structure of black-phase FAPbI_3_ is best understood not as a unique long-range-ordered polymorph, but as a kinetically arrested twin-domain state composed of locally ordered *a* ^+^ *a* ^+^ *a* ^+^ regions separated by sharp twin-like interfaces. Large-cell molecular dynamics reveal that this γ′ state emerges only when the system is allowed to develop mesoscale structural heterogeneity, establishing twinning as an intrinsic feature that emerges only at large length scales rather than a small-cell artifact. The origin of this behavior lies in the coupled dynamics of octahedral tilting and FA reorientation. In FAPbI_3_, the local energy landscape for octahedral tilting is shallow and only weakly constrains the final tilt registry, so the long-range structural outcome must be selected cooperatively through the coupled evolution of the inorganic tilt network and the molecular orientational degrees of freedom. This cooperative ordering becomes kinetically constrained on cooling: below the γ-formation window, the framework and molecular correlation times both increase by orders of magnitude relative to their values near 150 K, while controlled hold-time simulations show that additional molecular equilibration before cooling promotes the ordered γ topology. Together with the lower static energy of the ordered γ phase, these observations show that the low-temperature twin-domain topology reflects kinetic arrest of the coupled ordering process rather than a distinct thermodynamic ground state.

This arrested structural state leaves clear reciprocal-space, vibrational, and electronic fingerprints. It reproduces the diffuse scattering features of quenched low-temperature samples, and the artificial planar domain control shows that the characteristic additional diffuse weight arises from the twin boundaries themselves rather than from a different local tilt motif. The same arrested topology also broadens the low-energy dynamical response and generates a spatially heterogeneous electronic landscape, with local band gaps that track the mesoscale tilt texture and band-edge distributions that remain substantially broadened despite the reduction in thermal motion. In this sense, if cooling drives the system into the arrested γ′ topology rather than the ordered γ phase, static structural heterogeneity can replace thermal disorder as a dominant source of low-temperature electronic broadening. This also has direct implications for the interpretation and reproducibility of measurements in FAPbI_3_: differences in reported Urbach energies, photoluminescence line widths, carrier lifetimes, and scattering signatures across nominally similar samples may in part reflect distinct arrested structural textures rather than only differences in material quality or defect density. Thermal history should therefore be considered alongside composition and defect chemistry as a determinant of structure and optoelectronic behavior in hybrid halide perovskites.

More broadly, our results place static twinning alongside dynamic nanodomain formation as a fundamental mode of local order in perovskites. They show that, in hybrid organic–inorganic compounds, molecular A-site dynamics do not merely decorate an inorganic framework, but can act as kinetic selectors that determine how disorder is organized, correlated, and frozen into the structure. This mechanism should be relevant to soft molecular crystals in which molecular reorientation is coupled to a deformable framework, because the observed state can then be determined not only by thermodynamic stability but also by the relative relaxation time scales of the molecular and framework sublattices. In this respect, black-phase FAPbI_3_ joins a broader class of kinetically governed functional materials, including structural glasses and relaxor ferroelectrics. This perspective places the thermal history sensitivity and sample-to-sample variability widely observed in hybrid perovskites within this broader framework of kinetic control.

## 4 Methods

### 4.1 Force Field Training

Although the present study focuses primarily on FAPbI_3_, with smaller comparative analyses of MAPbI_3_ and CsPbI_3_, the MLFF was trained over the broader chemical space $\left({Cs}_{1-x-y}{MA}_{x}{FA}_{y}\right)Pb{\left({Br}_{m}I_{1-m}\right)}_{3}$ . The aim is to construct a single unified MLFF that enables direct comparison across chemically related compounds within one internally consistent potential energy framework, while also providing a reusable model for future studies of mixed-A-site and mixed-halide lead perovskites. For this seven-element chemical space, however, our previous workflow based primarily on direct on-the-fly sampling becomes prohibitively expensive.^22,36,37^ We therefore adopt a two-stage strategy in which a preliminary surrogate MLFF is first constructed from a limited but chemically representative DFT data set, and is then used to generate a much larger set of candidate structures for final data selection and retraining.

As the starting point for this workflow, an on-the-fly DFT training is performed for a single mixed composition, Cs_2_MA_3_FA_3_Pb_8_Br_12_I_12_, in a 2 × 2 × 2 supercell. This composition lies close to the center of the doubly mixed composition space and therefore contains representative local A-site and halide interactions. The A-sites are randomly arranged, and the halides are distributed using the ICET package.^38^ The initial inorganic framework is assigned the *a* ^0^ *a* ^0^ *a* ^0^ tilt pattern, since the equilibrium tilt state of this mixed composition is not known a priori. Four on-the-fly MD simulations are then carried out at 100, 300, 500, and 700 K to sample a broad thermal range with a tractable number of DFT calculations.

All DFT calculations were performed in VASP using the projector-augmented-wave method^39^ and the r^2^SCAN meta-GGA functional.^40^ For the on-the-fly MD stage, we used a plane-wave cutoff of 500 eV, a Γ-centered 2 × 2 × 2 *k*-point mesh, Gaussian electronic smearing with a width of 0.05 eV, and an electronic self-consistency threshold of 10^–5^ eV. The on-the-fly trajectories were propagated in the *NpT* ensemble with a 0.5 fs time step and Langevin dynamics.

A total of 2255 snapshot frames from these four on-the-fly trajectories are combined to train a preliminary MACE model.^10^ The data are split into training and validation sets using a 90:10 ratio, and the model is trained for 2000 epochs. Because this model is used only as a surrogate sampler for a broad and sparsely covered configuration space, we employ a relatively expressive architecture despite its higher evaluation cost. Specifically, the preliminary model uses a 7 Å radial cutoff, 128 feature channels, and message equivariance up to *L* = 1. The resulting validation accuracy is sufficient for structure generation and diversity sampling, but this preliminary model is not used for the final production simulations.

The preliminary model is then used to sample the doubly mixed compositional space using a structured surrogate-MD workflow over representative mixed compositions and initial tilt states. The detailed compositional sampling scheme is summarized in Figure S6 and Table S2. This produces a total of 175 surrogate MD simulations in the *NpT* ensemble, each 300 ps in duration. We extract 200 frames from each simulation by saving one structure per picosecond over the final 200 ps, resulting in 35,000 candidate structures.

To reduce the cost of DFT labeling, the candidate pool was down-selected using the DIRECT method.^41^ From the 35,000 surrogate-sampled frames, 3000 structures were selected as the main mixed-composition DFT-labeled data set, and 1000 additional end point-family structures were included for each of CsPbX_3_, MAPbX_3_, and FAPbX_3_.^42^ These end point families span the corresponding halide subspaces, i.e. Pb­(Br_*m*_I_1–*m*_)_3_ with fixed A-site identity. The final training set therefore comprised 6000 mixed- and endpoint-family structures. This data set construction process is further evaluated in Section S3 of the Supporting Information. For the final single-point labeling calculations, the same general DFT setup was retained, but with a higher plane-wave cutoff of 600 eV and a tighter electronic self-consistency threshold of 10^–7^ eV. The *k*-point sampling was chosen according to the supercell size, using a Γ-centered 2 × 2 × 2 mesh for the 2 × 2 × 2 cells and a Γ-centered 1 × 1 × 2 mesh for the 4 × 4 × 2 cells. These calculations were carried out as static single-point evaluations to obtain energies, forces, and stresses for the final training set.

The final production MACE model is trained on this curated data set using a 90:10 train–test split and a training length of 1000 epochs. The model uses a 5 Å cutoff, 64 feature channels, and message equivariance up to *L* = 0, which substantially reduces the inference cost while retaining sufficient accuracy for large-scale MD.

### 4.2 Molecular Dynamics

Large-scale MD simulations are carried out in LAMMPS^43^ through the MACE-MLIAP interface, combined with the cuEquivariance-enabled workflow for efficient GPU-accelerated production simulations. Unless otherwise noted, we use an 18 × 18 × 18 pseudocubic supercell (69,984 atoms) for constant-temperature simulations and a 16 × 16 × 16 pseudocubic supercell (49,152 atoms) for temperature-ramp simulations, where the smaller cell was used to increase throughput. The MD time step is 1 fs throughout. Production runs are performed on a single NVIDIA A100 GPU, yielding a typical performance of approximately 0.42 ns/day.

The initial structures of FAPbI_3_ are constructed with an ideal *a* ^0^ *a* ^0^ *a* ^0^ inorganic framework. Preliminary tests show that FA orientations relax toward a ⟨100⟩ manifold, and all production simulations reported in the main text therefore start from initial molecular configurations chosen within this orientational basin; the precise construction of these initial states is described in Sections S7 and S10 of the Supporting Information.

The temperature-resolved structural dynamics of FAPbI_3_ are sampled using independent constant-temperature MD simulations starting from these initial structures, rather than by continuing a single cooling trajectory from higher temperature. Each system is first equilibrated for 200 ps in the *NpT* ensemble at the target temperature, followed by 50 ps in the *NVT* ensemble. Production data are then collected from a subsequent 200 ps *NVE* trajectory, with snapshots saved every 1 ps. Temperature and pressure are controlled using a Nosé–Hoover thermostat and barostat with damping constants of 100 and 200 fs, respectively.

Heating and cooling protocols are initialized from the final snapshot of an equilibrated trajectory and are propagated under a temperature ramp of 0.25 K/ps in the *NpT* ensemble. After the temperature ramp, each system is further evolved using the same *NVT* + *NVE* sequence described above. To ensure statistical robustness, independent trajectories with distinct initial conditions were used where required.

### 4.3 Electronic Structure and Disorder Analysis

Electronic structure calculations were performed on 2 × 2 × 2 subcells extracted directly from representative MD snapshots of FAPbI_3_. The atomic positions and lattice parameters of these subcells were taken from the MD configurations without further structural relaxation, so that the calculated band structures reflect the instantaneous local environments generated by the finite-temperature trajectories. Band structures were computed using the projector-augmented-wave method as implemented in VASP, with the PBEsol exchange–correlation functional.^44^ A plane-wave cutoff of 500 eV, Gaussian smearing of 0.05 eV, and a Γ-centered 2 × 2 × 2 *k*-point mesh were used for all 2 × 2 × 2 subcells. The electronic self-consistency threshold was set to 10^–7^ eV. Wave functions were written during the band-path calculations and subsequently unfolded onto the primitive Brillouin zone using VASPKIT,^45^ following the effective-band-structure formalism.^46^ PBEsol was chosen for its balanced structural accuracy and computational tractability across the large number of subcell evaluations required. The electronic analysis therefore focuses on relative variations of local band-edge energies across distinct structural environments rather than on quantitative prediction of the experimental bulk gap.

To assess the influence of spin–orbit coupling, additional SOC-inclusive calculations were performed for 100 randomly selected 2 × 2 × 2 subcells from each of the 50, 100, and 200 K populations. The same atomic structures, electronic-structure settings, and reciprocal-space paths were used for the corresponding SOC and non-SOC calculations. The SOC wave functions were subsequently unfolded using the same VASPKIT workflow.

Electronic analyses were performed on a single MD snapshot at each temperature, corresponding to ∼500 distinct 2 × 2 × 2 subcells in total. For each 2 × 2 × 2 subcell, the valence band maximum (VBM), conduction band minimum (CBM), and band gap were extracted from the DFT band structure. To relate local electronic variation to local structure, the octahedron-averaged tilt norm *T*_norm_^mean^ was used as the structural descriptor.

To quantify band-tail broadening, the sampled band-edge energies were converted into normalized histogram-based density-of-states representations. The exponential tail near the band edge was fitted using the Urbach form^47,48^

1 $$D\left(E\right)\propto \exp\left(\frac{E-E_{\min}}{E_{U}}\right)$$

where *D*(*E*) is the density of states in the band-edge tail region, *E*_min_ is the minimum sampled band-edge energy, and *E*_*U*_ is the Urbach energy. Taking the logarithm yields

2 $$\ln D\left(E\right)=\frac{E-E_{\min}}{E_{U}}+C$$

so that

3 $$E_{U}={\left(\frac{d\;\ln D}{dE}\right)}^{-1}$$

Only the lowest-energy tail of each histogram was used in the regression, and the fitting uncertainty was estimated from the standard error of the fitted slope. This procedure yields separate Urbach energies associated with fluctuations of the conduction and valence band edges, *E*_*U*_^CBM^ and *E*_*U*_^VBM^. Because optical absorption depends on transitions between these states, an effective optical Urbach energy was estimated as

4 $$E_{U}^{opt}=\sqrt{{\left(E_{U}^{CBM}\right)}^{2}+{\left(E_{U}^{VBM}\right)}^{2}}$$

Additional details of the fitting procedure, uncertainty estimation, and comparison among alternative structural descriptors are provided in Section S11 of the Supporting Information.

### 4.4 Experimental Details

X-ray diffuse scattering measurements on FAPbI_3_ at 102 K were carried out at the I19–1 beamline at Diamond Light Source using X-rays of 12.9 keV. We performed a quenching procedure in which we set the cryostream to 102 K and directly inserted the sample into the cryostream from the room-temperature environment. Based on a lumped-capacitance estimate for a ∼100 μm crystal in a nitrogen cryostream (Biot number Bi ≪ 0.1), the effective cooling rate during the quench is approximately 200–500 K/s, roughly 10^3^–10^4^ times faster than a standard cryostream ramp. X-ray diffuse scattering measurements on FAPbI_3_ at 130 K were carried out on the I15 beamline at Diamond Light Source using X-rays of 72 keV. The sample was cooled to 130 K with a cooling rate of 3 K/min. In both experiments, each diffraction image was collected with a Pilatus2M detector (1679 × 1475 pixels, 172 × 172 μm^2^ pixel size). The single crystals, mounted on the goniometer using a cryo loop for intensity measurements, were coated with immersion oil type NVH and then transferred to the nitrogen stream generated by an Oxford Cryostream 800 series. CrysAlisPro was used for indexing, determination and refinement of the orientation matrix, and then the precession images were unwrapped using CrysAlisPro, with polarization correction. The images were generated such that the full reciprocal volume was recovered and loaded into HDF5 (.h5) files using the rspace3d Python package.^49^ Outlier rejection and symmetrization were also applied in the software to obtain the final data cube.

To connect the real-space analysis with experimentally accessible observables, we computed the dynamical structure factor *S*(**q**, ω) from the MD trajectories using the pynamic structure factor (psf) package.^6,21^ The reciprocal-space resolution is 1/18 r.l.u., and both X-ray and neutron signals were sampled over 0–5 r.l.u. with an energy resolution of approximately 0.05 meV. The *q*-integrated neutron spectra shown in Figure 3d were obtained by summing *S*(**q**, ω) over all sampled reciprocal-space vectors satisfying |**q**| < 3 r.l.u. The resulting spectra were normalized by their integrated intensity for comparison of spectral shape.

## Supplementary Material



## Data Availability

The PDynA package used in this work is open-source and available online at https://github.com/WMD-group/PDynA (DOI: 10.5281/zenodo.7948045). The computational datasets, input files, representative MD structures, and MLFF training data associated with this work are available from Zenodo at DOI: 10.5281/zenodo.21847778.

## Notes

Force field validation, data set construction, structural dynamics statistics, molecular orientation analysis, reciprocal-space analysis, electronic structure analysis, and additional figures and tables (PDF)
