# Supplementary material for: Kinetically Arrested Twin-Domain State in Formamidinium Lead Iodide
Source: J Am Chem Soc. 2026 Aug 12;148(33):35995–6005. doi: 10.1021/jacs.6c09938 (PMC13523686; doi:10.1021/jacs.6c09938)
Supplement: Supplementary file 1 [file ja6c09938_si_001.pdf]

# Supporting Information: Kinetically Arrested Twin-Domain State in Formamidinium Lead Iodide

Xia Liang[1,2,*], Milos Dubajic[3,*], Zezhu Zeng[4], Yang Lu[3], Johan Klarbring[5], Samuel D. Stranks[3], Aron Walsh[1]

[1]Department of Materials, Imperial College London, South Kensington Campus, London SW7 2AZ, UK

[2]School of Energy and Chemical Engineering, Ulsan National Institute of Science and Technology (UNIST), Ulsan 44919, Korea

[3]Department of Chemical Engineering and Biotechnology, University of Cambridge, Philippa Fawcett Drive, Cambridge CB3 0AS, UK

[4]Physical and Theoretical Chemistry Laboratory, Department of Chemistry, University of Oxford, Oxford OX1 3QZ, UK

[5]Department of Physics, Chemistry and Biology (IFM), Linköping University, SE-581 83, Linköping, Sweden

# 1 Force Field Validation

Table S1: Root mean squared errors of energy, forces and stress tensors of the unified MLFF covering the chemical configuration space $(Cs_{1-x-y}MA_xFA_y)Pb(Br_mI_{1-m})_3$.

| Material | RMSE on test set | | |
|---|---|---|---|
| | Energy (meV/atom) | Force (meV/Å) | Stress (meV/Å$^3$) |
| $CsPbBr_3$ | 0.46 | 9.14 | 0.343 |
| $CsPbI_3$ | 0.42 | 11.15 | 0.163 |
| $MAPbBr_3$ | 1.42 | 16.53 | 0.219 |
| $MAPbI_3$ | 0.68 | 16.41 | 0.275 |
| $FAPbBr_3$ | 1.14 | 18.73 | 0.225 |
| $FAPbI_3$ | 1.04 | 19.10 | 0.366 |

The errors in Table S1 indicate that the unified MLFF retains near-DFT fidelity across all six halide-perovskite end members. In particular, the energy RMSE remains below 1.5 meV/atom and the force RMSE below 20 meV/Å for every composition, which places the model well within the accuracy regime typically regarded as high quality for MLFFs of soft ionic solids. These values are comparable to, and in several cases lower than, those reported for recent MLFFs trained on chemically related perovskite systems[1–3]. The small spread across compositions further suggests that the unified model does not sacrifice transferability to achieve accuracy for a single compound.

Fig. S1 shows parity plots for 200 MD snapshots drawn from the cubic-phase regimes of $CsPbI_3$, $MAPbI_3$, and $FAPbI_3$, with DFT energies, forces, and stresses recomputed using the same settings as in the data-generation workflow. The resulting errors remain at the same overall level as those obtained on the held-out test sets, confirming that the model remains accurate on representative structures sampled during production MD rather than only on the train/test partition.

A second validation is provided by the energy–volume and hydrostatic-stress–volume

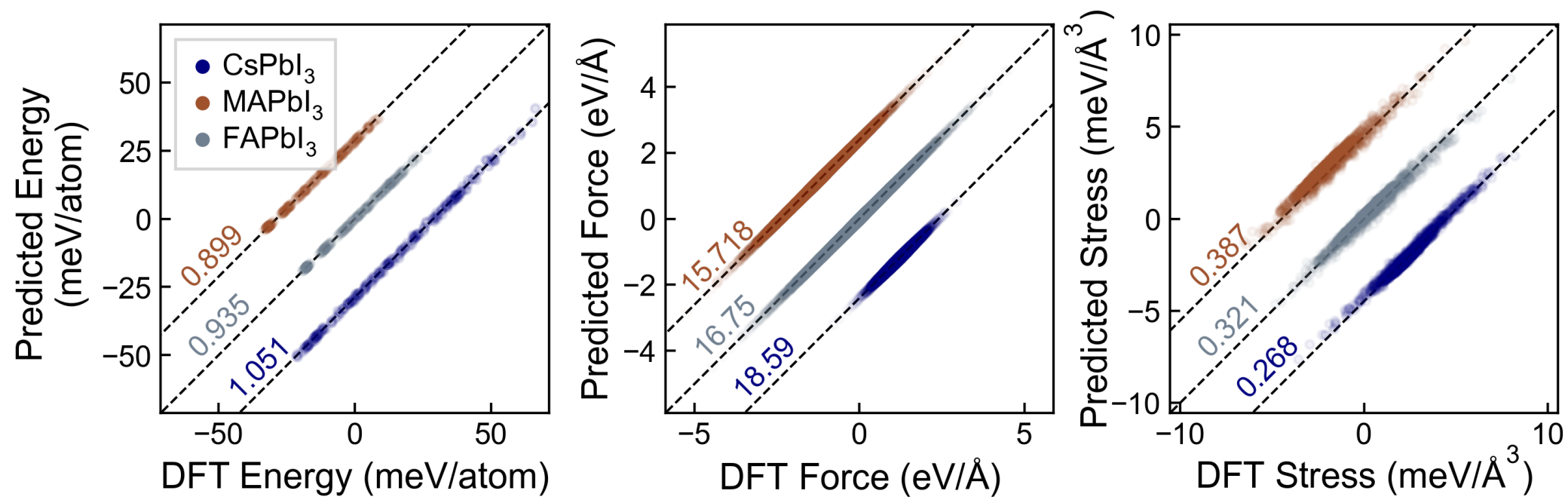


Figure S1: Parity plots for MLFF predictions on 200 MD snapshots drawn from the cubic-phase regimes of $CsPbI_3$, $MAPbI_3$, and $FAPbI_3$, with DFT reference values recomputed using the same settings as in the data-generation workflow. Left: energies; middle: forces; right: stresses. For visual clarity, the $CsPbI_3$ and $MAPbI_3$ populations are shifted relative to $FAPbI_3$, and separate parity lines are shown for each material. In the energy panel, the values are additionally shifted to a common scale because $CsPbI_3$ has a larger energy per atom. The numbers printed next to each population denote the corresponding RMSE values in meV units (meV/atom for energy, meV/Å for forces and meV/Å$^3$ for stresses).

curves for $FAPbI_3$ in the cubic $a^0a^0a^0$ and ordered $a^+a^+a^+$ structures (Fig. S2). Over a broad isotropic volume range from 80% to 125% of the reference cell volume, the MLFF reproduces the DFT-computed energies and hydrostatic stresses closely for both structural branches. This agreement confirms that the model captures not only local forces but also the near-equilibrium curvature and relative mechanical response of distinct structural states relevant to the present study.

To assess the lattice-dynamical response, Fig. S3 compares MLFF and DFT phonon dispersions of $CsPbI_3$ in its $\alpha$, $\beta$, and $\gamma$ phases. The agreement is consistently good across all three structures, showing that the unified MLFF reproduces the inorganic-framework lattice dynamics of this endpoint system with high fidelity. We do not use an analogous harmonic-phonon benchmark as a primary validation for $FAPbI_3$, because in the molecular iodide perovskite the orientational disorder of the FA sublattice and the resulting strong anharmonicity make a single-reference harmonic dispersion less uniquely defined than in the inorganic endpoint.

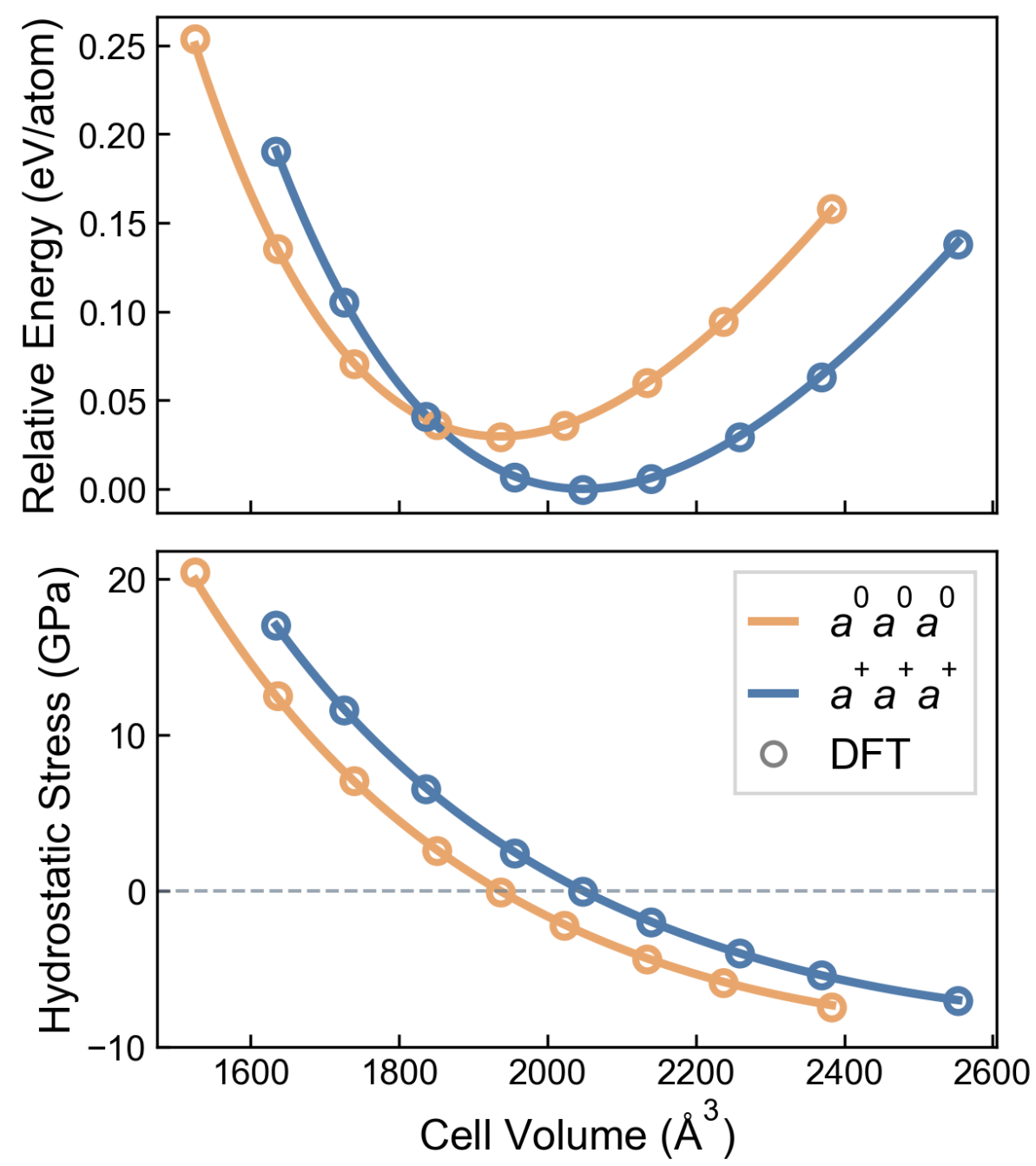


Figure S2: Energy–volume and hydrostatic-stress–volume relations for $FAPbI_3$ along the cubic $a^0a^0a^0$ and ordered $a^+a^+a^+$ structural branches. Solid lines show MLFF predictions over an isotropic volume range from 80% to 125% of the reference cell volume, and open circles denote DFT reference calculations on selected structures.

Finally, finite-temperature structural fidelity is tested by comparing DFT and MLFF radial distribution functions for $FAPbI_3$ at 100 and 300 K (Fig. S4). We focus on three representative pairs that capture the essential local physics: Pb–I for nearest-neighbour octahedral bonding, I–I for framework geometry and tilt-sensitive halide separations, and N–I for coupling between the FA cation and the inorganic cage. The MLFF reproduces all three distributions well at both temperatures, with only small differences in the first Pb–I peak. Taken together, these tests indicate that the unified potential reproduces the static, vibrational, mechanical, and finite-temperature structural observables most relevant to the large-scale MD analyses presented in the main text.

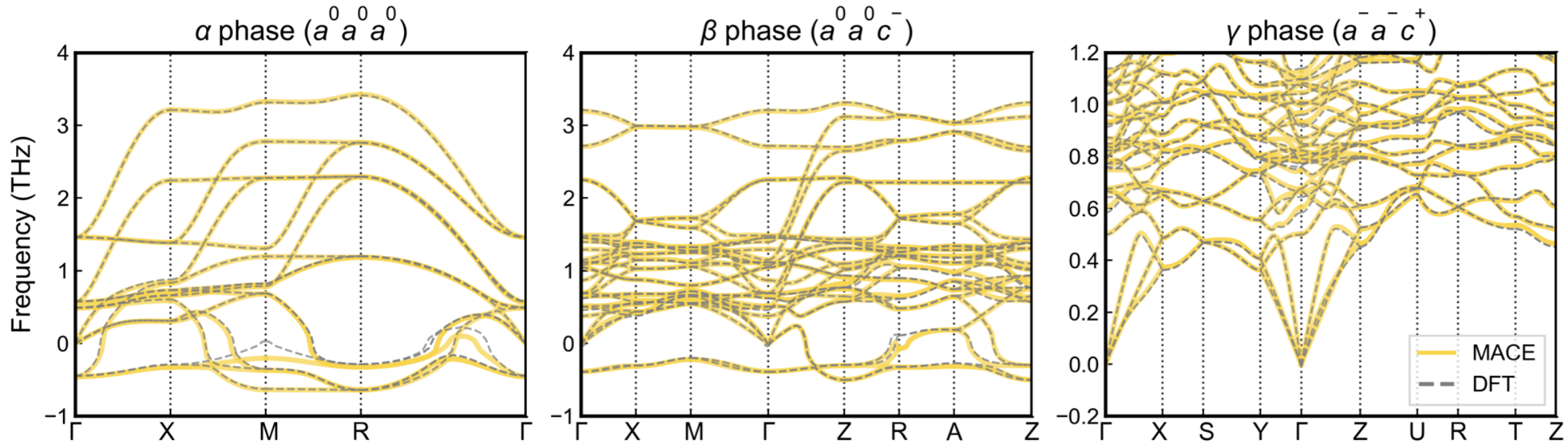


Figure S3: Comparison of MLFF and DFT phonon dispersions of $CsPbI_3$ in the $\alpha$ ($a^0a^0a^0$), $\beta$ ($a^0a^0c^-$), and $\gamma$ ($a^-a^-c^+$) phases. The unified MLFF reproduces the DFT lattice-dynamical spectra well across all three structures.

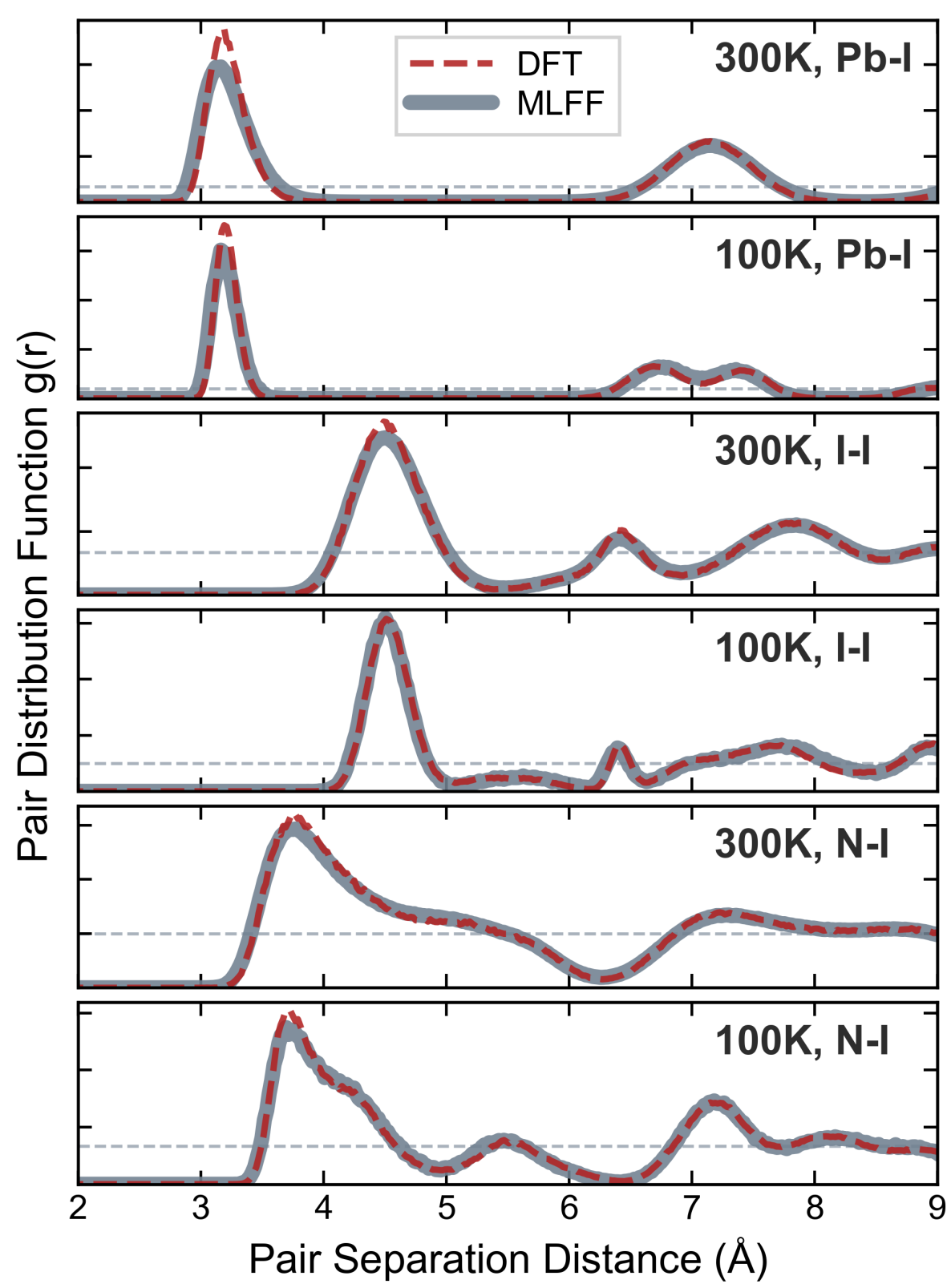


Figure S4: Radial distribution functions of $FAPbI_3$ at 100 K (ordered $a^+a^+a^+$) and 300 K (cubic $a^0a^0a^0$) from DFT and MLFF trajectories. The Pb–I, I–I, and N–I pair correlations are shown.

## 2 Pulay Stress and Recomputed DFT Labels

During variable-cell *ab initio* MD, the simulation cell changes continuously, whereas the plane-wave basis is defined through a finite cutoff. In plane-wave DFT, this can introduce Pulay-stress errors because the basis is not fully complete with respect to changes in cell shape and volume, leading to systematic errors in energies and especially stresses if the instantaneous configurations are used directly as training labels[4]. This issue is well known for cell relaxations and variable-cell simulations in VASP, where Pulay stress can bias the calculated stress tensor and distort the equilibrium volume unless the basis is sufficiently converged or the structures are recomputed with the updated basis[5].

To suppress this source of bias, all structures selected for final training were recomputed with static single-point DFT calculations using the converged labeling setup described in the Methods. This produced a recomputed dataset (*reDFT*) with substantially reduced basis-set inconsistency relative to the raw variable-cell data obtained directly from on-the-fly trajectories. Fig. S5 illustrates the effect for $MAPbI_3$: relative to a model trained on the raw labels, retraining on the recomputed labels removes the systematic shift in the energy and stress prediction and reduces the corresponding error distribution. In practice, this correction is particularly important for large-scale production MD, where even a modest systematic stress bias can accumulate into an artificial contraction or expansion of the simulation cell.

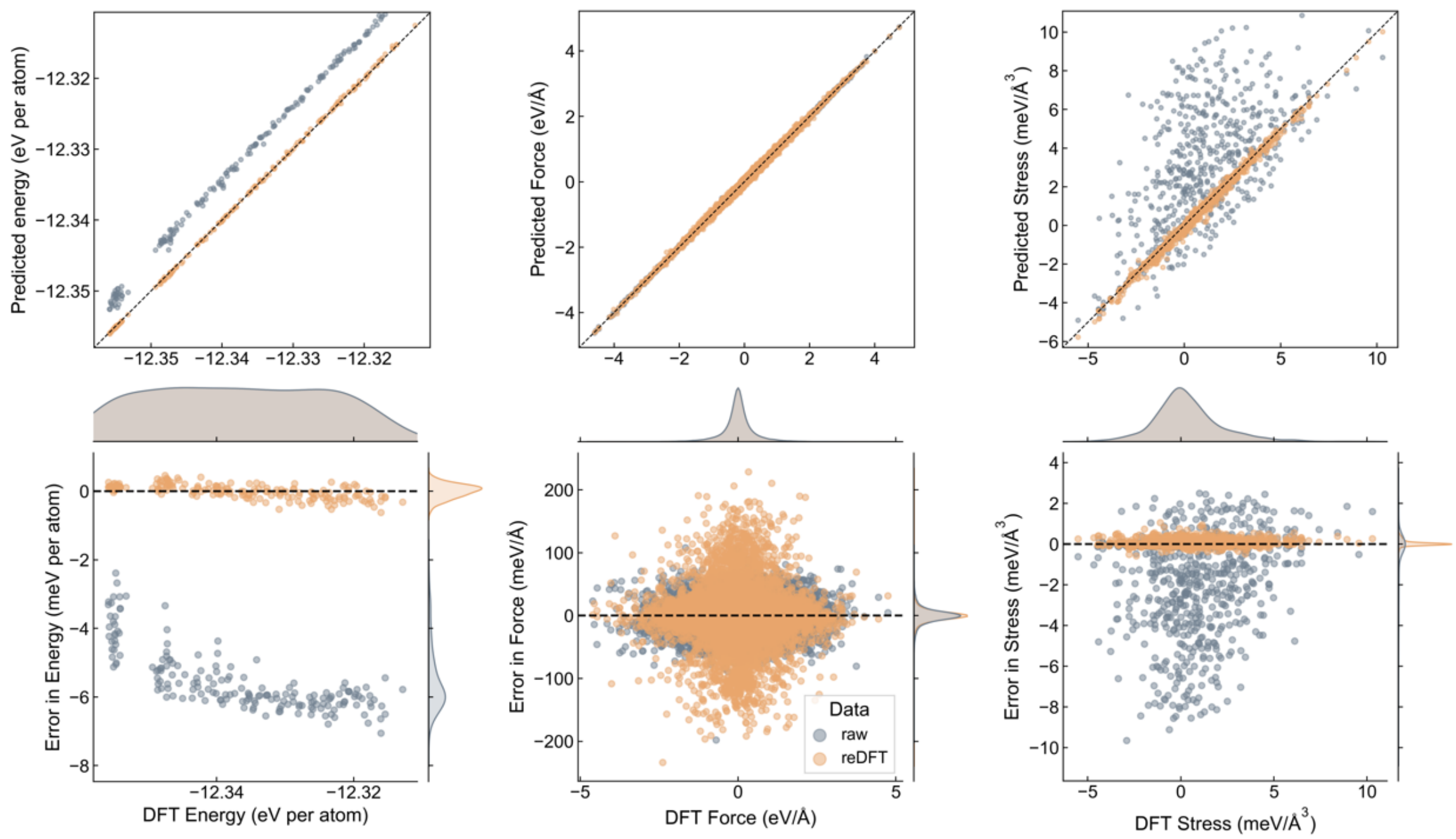


Figure S5: Comparison of $MAPbI_3$ validation errors obtained from MLFFs trained on raw on-the-fly labels and on recomputed single-point DFT labels (*reDFT*). Top row: predicted versus DFT values for energy, forces, and stress. Bottom row: corresponding prediction errors. The recomputed dataset removes the dominant systematic bias in the stress prediction and improves the consistency of the training labels.

# 3 Compositional Sampling Strategy for Surrogate Molecular Dynamics

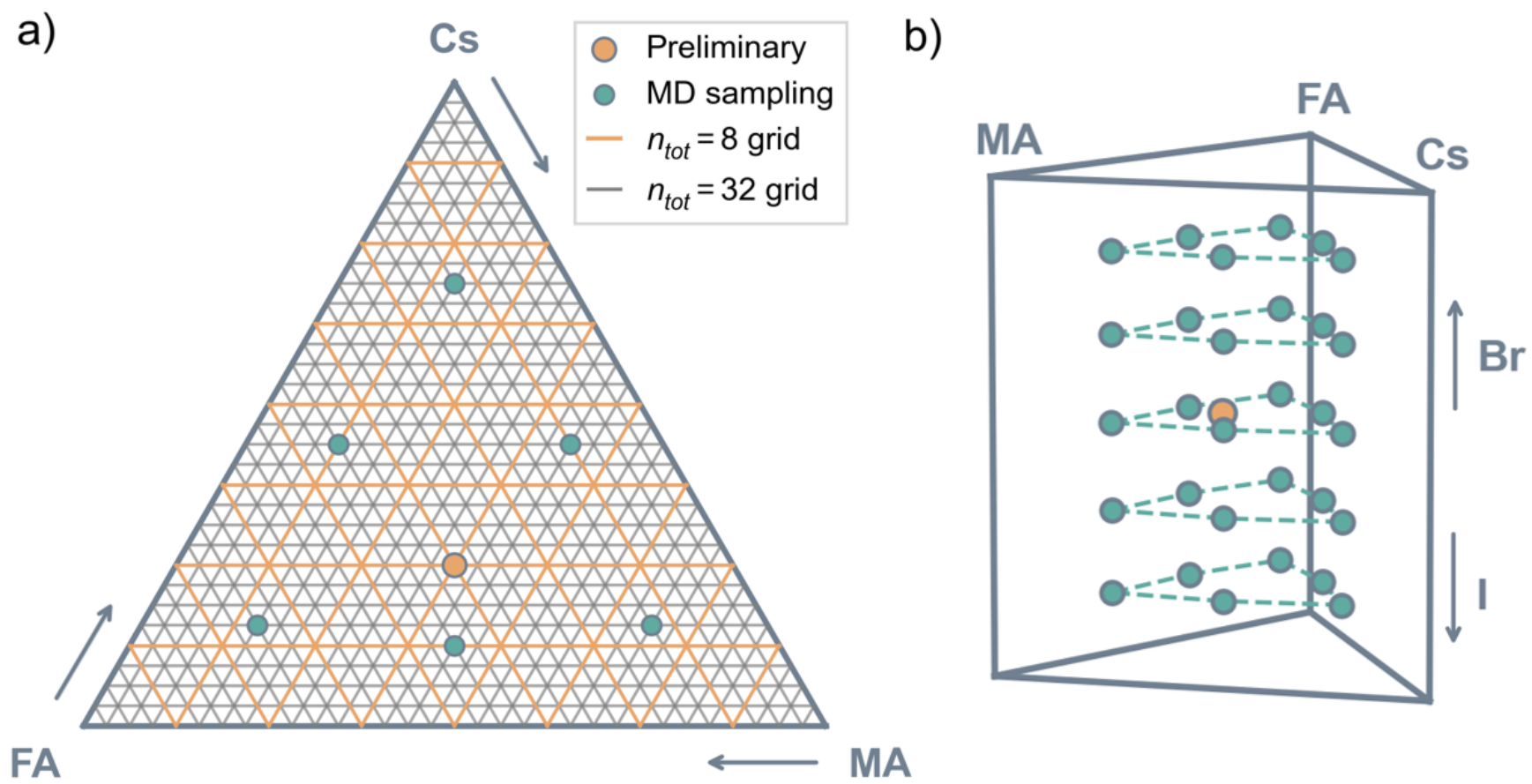


Figure S6: Sampling of the doubly mixed lead-halide perovskite composition space. (a) A-site ternary composition diagram showing the preliminary on-the-fly training composition and the set of A-site profiles used for surrogate MD sampling. The overlaid coarse and fine grids illustrate the $n_{\mathrm{tot}} = 8$ and $n_{\mathrm{tot}} = 32$ composition discretisations. (b) Schematic three-dimensional representation of the doubly mixed space spanned by the A-site and halide mixing axes, showing the preliminary composition and the mixed compositions sampled in the surrogate-MD stage.

To sample the doubly mixed compositional space $(\mathrm{Cs}_{1-x-y}\mathrm{MA}_x\mathrm{FA}_y)\mathrm{Pb}(\mathrm{Br}_m\mathrm{I}_{1-m})_3$ with the surrogate MLFF, we introduce two independent mixing axes: the A-site sublattice and the halide sublattice. On the A-site sublattice, seven cation profiles $(n_{\mathrm{FA}}, n_{\mathrm{MA}}, n_{\mathrm{Cs}})$ are selected such that $n_{\mathrm{FA}}+n_{\mathrm{MA}}+n_{\mathrm{Cs}} = 32$, namely $(11, 11, 10)$, $(5, 5, 22)$, $(14, 14, 4)$, $(4, 14, 14)$, $(5, 22, 5)$, $(22, 5, 5)$, and $(14, 4, 14)$. On the halide sublattice, 15, 31, 48, 65, or 81 bromine atoms are replaced by iodine using ICET, giving five mixed-halide compositions. Combining these two sets yields 35 distinct mixed structures, which are then paired with five idealised octahedral tilt patterns to generate the surrogate-MD dataset described in the Methods. The temperatures are chosen such that the Br-rich compositions are sampled at lower temperatures than the corresponding I-rich

compositions, consistent with the systematically lower phase transition temperatures of bromide-rich lead perovskites.

Table S2: Idealised initial tilt patterns and the corresponding temperature sets used for surrogate-MD sampling. Br-rich compositions are assigned lower temperatures than the corresponding I-rich compositions, consistent with the lower transition temperatures of bromide-rich lead perovskites.

| Tilting mode | Temperatures (K) |
|---|---|
| $a^0a^0a^0$ | 200, 300, 350, 400, 450 |
| $a^0a^0c^-$ and $a^0a^0c^+$ | 120, 150, 180, 210, 240 |
| $a^-a^-c^+$ and $a^+a^+a^+$ | 40, 55, 70, 85, 100 |
| **Temperatures (K)** | **Halide composition** |
| 200/120/40 | $I_{15}Br_{81}$ |
| 300/150/55 | $I_{31}Br_{65}$ |
| 350/180/70 | $I_{48}Br_{48}$ |
| 400/210/85 | $I_{65}Br_{31}$ |
| 450/240/100 | $I_{81}Br_{15}$ |

The surrogate-sampling workflow generates 35,000 candidate structures from small-cell MD across the doubly mixed composition space. 3,000 structures were then selected by DIRECT for final DFT relabelling. Fig. S7 shows that the surrogate-generated structures span a substantially broader region of SOAP descriptor space than the initial on-the-fly dataset obtained from the single reference composition $Cs_2MA_3FA_3Pb_8Br_{12}I_{12}$. The initial on-the-fly structures therefore act as a chemically representative starting point near the centre of the targeted configuration space, while the surrogate model enables systematic exploration into a much wider range of local environments.

Within the surrogate-generated dataset, PCA of the SOAP descriptors reveals a clear hierarchical organisation of the local environments (Fig. S8). The strongest separation is by A-site composition, indicating that changes in the relative fractions of FA, MA,

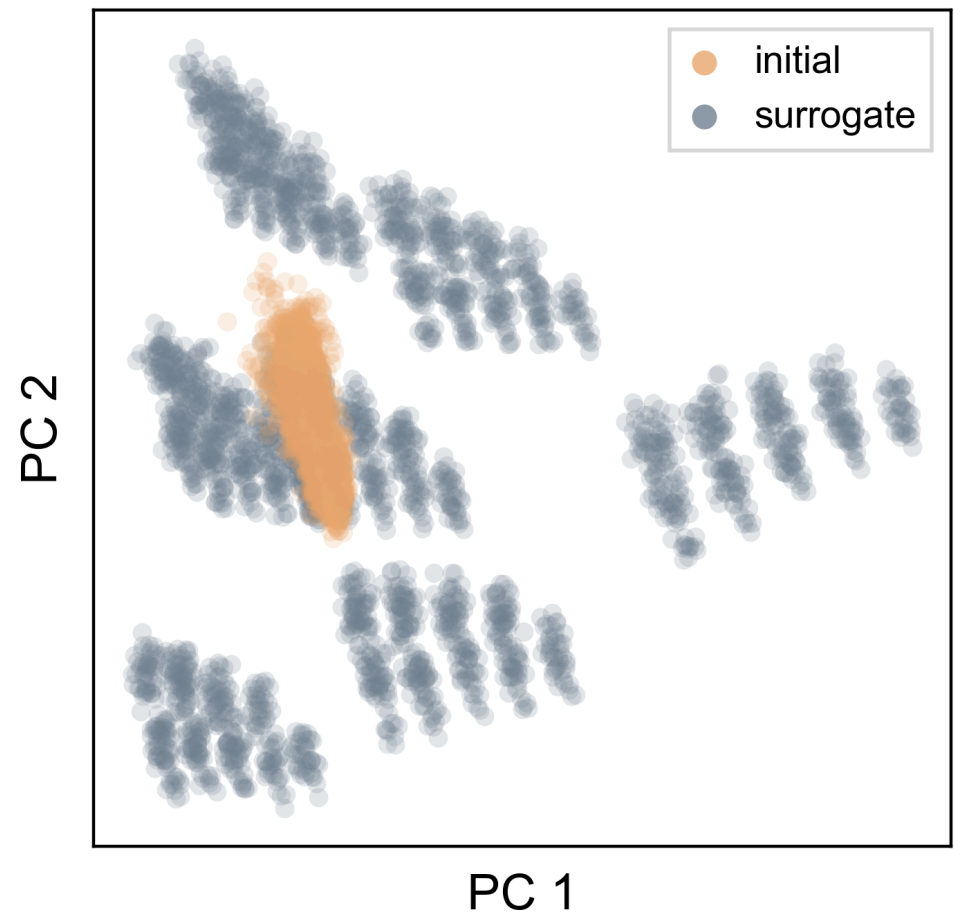


Figure S7: PCA projection of SOAP descriptors for structures obtained from the initial on-the-fly dataset and from the surrogate-MD sampling workflow. The surrogate-generated structures span a substantially broader region of descriptor space, showing that the initial dataset provides a chemically representative seed while the surrogate model expands the accessible local-environment diversity.

and Cs dominate the first layer of structural diversity. Within each A-site cluster, halide composition produces further subdivision into narrower strands, consistent with systematic sampling along the Br–I mixing direction. Temperature then generates an additional progression within each compositional strand, reflecting the increasing diversity of local environments sampled at higher thermal energy. This separation supports the use of a structured surrogate-MD workflow rather than a purely random exploration of configuration space.

The selected 3,000-frame training subset is not distributed uniformly across all categories (Fig. S9). The A-site profiles remain comparatively balanced, whereas the selected structures are skewed towards I-rich compositions and towards higher temperatures. This bias is physically reasonable: I-rich systems and high-temperature trajectories exhibit stronger structural rearrangements and broader local environment diversity, and therefore contribute disproportionately to the set of distinct configurations retained by DIRECT.

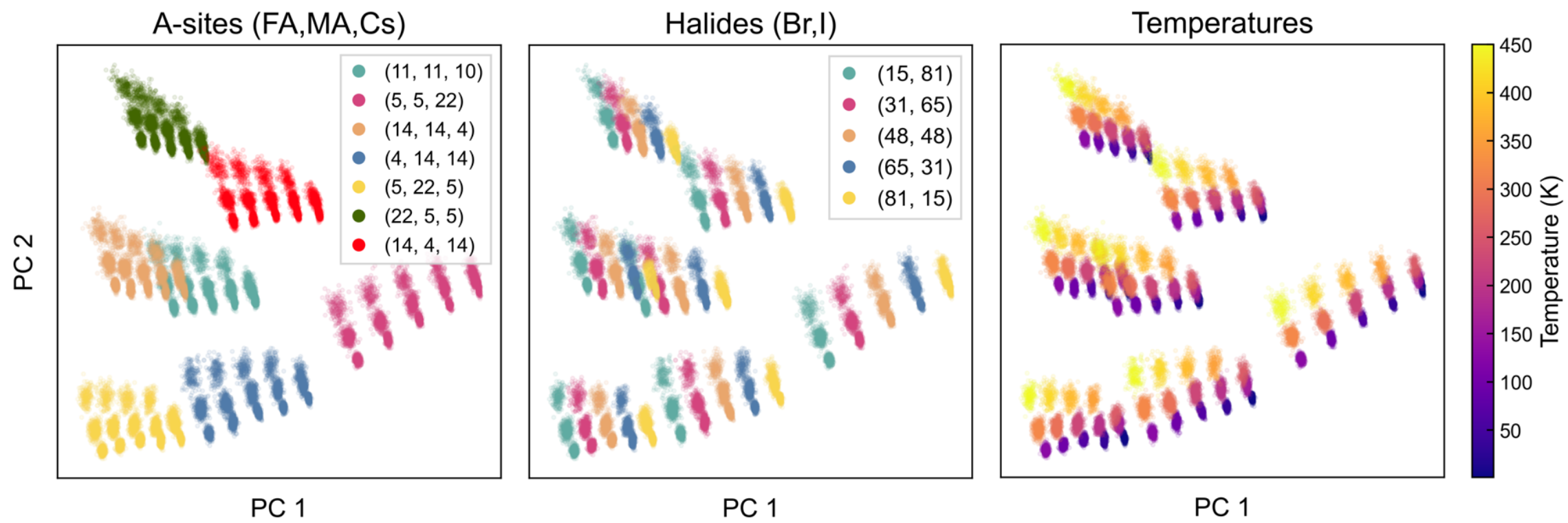


Figure S8: PCA projection of SOAP descriptors for the surrogate-generated doubly mixed perovskite structures, coloured by A-site composition, halide composition, and temperature. The descriptor space exhibits a hierarchical clustering in which A-site composition produces the strongest separation, followed by halide composition and then temperature.

To further improve sampling near the boundaries of the chemical space, the final training set also includes endpoint-family data for $CsPbX_3$, $MAPbX_3$, and $FAPbX_3$, each contributing 1000 additional structures. The effect of this endpoint-family augmentation is shown in Fig. S10. Relative to training on the mixed-composition *reDFT* dataset alone, the *reDFT+EP* model yields consistently improved errors across most validation sets. This improvement is especially clear for the energy and stress predictions, showing that explicit coverage of the edges of the A-site/halide composition space materially improves transferability within the interior of the doubly mixed space. This behaviour is consistent with the general aim of selection, namely to construct a training dataset that captures structural and chemical diversity across a large configuration manifold.

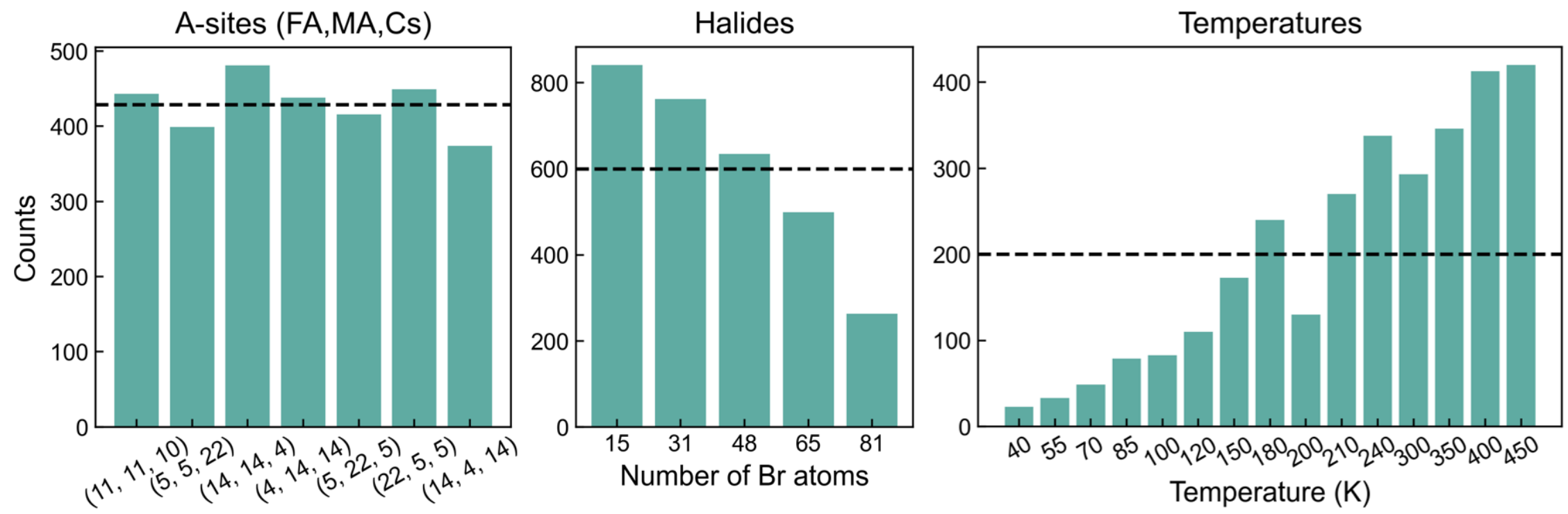


Figure S9: Number of DIRECT-selected structures across A-site compositions, halide compositions, and simulation temperatures. Dashed lines indicate the counts expected from a purely uniform selection. The selected dataset remains broadly balanced across A-site compositions but is enriched in I-rich and high-temperature structures, reflecting their greater structural diversity.

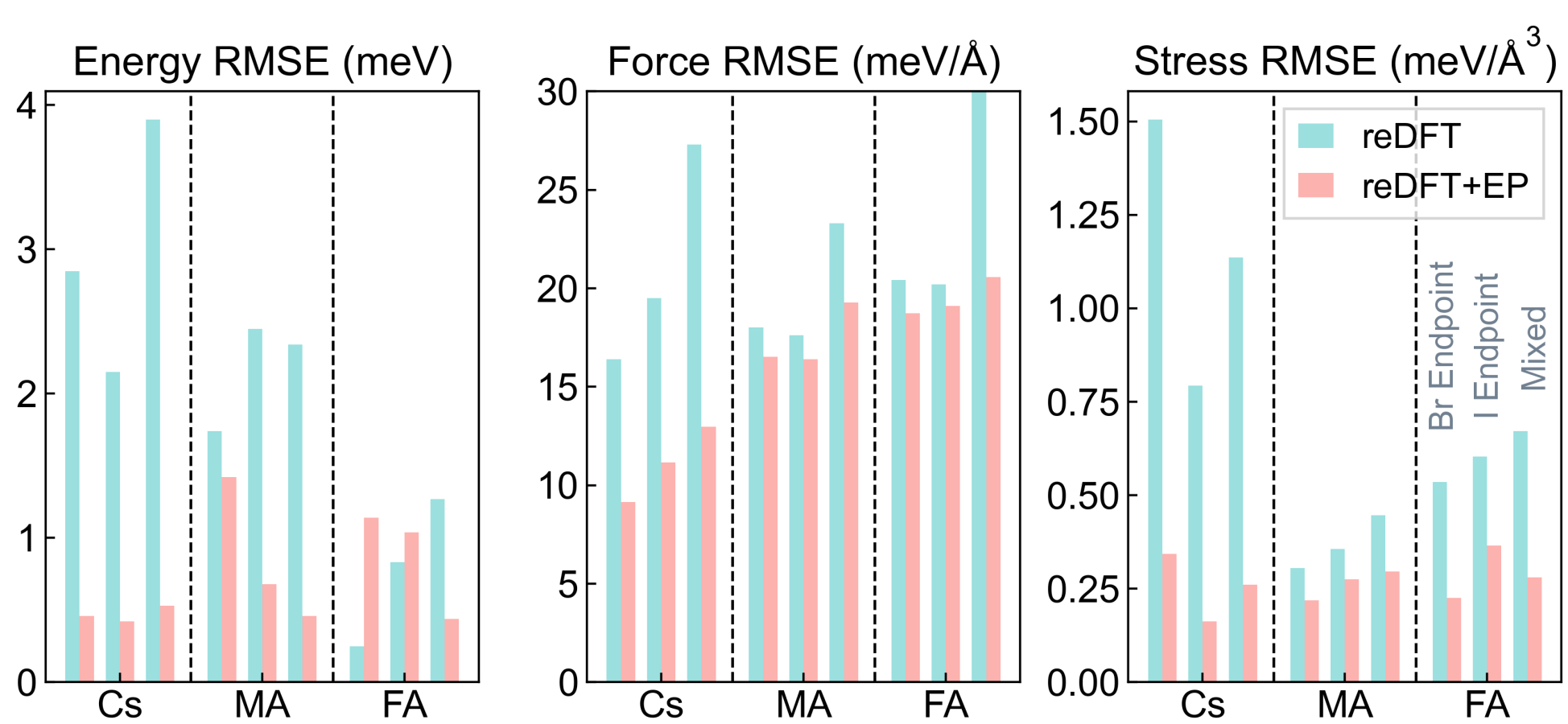


Figure S10: Validation RMSEs of models trained on the mixed-composition *reDFT* dataset alone and on the *reDFT+EP* dataset including endpoint-family structures. Results are grouped by A-site family and by Br endpoint, I endpoint, and mixed-halide validation sets. Including endpoint-family data improves predictive accuracy across most target regions of the doubly mixed chemical space.

# 4 Structural Dynamics Statistics

Following the PDynA framework[6], each $PbX_6$ octahedron is assigned a local tilt angle $\theta_\alpha(t;\mathbf{n})$ around axis $\alpha \in \{x, y, z\}$, where $\mathbf{n} = (n_x, n_y, n_z)$ indexes the octahedron in the supercell,

$$\theta_\alpha(t;\mathbf{n}). \tag{1}$$

The three components together define the local instantaneous tilting state of an octahedron. In practice, the mean tilting magnitude shown in Fig. S11a is extracted separately for each crystallographic direction from the corresponding distribution of instantaneous tilt angles. For each $\alpha \in \{x, y, z\}$, the sampled distribution is fitted to a symmetric two-Gaussian form,

$$P_\alpha(\theta) = A_\alpha \exp\left[-\frac{(\theta - \langle T_\alpha\rangle)^2}{2\sigma_\alpha^2}\right] + A_\alpha \exp\left[-\frac{(\theta + \langle T_\alpha\rangle)^2}{2\sigma_\alpha^2}\right], \tag{2}$$

where $\langle T_\alpha\rangle$ is the fitted mean tilt magnitude about axis $\alpha$, and $\sigma_\alpha$ is the common width of the two peaks. This definition follows the use of Gaussian fitting to the dynamic tilt distributions in the original PDynA analysis.

To quantify the local sign correlation of the tilt pattern, we use the nearest-neighbour correlation descriptor introduced in PDynA. For tilting around axis $\alpha$, correlated along axis $\beta$, the sign-sensitive local correlation with the $k$-th neighbour is

$$r^{(k)}_{\alpha,\beta}(t;\mathbf{n}) = \frac{\theta_\alpha(t;\mathbf{n})\,\theta_\alpha(t;\mathbf{n}+k\hat{\beta})}{\sqrt{\left|\theta_\alpha(t;\mathbf{n})\,\theta_\alpha(t;\mathbf{n}+k\hat{\beta})\right|}}, \tag{3}$$

which is effectively the sign of the tilt-product whenever both tilts are non-zero. Positive and negative values correspond to in-phase and out-of-phase local correlations, respectively. For the special case $\alpha = \beta$ and $k = 1$, this quantity directly reflects the

first-neighbour Glazer correlation along the tilt direction.

The corresponding global spatial tilting correlation function is

$$R_{\alpha,\beta}(k) = C \left\langle \theta_\alpha(t;\mathbf{n})\, \theta_\alpha(t;\mathbf{n}+k\hat{\beta}) \right\rangle_{\mathbf{n},t,\pm k}, \tag{4}$$

where $C$ is chosen so that $R_{\alpha,\beta}(0) = 1$, and $\langle \cdots \rangle_{\mathbf{n},t,\pm k}$ denotes averaging over all octahedra, all sampled times, and neighbours at $\pm k$ along $\beta$. The spatial extent of the tilt correlation is obtained by fitting the envelope of $R_{\alpha,\beta}(k)$ to

$$R_{\alpha,\beta}(k) = \exp\left(-\frac{k}{\xi_{\alpha,\beta}}\right), \tag{5}$$

where $\xi_{\alpha,\beta}$ is the fitted correlation length in the $\beta$ direction of the tilt around the $\alpha$ axis. In the main text, the real-space correlation lengths are reported as the relevant directional values.

For the controlled-orientation simulations discussed in Fig. 3c, the low-temperature structural outcomes were classified according to the number of crystallographic directions exhibiting system-spanning tilt correlation. In practice, a direction was labelled as system-spanning when the fitted real-space correlation length exceeded 18 pseudocubic unit cells, corresponding to the full linear size of the simulation box used in that analysis. No divergence of the fitted correlation length is implied. This criterion distinguishes fully correlated single-domain $\gamma$-like configurations from heterogeneous low-temperature $\gamma'$ states with reduced long-range coherence. The corresponding fitted correlation lengths for all controlled-orientation simulations are shown explicitly in Fig. S12, which provides the underlying structural dataset used to classify the outcomes summarised in Fig. 3c.

The tilting correlation polarity (TCP) around axis $\alpha$ is defined from the populations

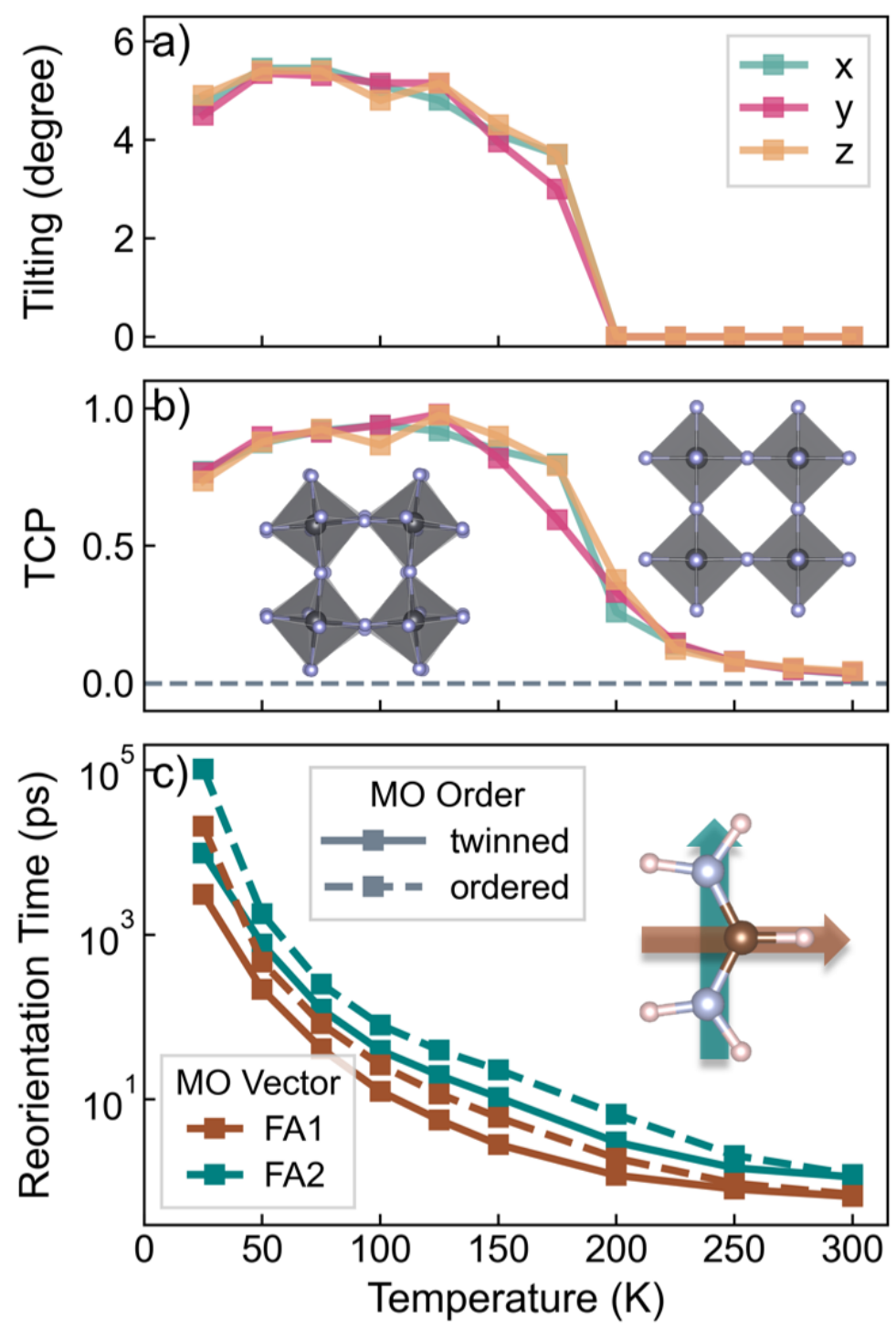


Figure S11: Temperature dependence of (a) the fitted mean tilt magnitude along the three crystallographic directions, extracted from the two-Gaussian fit of the dynamic tilt-angle distributions; (b) the TCP along the three crystallographic directions; and (c) characteristic FA molecular reorientation times extracted from the molecular-orientation autocorrelation functions.

of positive and negative first-neighbour correlations,

$$\delta_\alpha = \frac{n_\alpha^+ - n_\alpha^-}{n_\alpha^+ + n_\alpha^-}, \tag{6}$$

where $n_\alpha^+$ and $n_\alpha^-$ count the instances of $r_{\alpha,\alpha}^{(1)} > 0$ and $r_{\alpha,\alpha}^{(1)} < 0$, respectively. As a result, $\delta_\alpha \in [-1, 1]$, with values close to 1, 0, and $-1$ corresponding to Glazer superscripts $+$, 0, and $-$, respectively.

Time correlations are analysed for both octahedral tilting and molecular orientations. In both cases, the normalised autocorrelation functions are fitted to the same

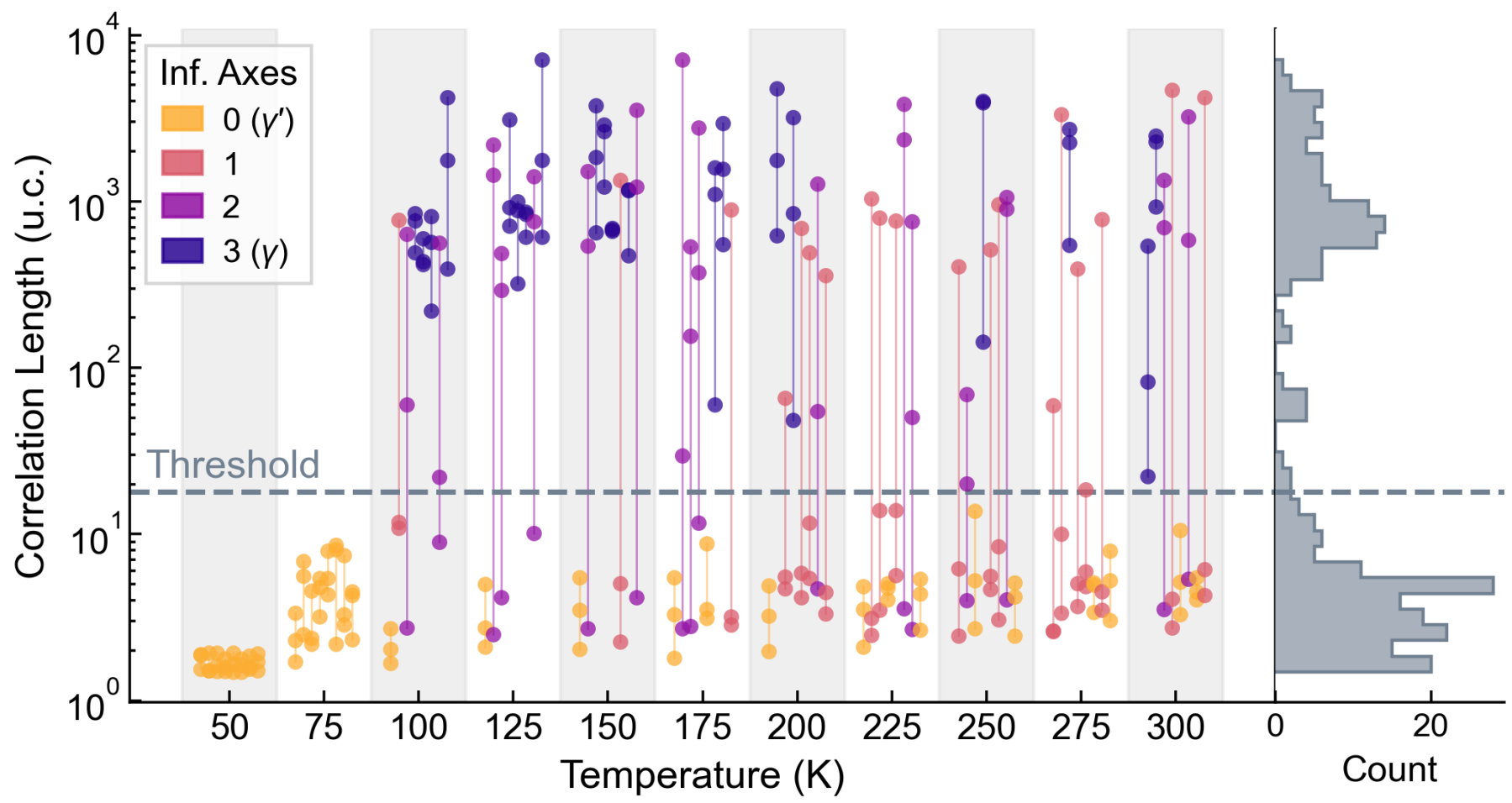


Figure S12: Temperature-dependent correlation length statistics for the controlled-orientation simulations, coloured by the number of system-spanning axes. Each vertical cluster shows the three directional correlation lengths ($x$, $y$, and $z$) from a single MD run at the indicated temperature, with repeat runs spread symmetrically around each temperature tick. Point colour encodes the number of system-spanning axes identified for that run: 0 ($\gamma'$), 1, 2, or 3 ($\gamma$), thereby indicating the degree of long-range octahedral tilt coherence. The dashed horizontal line marks the threshold of 18 pseudocubic unit cells used to distinguish finite from system-spanning correlation. The right panel shows the marginal distribution of all fitted correlation lengths on the same logarithmic scale.

biexponential form used in the original PDynA formalism,

$$A(t) = C \exp\left(-\frac{t}{\tau_1}\right) + (1 - C) \exp\left(-\frac{t}{\tau_2}\right), \tag{7}$$

where $\tau_1$ is the dominant correlation time and $\tau_2$ captures the short-time correction. In all fits considered here, $C > 0.8$, so that $\tau_1$ is the dominant timescale and is therefore taken as the characteristic tilt or molecular reorientation time, while the second exponential term accounts for the fast initial decay correction.

Fig. S11 summarises the temperature dependence of the fitted directional mean tilt angles, the corresponding TCP values, and the FA reorientation time. Panels a and b show not only the growth of $\gamma$-like in-phase tilt order on cooling from the high-temperature phase, but also its partial reduction at lower temperature as the

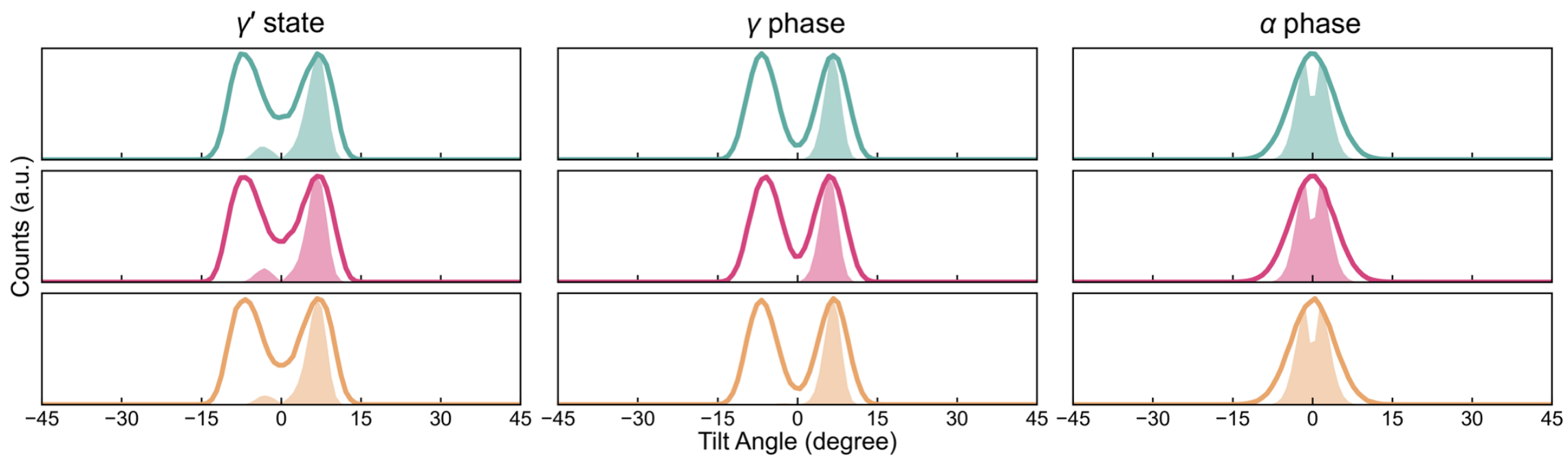


Figure S13: Explicit octahedral tilt angle distributions for the three key structural regimes of $FAPbI_3$: the high-temperature $\alpha$ phase, the ordered $\gamma$ phase, and the low-temperature $\gamma'$ state. Rows correspond to the three Cartesian tilt directions.

system crosses into the twinned $\gamma'$ state, where both the fitted tilt amplitude and TCP decrease. Panel c shows the rapid increase in molecular reorientation time on cooling, which forms part of the dynamical basis for the history-dependent crossover discussed in the main text. For completeness, Fig. S13 shows the explicit distributions of octahedral tilt angles for the three key structural regimes discussed in the main text, namely the high-temperature $\alpha$ phase, the ordered $\gamma$ phase, and the low-temperature $\gamma'$ state. In the $\alpha$ phase, the overall tilting is at zero degrees and with fluctuation up to 15 degrees, consistent with dynamic temporal local tilts. In the ordered $\gamma$ phase, the distributions sharpen into well-defined non-zero peaks, reflecting long-range in-phase tilt coherence. In the $\gamma'$ state, the distributions resemble those of the $\gamma$ phase but with finite out-of-phase correlation, which is attributed to the twin domain boundary. The corresponding tilt-autocorrelation analysis is shown separately in Fig. S14. The characteristic correlation time rises sharply on cooling. The extended-time curves further show that the 250 and 300 K autocorrelation functions decay to approximately zero within 100 ps, whereas those at 200 K and below retain substantial finite correlations throughout the displayed window. The characteristic correlation times obtained from ordered structures are consistently higher than those of their twinned counterparts until the values converge above 200 K.

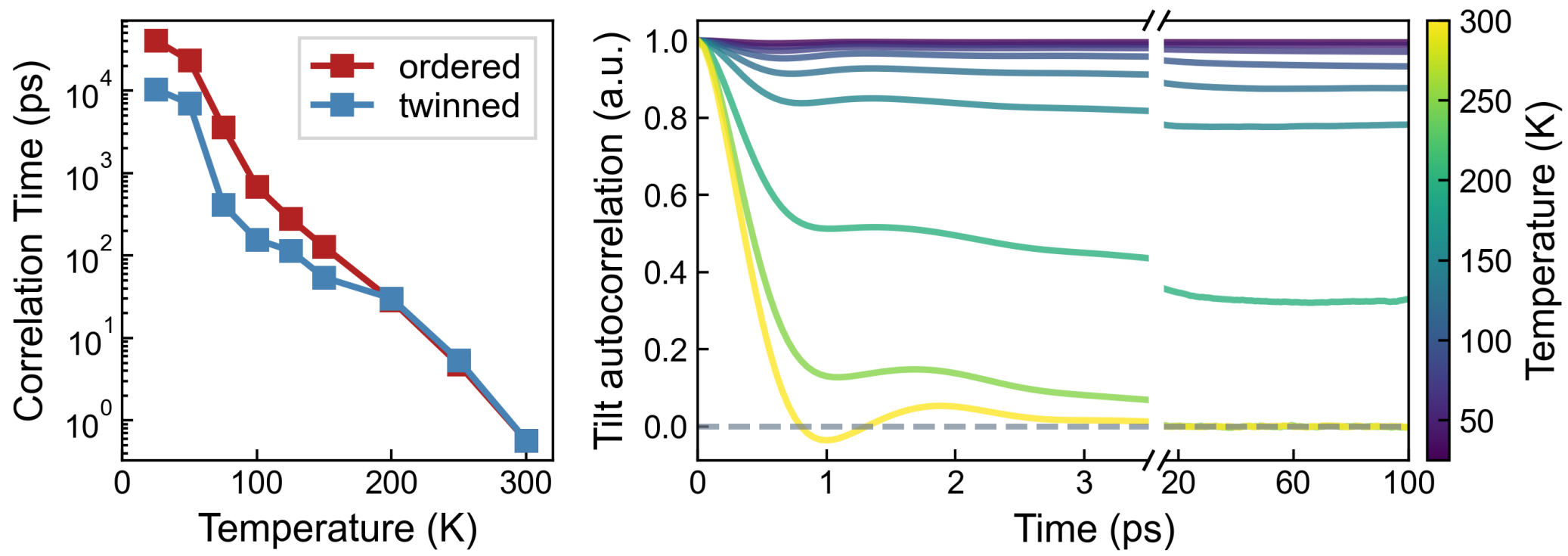


Figure S14: Tilt-autocorrelation analysis of $FAPbI_3$. The left panel shows the characteristic tilt correlation times extracted from the biexponential fit of Eq. 7 for ordered and twinned structures. The right panel shows the corresponding normalised tilt autocorrelation functions, averaged over the three Cartesian directions, using a broken time axis to display both the initial 0–3.5 ps decay and the extended 20–100 ps behaviour.

The extended-time view in Fig. S14 is included to show the relaxation behaviour underlying the characteristic times in the left panel. At 250 and 300 K, the tilt autocorrelation is effectively lost within the displayed 100 ps window. At lower temperatures, substantial residual correlation persists throughout the same interval, demonstrating increasingly long-lived tilt memory on cooling.

The tilt autocorrelation measures decorrelation of local framework fluctuations, whereas the molecular autocorrelation measures reorientation of FA molecular axes between preferred orientational basins. Fig. S15 compares the relative temperature-dependent slowing of the tilt and molecular degrees of freedom. The tilt and molecular quantities can be compared as characteristic relaxation times, but they monitor different microscopic observables. Around the $\gamma$-formation window, approximately 100–200 K, the fitted timescales are of the same broad order. On further cooling, all three correlation time ratios increase rapidly relative to their respective 150 K reference values, showing the shared slowing of framework decorrelation and molecular reorientation. Together with the controlled-orientation simulations in Fig. 3c and the energetic comparison in Fig. S20, this behaviour supports the assignment of the low-temperature $\gamma'$ state to

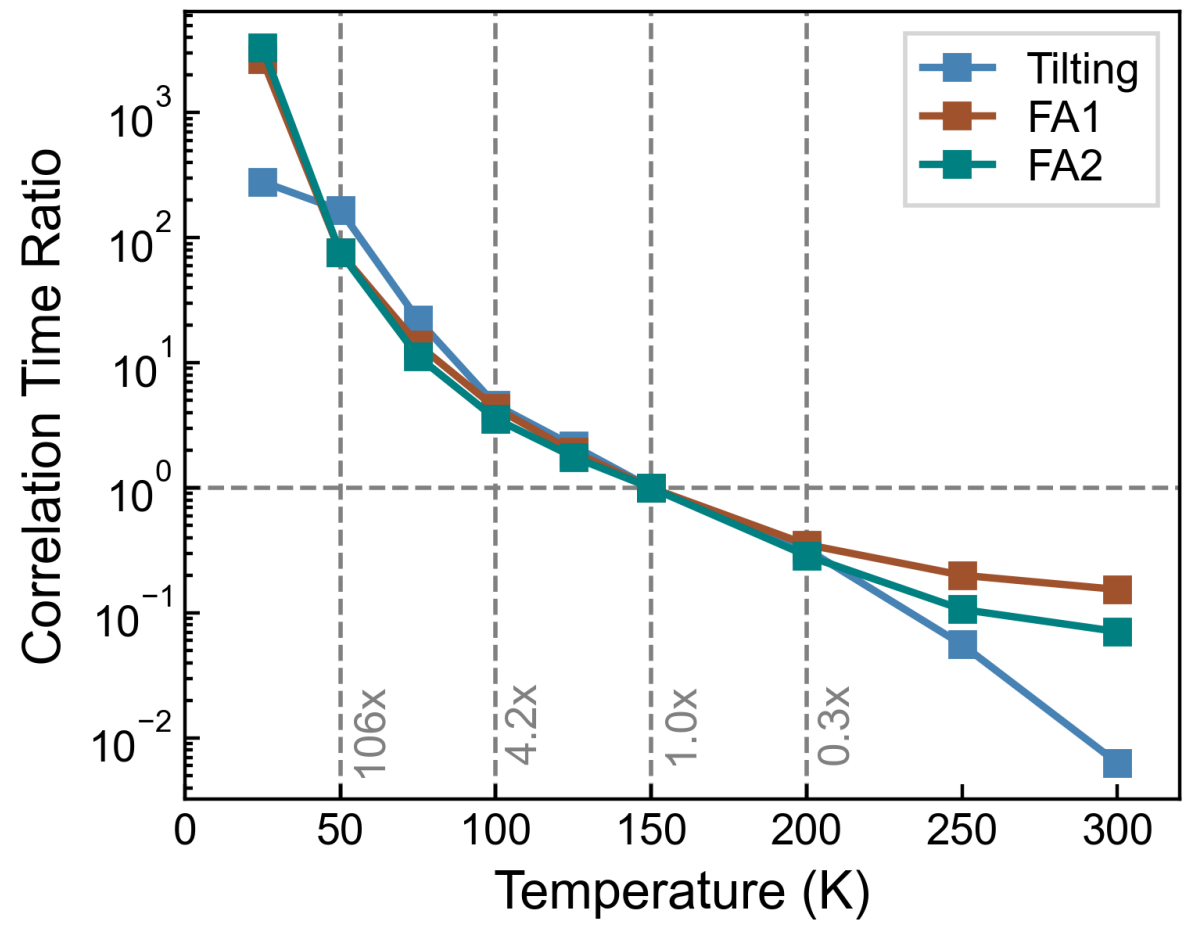


Figure S15: Relative slowing of octahedral tilting and FA molecular reorientation in $FAPbI_3$. The characteristic correlation times for octahedral tilting, FA1 reorientation, and FA2 reorientation are normalised to their respective values at 150 K. The vertical dashed lines mark selected temperatures, and the grey horizontal line indicates the 150 K reference value. The ratios show that both the framework and molecular degrees of freedom slow sharply on cooling below the $\gamma$-formation window.

kinetic arrest of the coupled ordering process rather than to a separate lower-energy phase.

## 5 Local Tilt Energetics and Bond-Length Coupling

The tilt-resolved potential energy surfaces discussed in the main text should be distinguished from finite-temperature free-energy landscapes. In anharmonic halide perovskites, free energy includes both entropic contributions and thermal averaging and is therefore required to determine equilibrium phase stability[7]. Here, by contrast, the potential energy surfaces are used more narrowly as mechanistic probes of the local energetic hierarchy of idealised tilt distortions, without imposing long-range molecular order. They therefore clarify which aspects of the tilt landscape are weakly or strongly constrained locally, but do not by themselves determine the thermodynamically stable finite-temperature phase.

The bond-length-dependent potential energy surfaces in Fig. S16 show that the tilt energetics cannot be interpreted independently of local lattice relaxation. For all three A-site compositions, the minimum-energy path shifts systematically in the two-dimensional space of tilt angle and bond length, indicating strong coupling between octahedral rotation and framework bond length relaxation. A fixed-bond-length cut therefore does not provide a physically complete description of the tilting energetics. For comparison between different tilt modes, the tilt coordinate is defined through the norm of the three octahedral tilt components. This is particularly important for comparing $a^-a^-c^+$ and $a^+a^+a^+$ distortions, because molecular-dynamics trajectories show that in the $a^-a^-c^+$ mode the typical relative amplitudes of the two equivalent in-plane tilts and the out-of-plane tilt are approximately $1:2$. Accordingly, for a variable angle parameter $\theta$, the $a^-a^-c^+$ mode is constructed as $[\theta/\sqrt{2}, \theta/\sqrt{2}, \sqrt{2}\theta]$, whereas the $a^+a^+a^+$ mode is written as $[\theta, \theta, \theta]$. With this choice, equal values of the variable angle correspond to equal norms of the three-component tilt vector, allowing the two modes to be compared consistently. From this perspective, $CsPbI_3$ shows the

clearest energetic preference for finite tilting, with minima that are lower than the cubic limit by up to $\sim 5$ meV/atom depending on the mode. It is noteworthy that the displacement of the Cs atom in the structure will favour the $a^-a^-c^+$ tilt mode over the single-tilt modes[8]. $MAPbI_3$ is intermediate, with only a small energetic stabilisation of tilted structures, typically below 2 meV/atom. By contrast, $FAPbI_3$ shows essentially no energetic preference for finite tilting at the local level: the lowest-energy configuration remains at, or extremely close to, the zero-tilt limit, and the $\Delta E < 10$ meV/atom region spans a broad range of tilt angles, extending from approximately $0°$ to $6°$ for $a^-a^-c^+$ and $a^+a^+a^+$ modes and to nearly $10°$ for $a^0a^0c^-$ and $a^0a^0c^+$. This broad, shallow basin reinforces the conclusion that in $FAPbI_3$ the local potential energy surface alone only weakly constrains the tilt amplitude and does not uniquely select the final correlation pattern or tilting magnitude.

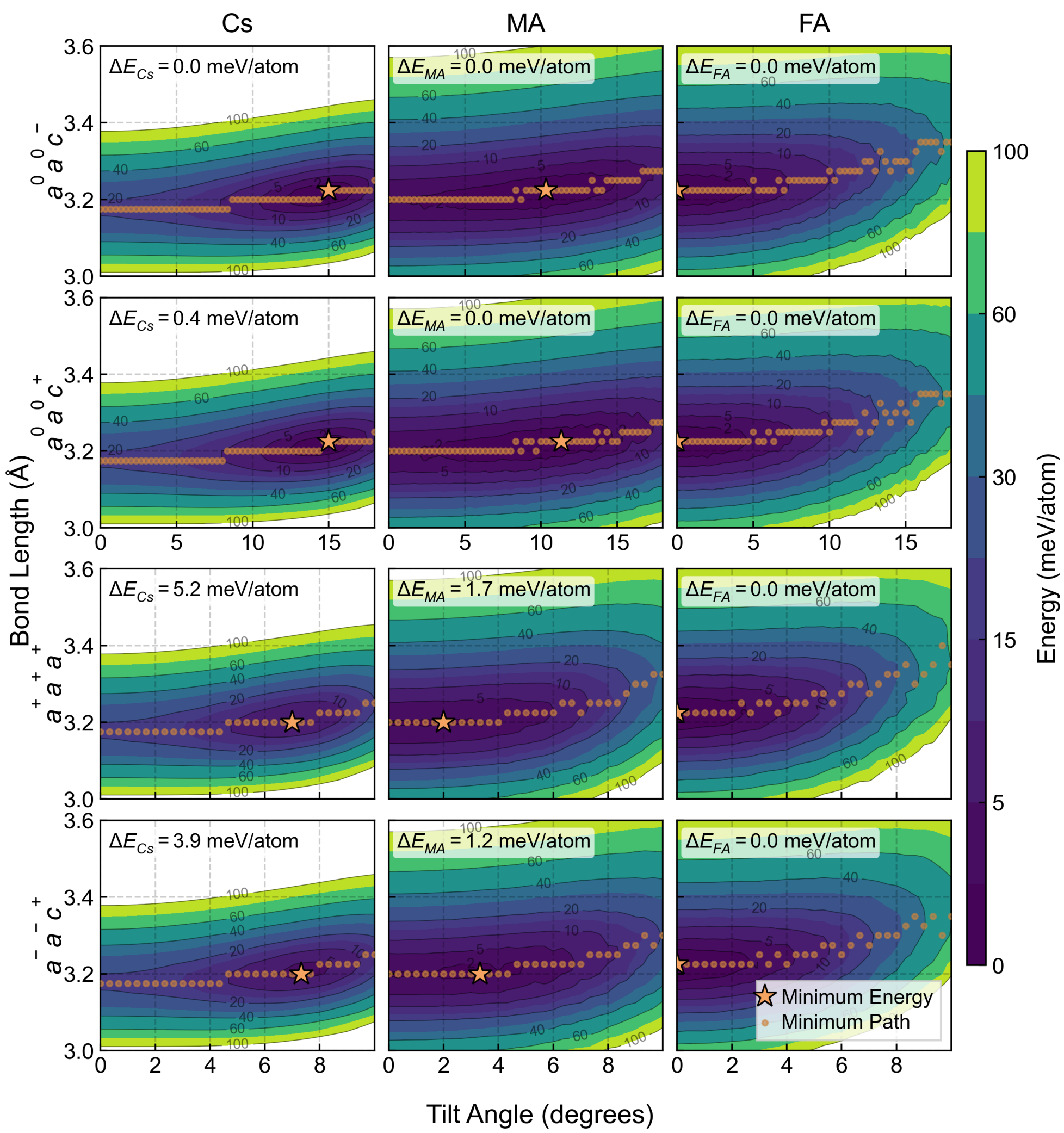


Figure S16: Two-dimensional potential energy surfaces of $CsPbI_3$, $MAPbI_3$, and $FAPbI_3$ as functions of tilt angle and bond length for the idealised modes $a^0a^0c^-$, $a^-a^-c^+$, $a^+a^+a^+$, and $a^0a^0c^+$. The plotted surfaces are averaged over 50 independent samples. Stars mark the global minimum on each surface, and circles trace the minimum-energy path with respect to tilt angle, illustrating the coupling between octahedral tilting and bond length relaxation.

## 6 Simulated Phase Transition Temperature

The $\alpha$-to-$\gamma$ transition temperature in our simulations (∼200 K) falls approximately 80 K below the experimental value (∼280 K). We attribute this to the following concurring contributions.

The MLFF is trained on $r^2$SCAN energies, forces, and stresses. While $r^2$SCAN represents a significant improvement over PBE for structural and thermodynamic properties[9], semi-local functionals generically underestimate the free energy barrier between competing structural phases in anharmonic systems because they underestimate the curvature of the potential energy surface near the high-symmetry reference structure. This bias is directly inherited by the MLFF through its training data and depresses the computed transition temperature. Indirect evidence for this in halide perovskites is provided by the observation that MLFF-based simulations trained on comparable DFT data consistently yield transition temperatures below experiment[3,6,10].

A further source of transition temperature depression is the systematic softening of the DFT potential energy surface that occurs during MLFF training, arising from the finite density of training configurations and the implicit regularisation of the model. This effect has been identified across multiple neural-network-potential architectures and leads to an underestimation of effective energy barriers and, consequently, of the temperature at which ordered phases stabilise[11,12]. The present authors have verified this behaviour independently for Allegro[13] and GAP-based[14] potentials applied to halide perovskite systems, consistent with the broader evidence that this is an architecture-independent limitation of current MLFF training strategies.

# 7 Molecular Orientations

To characterise the orientational distribution of FA, each molecular vector is projected onto spherical polar coordinates $(\phi, \theta)$ and accumulated into a two-dimensional hexagonal histogram. Denoting the normalised population of bin $k$ by

$$p_k = \frac{n_k}{\sum_j n_j}, \tag{8}$$

we quantify the degree of orientational order through the variance of the normalised bin counts,

$$\sigma_p^2 = \frac{1}{N_{\mathrm{bin}}} \sum_{k=1}^{N_{\mathrm{bin}}} (p_k - \bar{p})^2, \qquad \bar{p} = \frac{1}{N_{\mathrm{bin}}} \sum_{k=1}^{N_{\mathrm{bin}}} p_k. \tag{9}$$

A nearly uniform orientational distribution gives a small variance, whereas localisation into preferred directions increases $\sigma_p^2$. In this way, the variance of the normalised hexbin counts provides a scalar order parameter complementary to the explicit pair-correlation analysis in the main text.

To analyse orientational correlations beyond the polar-angle distributions, each FA cation is represented by two molecular vectors, $v_{\mathrm{FA1}}$ and $v_{\mathrm{FA2}}$, corresponding to the two orthogonal molecular axes used in the main-text pair-correlation analysis. Here $v_{\mathrm{FA1}}$ is defined as the vector from the midpoint of the two N atoms to the C atom, while $v_{\mathrm{FA2}}$ connects the two N atoms. To verify that the $\langle 100 \rangle$-based orientational analysis is not imposed by construction, we generated 100 independent $FAPbI_3$ configurations by randomising all FA orientations in the equilibrium cell and then relaxing only the molecular degrees of freedom with the trained MLFF while keeping the inorganic framework fixed ($a^0a^0a^0$ mode). The energies before and after FA-only relaxation both separate into two clear groups (Fig. S17a), indicating distinct molecular arrangements within the same fixed framework. More importantly, after relaxation both $v_{\mathrm{FA1}}$ and $v_{\mathrm{FA2}}$

are strongly concentrated near the symmetry-folded $\langle 100\rangle$ direction (Fig. S17b). This shows that even when initialised from fully random orientations, the FA molecules relax back into the same preferred orientational basin. The (100)-restricted initialisation used in the production MD therefore does not impose an artificial ordering pattern, but reflects the orientational manifold selected naturally by the dynamics.

Fig. S17c,d further shows the polar-projection distributions of the FA1 and FA2 molecular vectors as a function of temperature, together with the corresponding orientational-order measure. The highest-density regions remain centred on the $\langle 100\rangle$ directions, while the distribution broadens substantially on heating. The temperature dependence of the variance confirms that orientational localisation becomes strongest near the low-temperature ordered regime and weakens again towards the high-temperature dynamically disordered phase.

For reference, the random benchmark used in Fig. 3b was constructed from ten independently generated configurations with completely random FA molecular orientations. The nearest-neighbour FA-pair statistics were evaluated for each configuration using the same analysis protocol as for the MD trajectories, and the resulting occupancies were averaged to obtain the reference distribution. This benchmark therefore represents the orientational statistics expected in the absence of intermolecular orientational correlations. Relative to this benchmark, the ordered $\gamma$ phase develops a symmetry-selected anisotropic molecular-correlation pattern: for a pair separated along one axis $\alpha$, FA1–FA1 favours transverse $\pm\beta_1$ relations, while FA2–FA2 favours transverse $\pm\beta_2$ relations, where $\alpha$, $\beta_1$, and $\beta_2$ denote the three principal directions as illustrated in Fig. 3a. This directional pattern is commensurate with long-range $a^+a^+a^+$ tilt coherence and is the molecular-ordering topology that fails to develop globally in the low-temperature $\gamma'$ state.

The molecular reorientation time is extracted from the temporal autocorrelation of

the molecular orientation vectors, as in the PDynA formalism,

$$A_{\mathrm{MO}}(t) = \langle \mathbf{v}(t_0; \mathbf{n}) \cdot \mathbf{v}(t_0 + t; \mathbf{n}) \rangle_{t_0, \mathbf{n}} \,, \tag{10}$$

which is fitted to the same biexponential form of Eq. 7. Following the original implementation, $\tau_1$ is taken as the characteristic reorientation time because it dominates the decay, while $\tau_2$ accounts for the fast initial correction term.

Assuming activated rotational dynamics, the temperature dependence of the fitted reorientation time is described by

$$\tau_r(T) = \tau_0 \exp\left(\frac{E_\mathrm{a}}{k_\mathrm{B} T}\right), \tag{11}$$

or equivalently

$$\ln \tau_r = \ln \tau_0 + \frac{E_\mathrm{a}}{k_\mathrm{B}} \frac{1}{T}. \tag{12}$$

A linear fit to $\ln \tau_r$ versus $1/T$ thus yields the effective activation energy $E_\mathrm{a}$ from the slope. We emphasise that the resulting $E_\mathrm{a}$ should be interpreted as a coarse-grained kinetic barrier associated with molecular reorientation in the fluctuating framework environment, rather than as a unique microscopic rotation barrier, because the motion is coupled to local lattice distortions and intermolecular correlations. In practice, the fit is restricted to the temperature window where approximately Arrhenius behaviour is observed. The resulting barriers obtained here are consistent with previously reported values. For example, quasielastic neutron-scattering and spectroscopic studies have reported activation energies of order $\sim$ 20–30 meV for dipole reorientation of FA molecules around their principal axis in hybrid perovskites, while larger barriers of order 40–100 meV have been associated with more complete molecular rotations involving stronger coupling to the inorganic framework[15,16]. The values extracted in the present work therefore

fall within the range expected for thermally activated molecular reorientation in these materials. Additionally, the simulated values suggest that, at the same temperature, the rotation of molecules is favoured around the FA1 axis as well as in the twinned structure, which is consistent with the intuition from structural geometry.

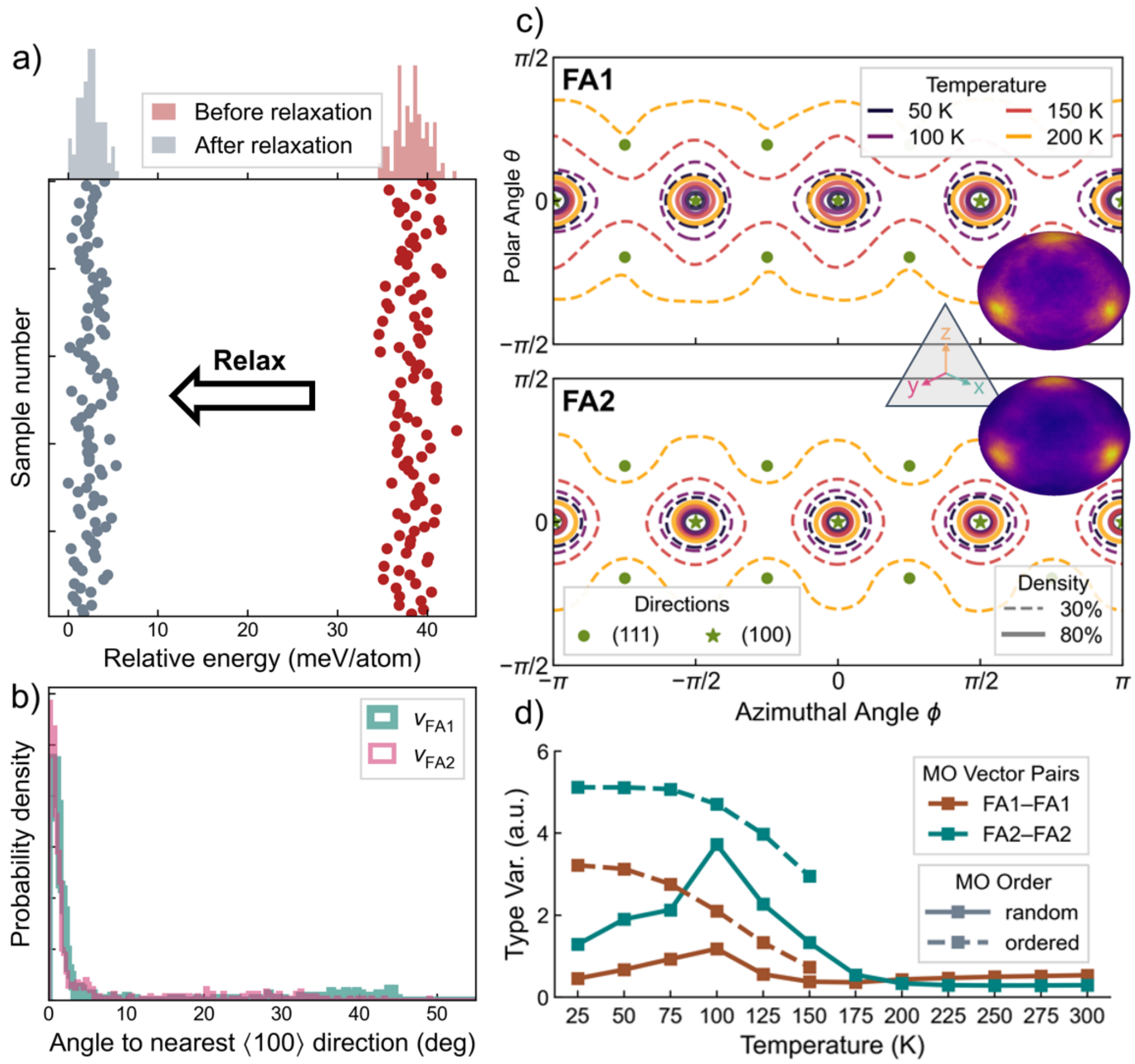


Figure S17: FA molecular orientations in $FAPbI_3$. (a) Relative energies of 100 structures with randomly initialised FA orientations before and after FA-only relaxation, referenced to the lowest relaxed configuration. The top histogram shows the corresponding energy distributions. (b) Distribution of the angle between each relaxed molecular vector and the nearest ⟨100⟩ direction, obtained by taking the maximum dot product with the six Cartesian ⟨100⟩ directions. (c) Polar-projection visualisations of the FA1 and FA2 molecular orientation distributions at different temperatures, with density contours highlighting the preferred (100)-type directions. The inset spheres illustrate the corresponding 3D distributions. (d), Temperature dependence of the orientational-order measure defined as the variance of the normalised hexbin counts; larger values indicate stronger localisation of the molecular orientations.

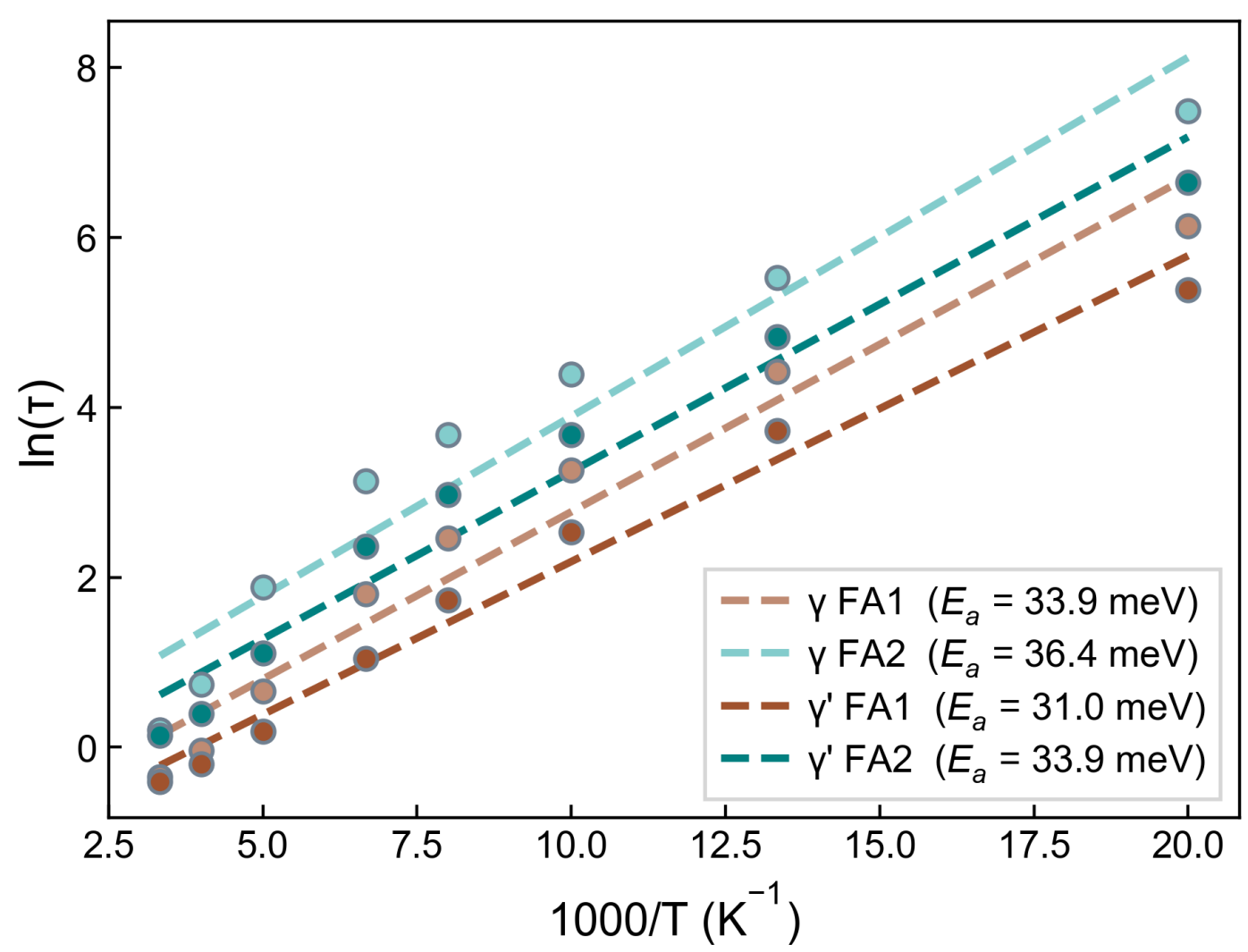


Figure S18: Arrhenius analysis of the FA reorientation times. Characteristic reorientation times extracted from the molecular-orientation autocorrelation functions are plotted as $\ln \tau_r$ versus $1000/T$, and the slope of the linear fit yields the effective activation energy $E_\mathrm{a}$. Separate fits are shown for FA1 and FA2 in the ordered $\gamma$ and twinned $\gamma'$ states.

# 8 Artificial Planar Disorder Domain and Energetic Competition

Unless otherwise stated, the ordered $\gamma$ and heterogeneous $\gamma'$ structures used in the main text analyses were not imposed by hand, but selected from the large ensemble of cooling and equilibration MD trajectories described in the Methods. These trajectories generate many representative low-temperature configurations depending on temperature history. Structures labelled as ordered $\gamma$ were taken from trajectories that developed long-range $a^+a^+a^+$ coherence along all three crystallographic directions, whereas structures labelled as $\gamma'$ were taken from trajectories that remained locally $\gamma$-like but developed reduced long-range coherence and heterogeneous low-temperature textures.

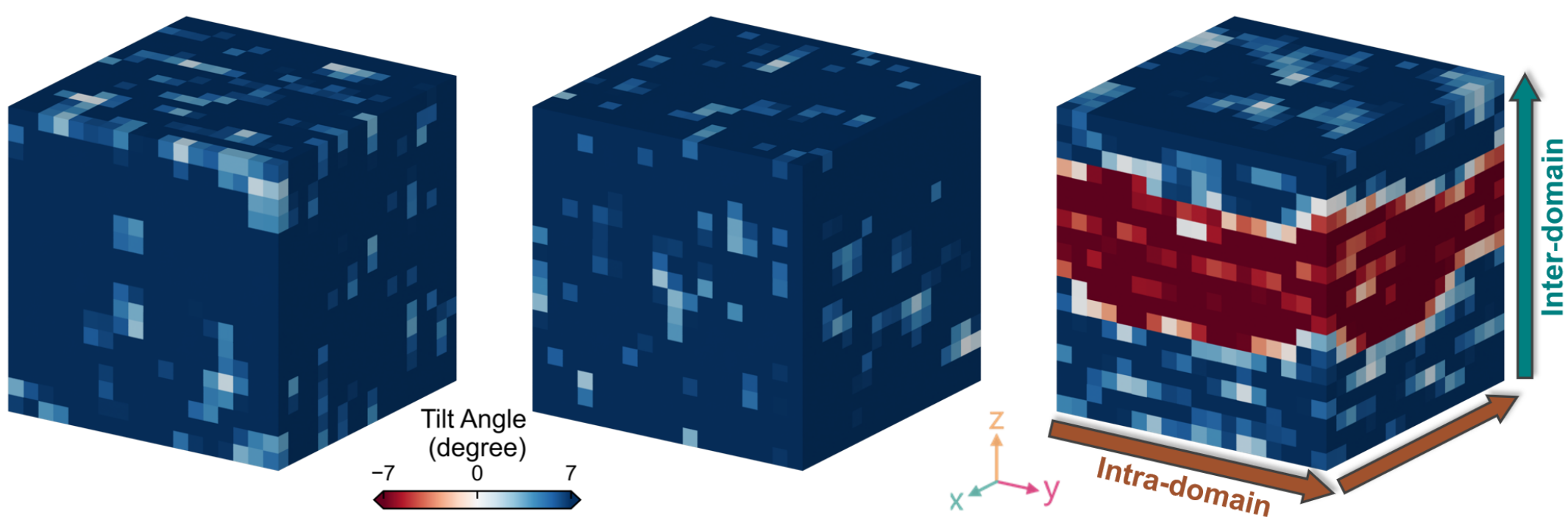


Figure S19: Three-dimensional tilt map of an instantaneous frame of $FAPbI_3$ at 100 K containing an artificial planar twin boundary perpendicular to $z$. Tilt coherence is preserved within the domain planes but interrupted across the boundary, providing a control structure for identifying the reciprocal-space signature of a single twin interface.

By contrast, an artificial structure was constructed to isolate the scattering signature of a single twin boundary. Starting from a perfect $a^+a^+a^+$ supercell, half of the octahedra in a central slab were shifted by one pseudocubic unit cell along the $y$ direction, thereby creating a planar discontinuity in the tilt pattern. This operation preserves coherent $x$- and $y$-directed tilts within the plane while introducing a twinned $z$-tilt across the slip planes. The structure was subsequently equilibrated at 100 K, where it remains

metastable on the simulation timescale and can therefore be used as a clean control for the reciprocal-space signature of a single boundary. In Fig. S19, the intra-domain directions (parallel to the boundary plane) and the inter-domain direction (perpendicular to the boundary) are explicitly illustrated, which provides a visual reference for the directional anisotropy of the resulting scattering features discussed in the main text (Fig. 4).

Because the discontinuity is encountered only when crossing the boundary, the reciprocal-space response is strongly anisotropic. The $HK$ half-integer plane remains essentially identical to the ordered bulk $\gamma$ phase, whereas the $HL$ and $KL$ planes become sensitive to the boundary and develop the satellite-like diffuse features discussed in the main text.

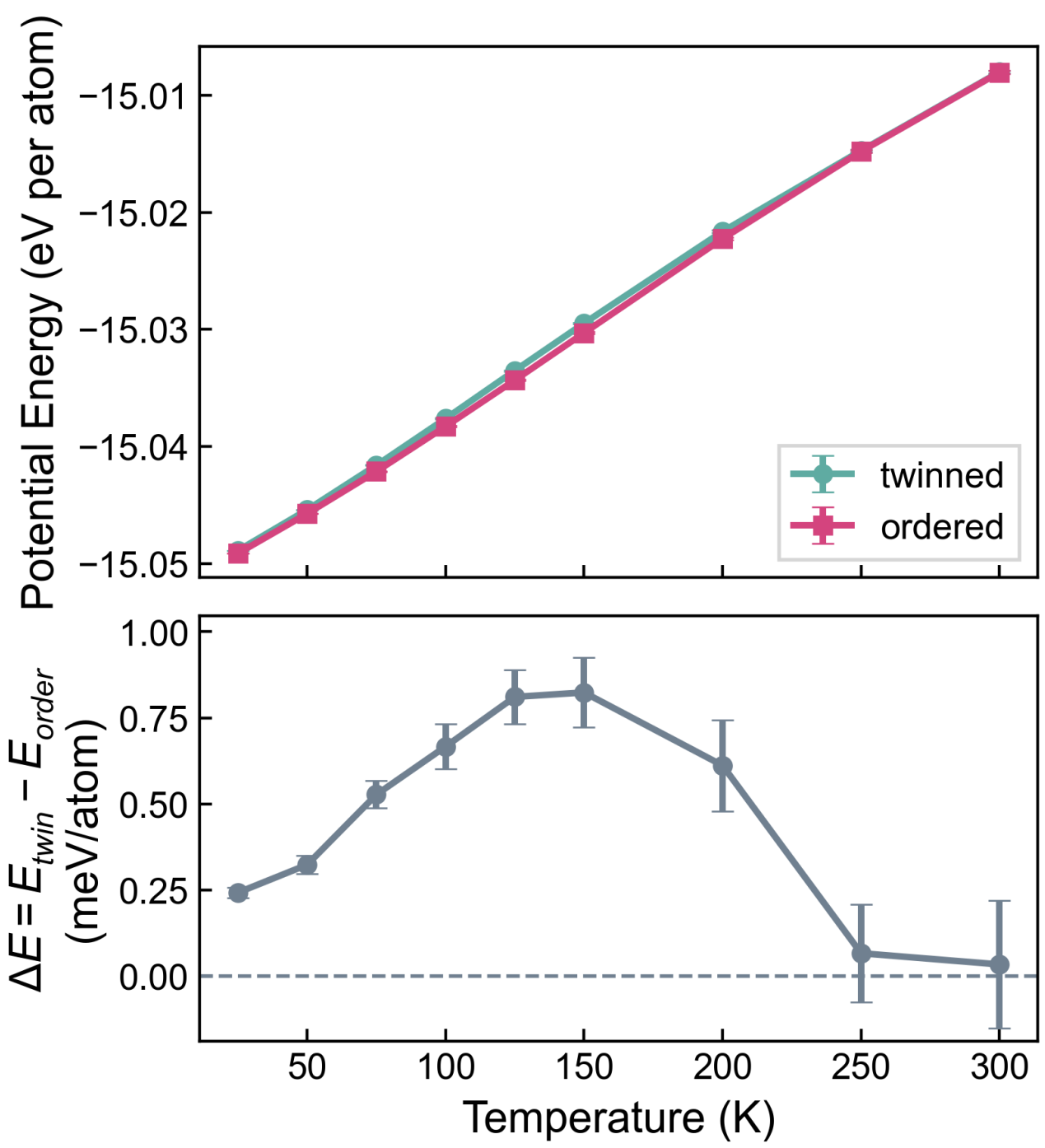


Figure S20: Potential energy per atom of the ordered $\gamma$ and twinned $\gamma'$ states as a function of temperature. Top panel: absolute potential energies. Bottom panel: energy difference $\Delta E = E_{\text{twin}} - E_{\text{order}}$. The ordered $\gamma$ state is always the energetic minimum, whereas the twinned $\gamma'$ state becomes nearly degenerate at high temperature, consistent with kinetic arrest rather than energetic stabilisation.

The energetic competition between the ordered $\gamma$ phase and the twinned $\gamma'$ state is summarised in Fig. S20. The ordered $\gamma$ structure is always lower in potential energy, confirming that it is the local energetic minimum. However, the energy difference is largest at $\gamma$ phase temperature and decreases strongly on heating, approaching zero within uncertainty at high temperature. This behaviour supports the interpretation advanced in the main text: the $\gamma'$ state is not the equilibrium minimum but a kinetically arrested metastable state, stabilised dynamically by the freezing of the molecular and tilt rearrangements rather than by a lower static energy[17]. The near-degeneracy at high temperature also explains why multiple low-temperature topologies can become accessible depending on the thermal history.

## 9 Space Group Determination of the $\gamma$ Phase

The low-temperature $\gamma$ phase of $FAPbI_3$ has been assigned in the literature to multiple tilt systems, including the lower-symmetry tetragonal pattern $a^0a^0c^+$. Here we confirm experimentally that the $\gamma$ phase is best described by the $a^+a^+a^+$ tilt pattern (space group Im$\bar{3}$) by comparing the measured X-ray scattering with structure factors calculated for candidate tilt systems.

Fig. S21a shows the experimental X-ray scattering pattern on the $HK1.5$ reciprocal-space plane measured at 150 K. This plane was chosen because it contains specific superstructure reflections, highlighted by white circles, that are uniquely allowed by the $a^+a^+a^+$ tilt pattern and forbidden in lower-symmetry tilt systems. The corresponding pattern obtained from our large-scale MD trajectories, which were independently identified as $a^+a^+a^+$ from the real-space tilt analysis, reproduces these characteristic superstructure reflections (Fig. S21b).

To further isolate the structural origin of these reflections, we computed structure factors directly from idealised CIF models for two candidate tilt patterns. For the $a^+a^+a^+$ model, Cs was placed on the A-site as a computationally convenient proxy, since the FA molecule contributes only weakly to the structure factor relative to Pb and I. The Cs substitution therefore introduces only a small perturbation to the diagnostic superstructure intensities. The resulting $a^+a^+a^+$ pattern (Fig. S21c) clearly reproduces the reflections highlighted in the experimental data.

By contrast, the $a^0a^0c^+$ model, which has previously been used to describe related FA lead-halide phases, does not generate these reflections even when three symmetry-equivalent twin orientations are superimposed to mimic the twinning scenario encountered in $MAPbI_3$ (Fig. S21d). The diagnostic superstructure peaks are forbidden in each individual twin and therefore remain absent in their superposition.

Taken together, the experimental observation of these reflections, their reproduction by MD-based diffraction simulations, and their absence in the twinned $a^0a^0c^+$ model provide a consistent and specific structural fingerprint that identifies the $\gamma$ phase of $FAPbI_3$ as $a^+a^+a^+$.

The temperature dependence of the experimental scattering on this plane further supports the $a^+a^+a^+$ assignment (Fig. S22). At 130 K and 150 K, the superstructure reflections are clearly visible at the half-integer positions highlighted in Fig. S21a, consistent with long-range $a^+a^+a^+$ tilt coherence within the measured volume. At 280 K and 295 K, these superstructure reflections are no longer observed, indicating that the static in-phase tilt order characteristic of the $\gamma$ phase is absent. Instead, the higher-temperature patterns retain pronounced diffuse scattering around the Bragg positions and near the zone boundary, reflecting the dynamic local tilt correlations of the $\alpha$-like regime rather than a frozen long-range tilt pattern.

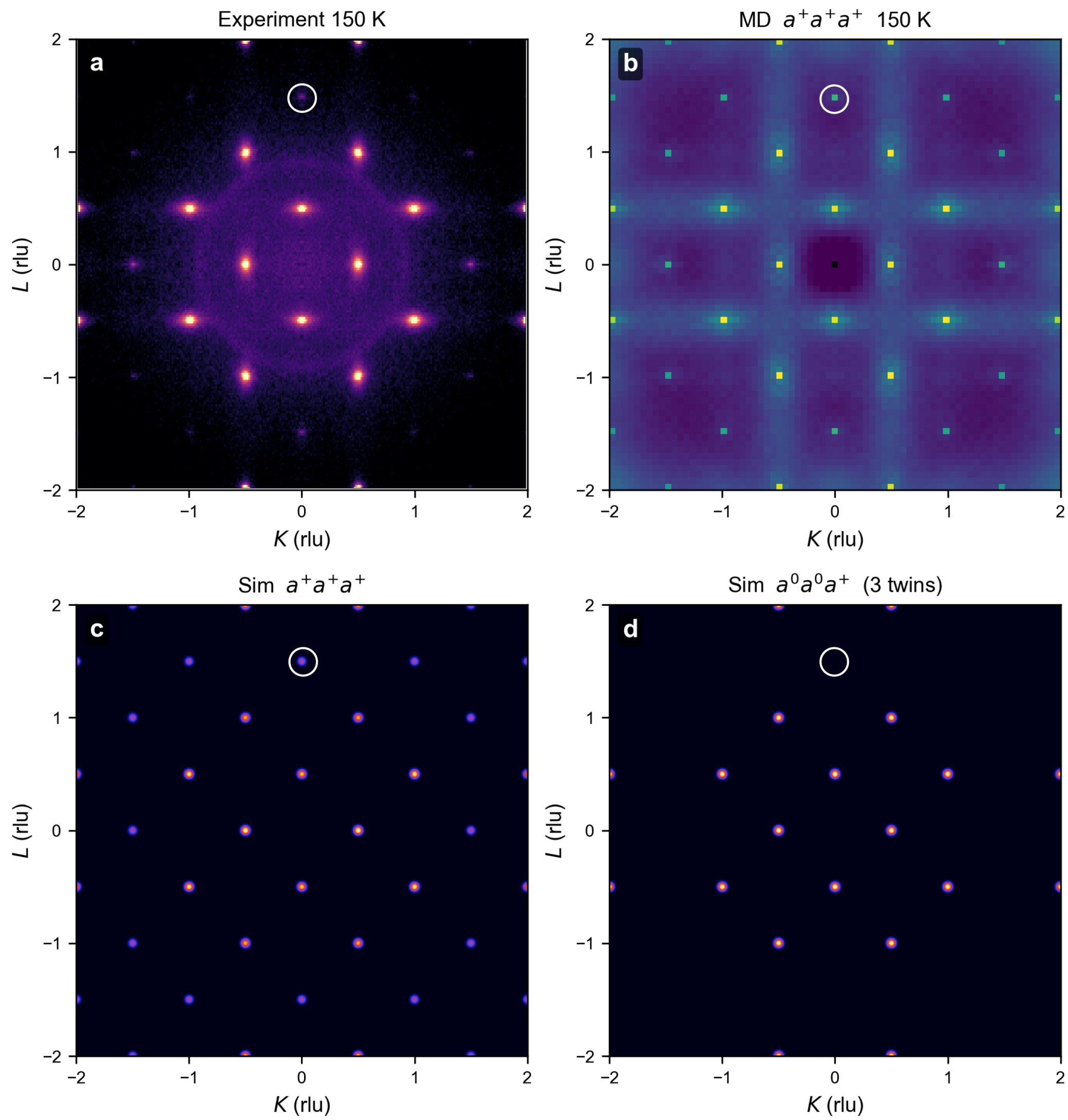


Figure S21: Space group determination of the $\gamma$ phase of $FAPbI_3$. (a) Experimental X-ray scattering pattern on the $HK1.5$ reciprocal-space plane at 150 K. White circles highlight superstructure reflections that are uniquely allowed by the $a^+a^+a^+$ tilt pattern. (b) Corresponding diffraction pattern computed from MD trajectories independently identified as $a^+a^+a^+$ in real space, reproducing the same superstructure reflections. (c) Structure factor simulation from CIF model with $a^+a^+a^+$ symmetry. (d) Structure factor simulation from an $a^0a^0c^+$ model with three symmetry-equivalent twin orientations superimposed. All diffraction patterns show $HK1.5$ reciprocal-space planes. The diffraction ring in (a) originates from ice formation on the crystal during the measurement.

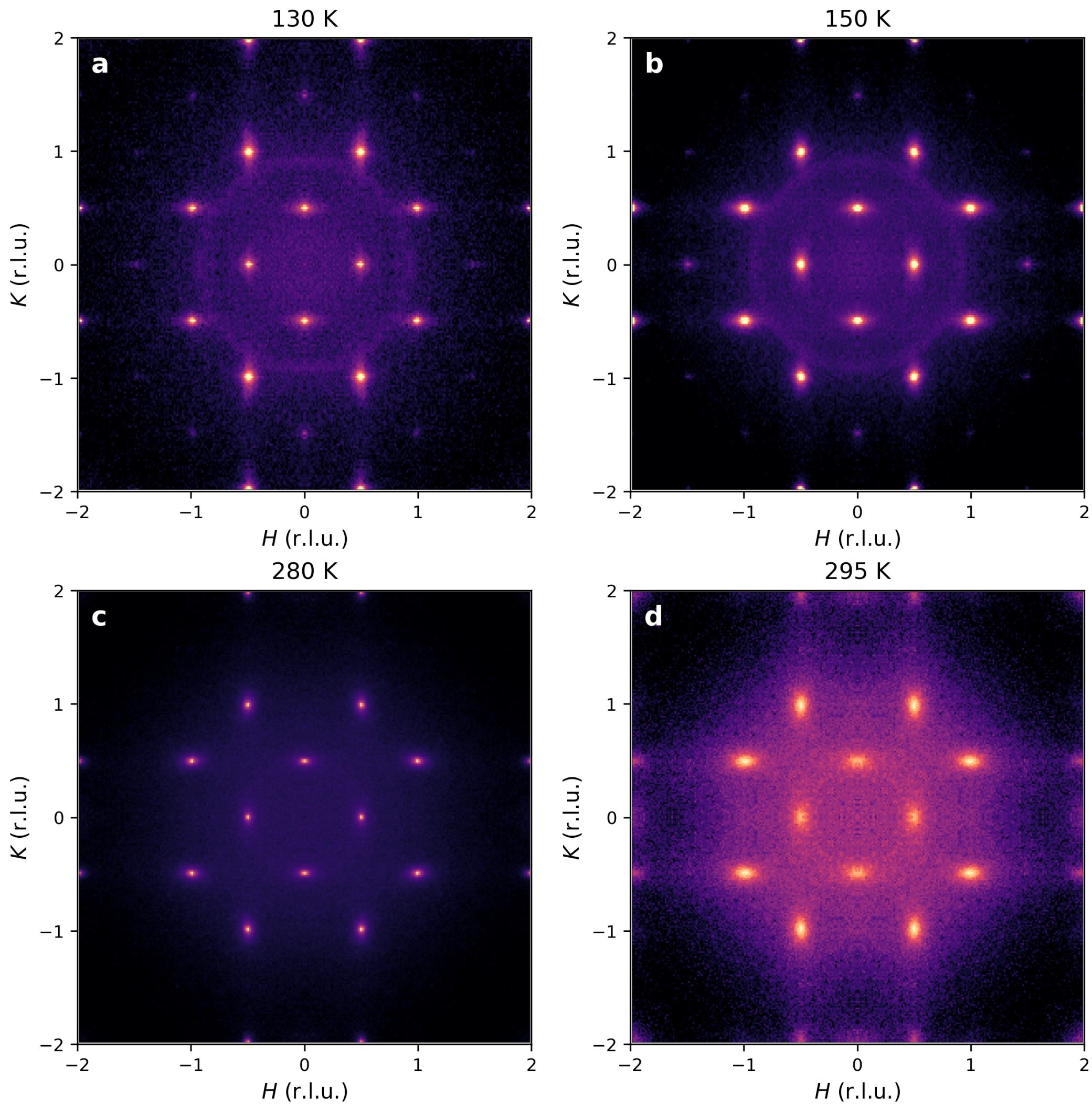


Figure S22: Temperature dependence of the experimental X-ray scattering of $FAPbI_3$ on the $HK1.5$ reciprocal-space plane. (a) 130 K and (b) 150 K, in the $\gamma$ phase, where the characteristic $a^+a^+a^+$ superstructure reflections are present at the half-integer positions highlighted in Fig. S21a. (c) 280 K and (d) 295 K, above the ordering transition, where these superstructure reflections are no longer present and the pattern is instead dominated by diffuse scattering around the Bragg positions, characteristic of the dynamically disordered $\alpha$ phase.

## 10 Diffuse Scattering and Reciprocal-Space Analysis

The reciprocal-space observables in this work were computed from MD trajectories using the previously established real-space-to-scattering workflow for perovskites[18], which was subsequently tested successfully in related hybrid lead-halide systems[19]. In this approach, the instantaneous atomic configurations from MD are converted into reciprocal-space intensities by evaluating the corresponding structure-factor expressions over the sampled trajectory, yielding both energy-integrated diffuse scattering and the dynamical structure factor $S(\mathbf{q}, \omega)$. The full formalism is described in those earlier works; here we use the same workflow and focus on the specific reciprocal-space sampling, averaging, and correlation length extraction procedures adopted for $FAPbI_3$.

For the energy-resolved reciprocal-space maps, the raw simulated $S(\mathbf{q}, \omega)$ was first evaluated on a regular reciprocal-space grid spanning 0–5 r.l.u. along each reciprocal direction. Each sampled $\mathbf{q}$ point was then folded into the first Brillouin zone by reducing its coordinates modulo 1, and intensities contributing to the same reduced reciprocal-space bin were averaged to obtain a folded spectrum on a regular reduced-zone grid. For cubic structures, an additional averaging was performed over bins related by permutation of the $x$, $y$, and $z$ reciprocal axes. The folded spectrum was then sampled along the reduced-zone high-symmetry path $\Gamma \to M \to R \to \Gamma \to X \to M$, sorted along the energy axis, normalised by its maximum intensity, and plotted as the folded dynamical structure factor.

To compare equivalent diffuse features around the zone-boundary $M$ points, one-dimensional line profiles were extracted from the energy-integrated scattering intensity $S(\mathbf{q})$ on a cubic reciprocal-space grid. For the cubic case analysed here, we construct the 12 symmetry-equivalent line cuts

$$(H, 2.5, 1.5), \qquad (2.5, K, 1.5), \qquad (2.5, 1.5, L),$$

with $H, K, L \in \{0, 1, 2, 3\}$, corresponding to scans through all symmetry-equivalent $M$-point environments in the chosen half-integer planes. For each cut, the intensity is averaged over a small transverse window in the orthogonal directions, cropped to a window around the nominal $M$-point region, and normalised to unit peak height. Because the reciprocal-space grid spacing of the MD data is limited to 1/18 r.l.u., the individual line profiles are then linearly interpolated onto a common grid before averaging, which improves the numerical stability of the profile comparison and full width at half maximum (FWHM) extraction but does not remove the underlying resolution limit of the original data.

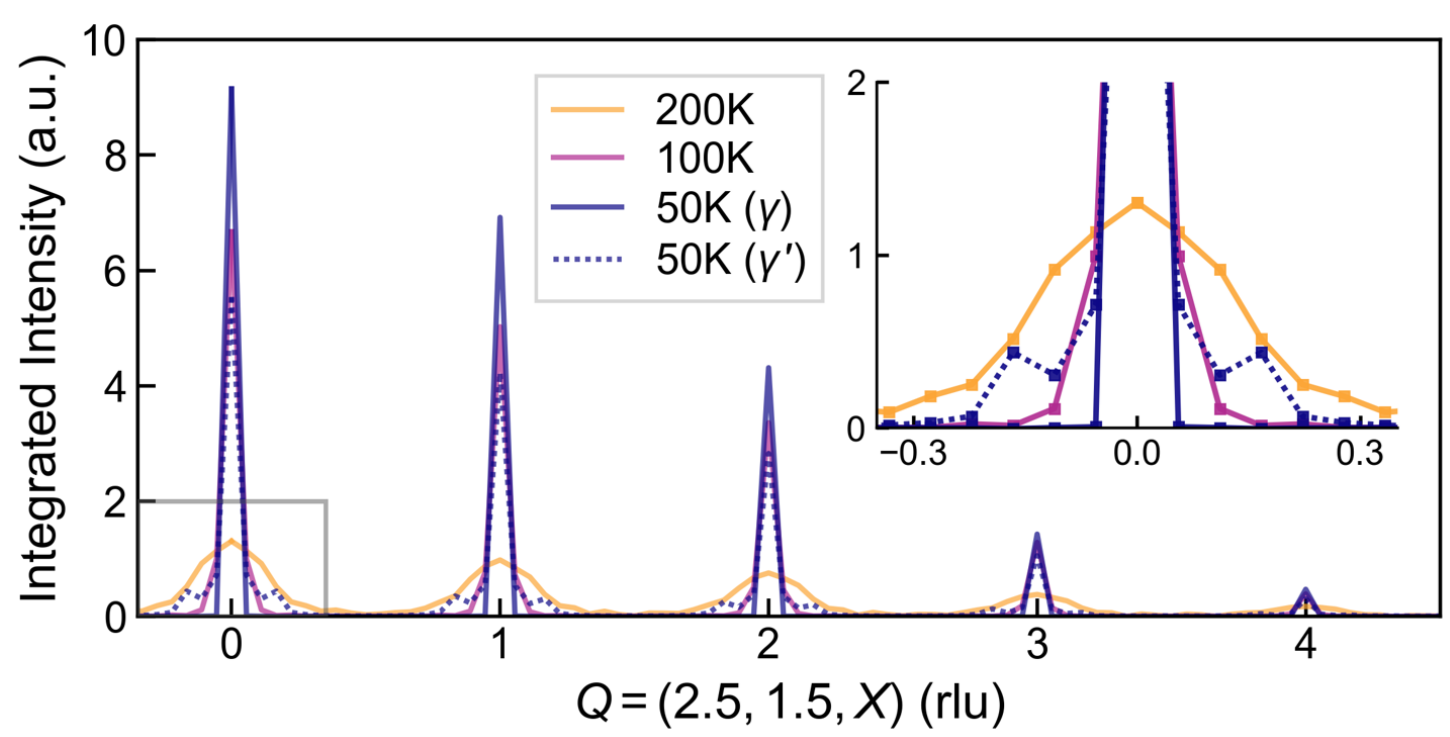


Figure S23: Energy-integrated X-ray scattering intensity $S(q)$ extracted along the reciprocal-space line profile $(2.5, 1.5, X)$. Ordered and disordered structures are shown with solid and dashed lines, respectively. The inset highlights the near-$M$ line shape, including the satellite-like intensity characteristic of the twinned $\gamma'$ state.

The reciprocal-space correlation length is extracted from the FWHM of this averaged profile, following the FWHM-based procedure used in the diffuse scattering analysis of related FA-based lead-halide perovskites[19]. If $q_L$ and $q_R$ denote the left and right half-maximum crossing points of the peak, then

$$\text{FWHM} = q_R - q_L, \tag{13}$$

and the associated reciprocal-space correlation length is estimated as

$$\xi_{\text{rec}} = \frac{2a_{\text{pc}}}{\pi\,\text{FWHM}}, \tag{14}$$

where $a_{\text{pc}}$ is the pseudocubic lattice constant. This expression corresponds to the standard Lorentzian/FWHM estimate used to connect reciprocal-space peak widths to a real-space correlation length.

Fig. S24 illustrates the fitting procedure. The experimental 293 K profile is well described by a single broadened peak, so the FWHM extraction is robust. In contrast, the MD 150 K ordered $\gamma$ profile is already so sharp that its width approaches the reciprocal-space grid spacing, making the fitted width effectively resolution-limited even after linear interpolation. For the low-temperature twinned $\gamma'$ state, the problem is more severe: the presence of satellite intensity means that the near-$M$ profile is no longer well described by a single peak at all. In such cases, the FWHM-based correlation length becomes intrinsically uncertain and should be interpreted only as a rough reciprocal-space metric rather than a rigorous single-domain correlation length. The reciprocal-space length obtained from the peak width is therefore an observable-dependent measure of diffuse scattering coherence rather than a second estimate of the real-space tilt correlation decay length. Numerical equality between these two quantities is neither expected nor required. The reciprocal-space values are used primarily for the direct experiment–simulation comparison, for which the measured and simulated profiles are analysed using the same peak width procedure.

For the twinned $\gamma'$ state, the near-$M$ line profiles are generally non-Lorentzian because the diffuse signal contains contributions from twin boundaries and configuration-dependent domain textures. We therefore do not assign a single reciprocal-space correlation length to the $\gamma'$ state using the same FWHM procedure. Instead, the

characteristic length scale of the $\gamma'$ state is discussed using the real-space tilt correlation analysis shown in Fig. 1a of the main text.

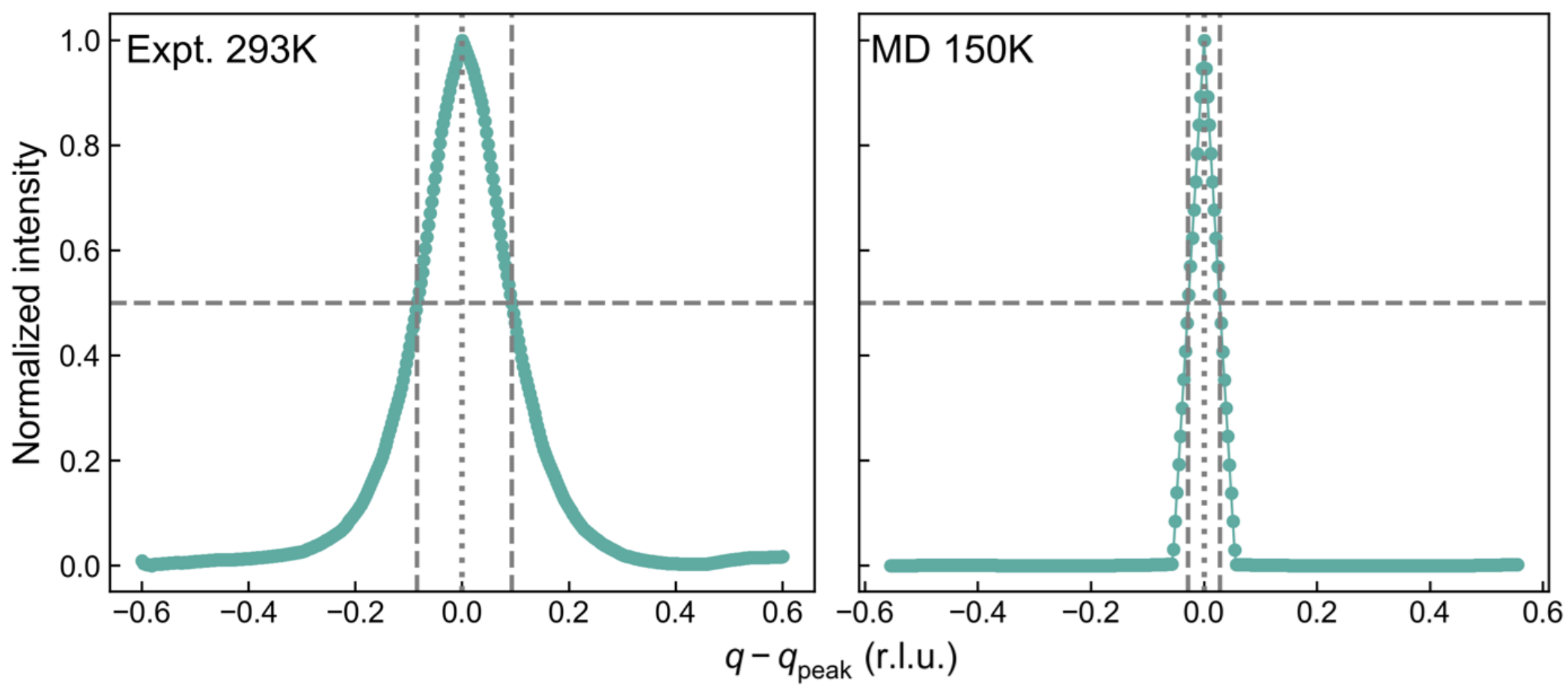


Figure S24: Examples of FWHM-based correlation length extraction from near-$M$ line profiles. Left: experimental 293 K data, for which the broadened single peak is well resolved. Right: MD 150 K data, where the peak width is close to the reciprocal-space resolution limit. Dashed vertical lines mark the half-maximum crossings.

To account for the strong configurational heterogeneity of the low-temperature twinned $\gamma'$ state, the diffuse patterns shown in Fig. S25 (and Fig. 4a) were averaged over 12 independent MD calculations. In practice, this average combines four independent trajectories and the three symmetry-equivalent half-integer planes $HK1.5$, $HL1.5$, and $KL1.5$. The independence of these calculations was established through randomisation of both the A-site molecular orientations and the initial velocities. In each initial configuration, the FA1 vector of every molecule was assigned randomly to one of the six allowed $\langle 100 \rangle$ directions, and the corresponding FA2 vector was then assigned randomly to one of the two remaining perpendicular $\langle 100 \rangle$ directions, ensuring the geometrical constraint that FA1 and FA2 remain orthogonal. On top of this orientational randomisation, distinct initial velocity seeds were used in the MD runs to avoid generating similar initial octahedral configurations. This averaging is particularly important for the twinned $\gamma'$ state, whose reciprocal-space response varies substantially

between independent realisations, whereas the bulk $\gamma$ and $\alpha$ phases show much less configurational variation and therefore do not require the same level of averaging.

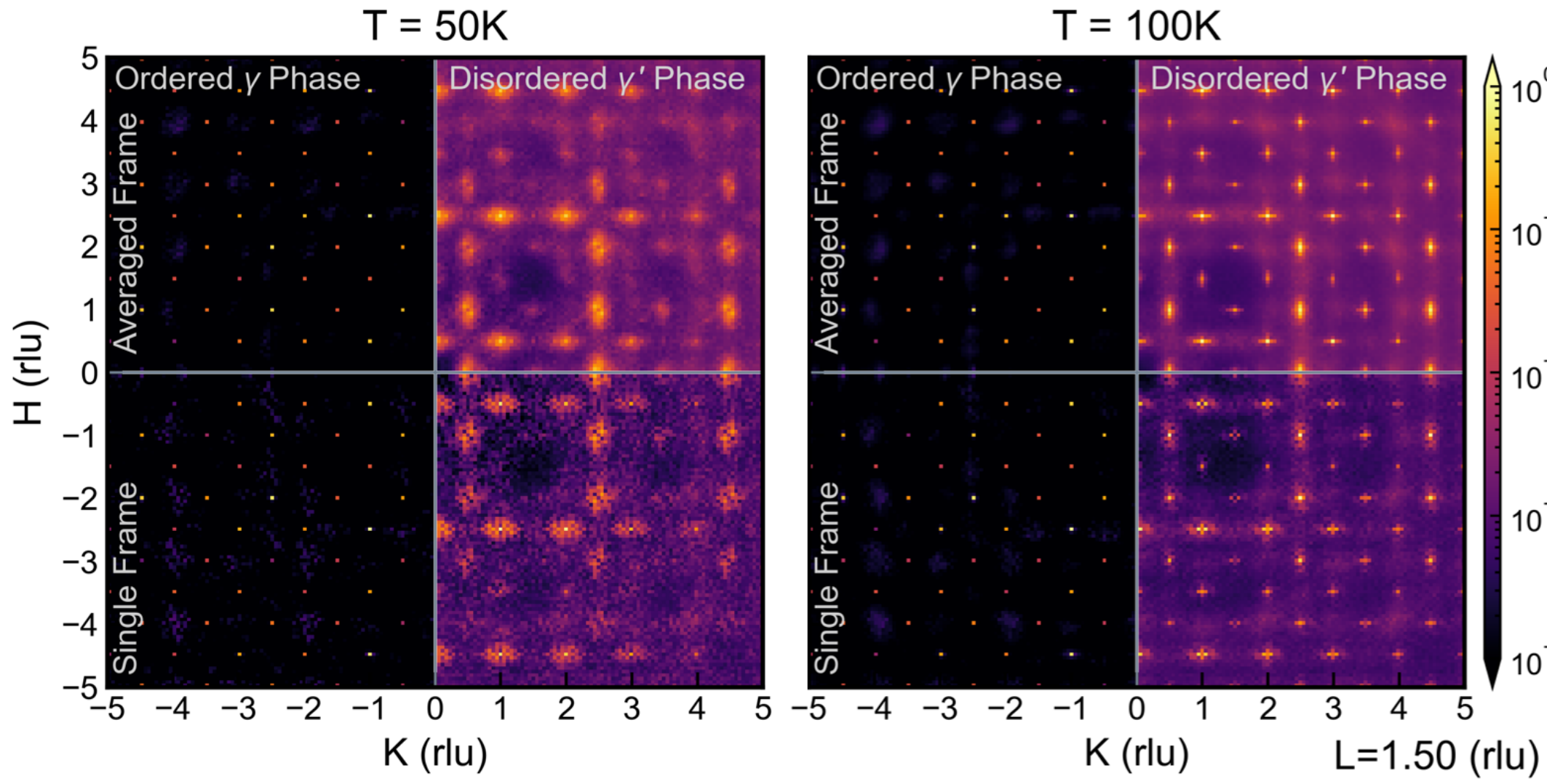


Figure S25: X-ray diffuse scattering of $FAPbI_3$ on the half-integer plane $L = 1.5$ r.l.u. at 50 and 100 K. The top row shows patterns averaged over four independent calculations and three symmetry-equivalent planes ($HK1.5$, $HL1.5$, and $KL1.5$); the bottom row shows one representative contribution from this 12-pattern average. The left and right columns correspond to the ordered $\gamma$ phase and the twinned $\gamma'$ state, respectively.

In addition to the energy-integrated diffuse scattering, the low-energy neutron response was also compared between structures initialised from ordered $\gamma$ and disordered $\gamma'$ states. The twinned $\gamma'$ configurations consistently retain broader low-energy spectral features than the ordered $\gamma$ phase, even at the same nominal temperature, whereas on heating both structures towards the high-temperature regime their responses become progressively more similar. This supports the interpretation that the broadening observed in the low-temperature $\gamma'$ state arises from arrested structural heterogeneity rather than from thermal disorder alone.

## 11 Electronic Disorder, Structural Featurisation, and Urbach Energy

Additional details supporting the electronic-disorder analysis are provided in the figures below. Fig. S26 shows an example of the Urbach-tail fitting procedure for the 50 K $\gamma'$ structure, illustrating the fitted low-energy tails near the valence and conduction band edges. In the main text, the Urbach energy is extracted from the statistical distribution of band-edge energies obtained from DFT calculations on subdivided $2 \times 2 \times 2$ subcells. For each subcell, the electronic band structure was computed, and the valence band maximum (VBM) and conduction band minimum (CBM) were identified. The sampled band-edge energies were then converted into normalised histogram-based density-of-states representations, and the low-energy tails were fitted to extract $E_U^{\mathrm{VBM}}$ and $E_U^{\mathrm{CBM}}$, from which the effective optical Urbach energy $E_U^{\mathrm{opt}}$ was obtained. Because these calculations were performed at the semilocal DFT level, the absolute gap values should be interpreted cautiously; the focus is on the relative variation of local band-edge energies across distinct structural environments rather than on quantitative prediction of the experimental bulk gap.

Because spin–orbit coupling strongly affects the Pb-derived conduction states in $FAPbI_3$, we performed an additional matched calculation to determine whether SOC changes the relative electronic-disorder trends obtained from the full non-SOC dataset.

Fig. S27 shows that inclusion of SOC lowers the $FAPbI_3$ band gap by approximately 1 eV across all three temperature subsets. After removal of the mean SOC-induced gap shift, the relative band gap ordering remains strongly preserved, with Pearson correlation coefficients of 0.954, 0.940, and 0.875 at 50, 100, and 200 K, respectively, and mean absolute deviations of 0.014–0.029 eV. The local environments identified as relatively low- or high-gap in the non-SOC treatment therefore remain similarly ordered

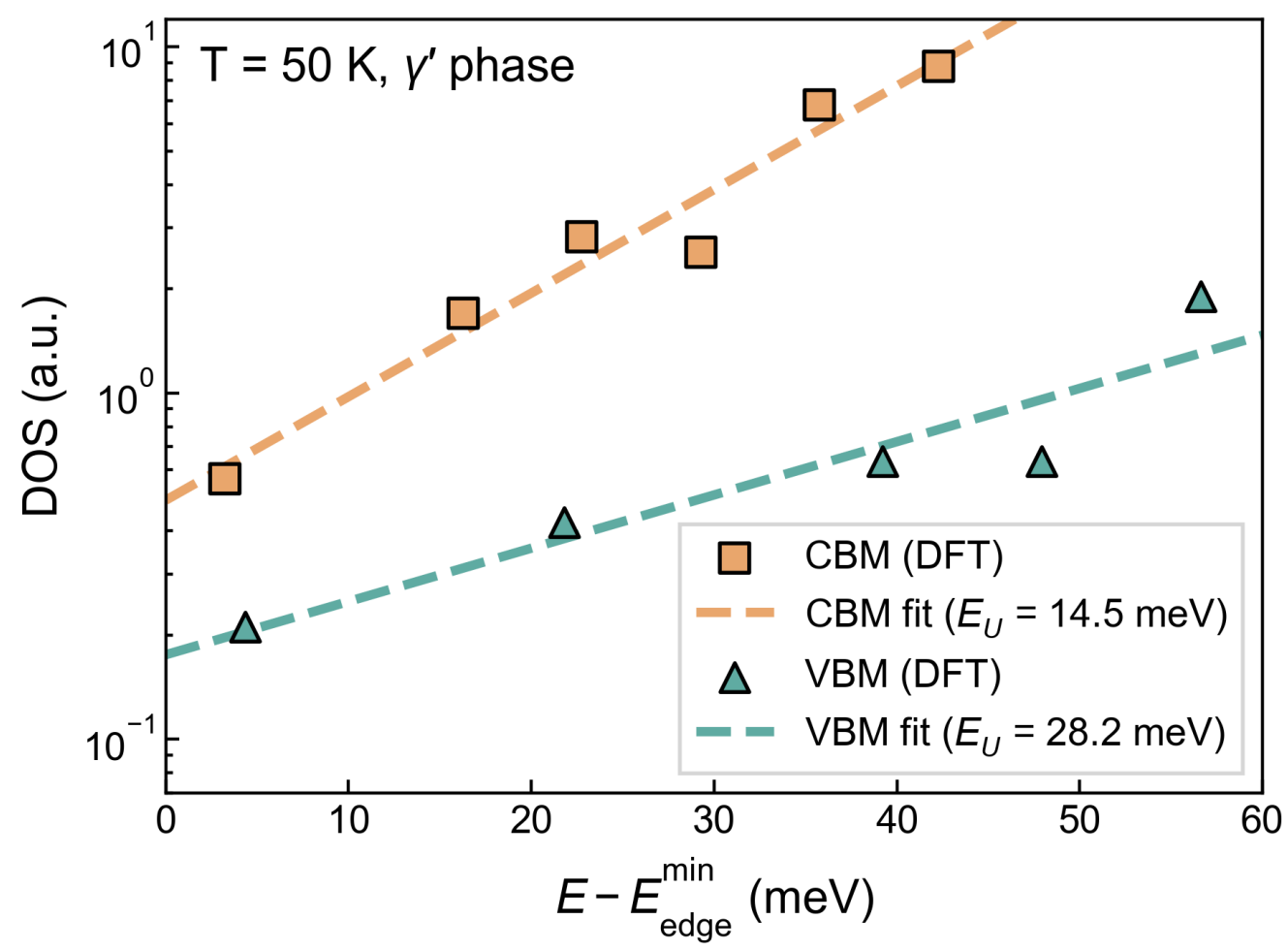


Figure S26: Example Urbach-tail fitting for the 50 K $\gamma'$ structure. Scatter points show the histogram-derived DOS near the valence and conduction band edges, and dashed lines show the exponential fits used to extract $E_U^{\mathrm{VBM}}$ and $E_U^{\mathrm{CBM}}$.

after SOC is included.

For comparison of the band-edge broadening, the VBM and CBM distributions were independently mean-centred before analysis. The fitted widths therefore quantify only the spread of each distribution and are invariant to an arbitrary additive energy reference. Because the SOC subsets contain 100 subcells at each temperature, Gaussian FWHM values were used as a stable measure of distribution width rather than fitting separate exponential tails. The mean-centred VBM and CBM distributions broaden with increasing temperature under both treatments. SOC transfers a larger fraction of this broadening to the conduction band edge, whereas the non-SOC treatment shows a larger valence-band contribution. Nevertheless, the overall widths remain on the same energy scale and retain the same temperature-dependent increase.

Because the electronic analysis is carried out on periodically repeated $2 \times 2 \times 2$ subcells extracted from the large MD supercell, it is useful to assess how this subcell treatment modifies the local structural distributions. Fig. S28 compares the distributions of octahedral distortion and tilt descriptors evaluated over the full supercell and

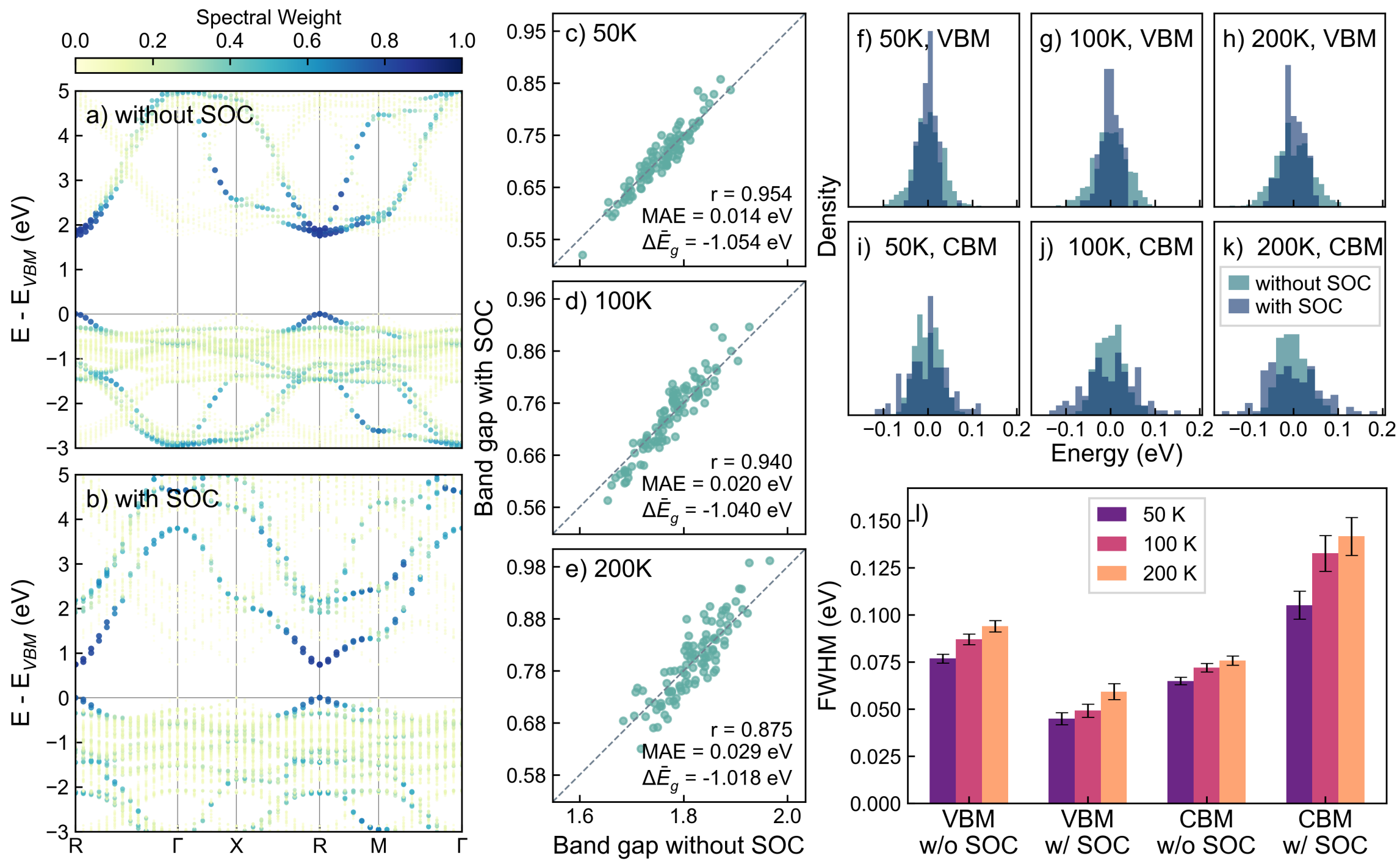

Figure S27: Sensitivity of the local electronic analysis to spin–orbit coupling. (a,b) Unfolded band structures calculated without and with SOC for the same $2 \times 2 \times 2$ subcell, with energies referenced to the corresponding valence-band maximum. SOC produces the expected reduction of the direct gap at the $R$ point while retaining the principal band-edge dispersion, consistent with previous calculations for $FAPbI_3$[20]. (c–e) Matched comparisons of band gaps calculated without and with SOC for randomly selected subcells at 50, 100, and 200 K. The mean SOC-induced gap shift is removed from the ordinate to expose the relative ordering of local environments; $r$ is the Pearson correlation coefficient, MAE is the mean absolute deviation from the shifted unit-slope relation, and $\Delta\bar{E}_g$ is the mean SOC-induced gap change. (f–k) Mean-centred VBM and CBM distributions calculated with and without SOC. (l) Gaussian FWHM values of the corresponding mean-centred distributions. Error bars show the standard errors propagated from the Gaussian-fitted distribution widths. SOC changes the partitioning of broadening between the valence and conduction edges while preserving its overall scale and temperature dependence.

over the corresponding extracted subcells. The tilting distribution is largely preserved by the subcell construction, indicating that the rotational environments relevant to the electronic analysis remain statistically representative at the population level. The imposed periodic boundary conditions nevertheless modify some local distortion com-

ponents, with the clearest shift occurring in the $T_{2g}$ mode and a smaller change in $T_{1u}$. We attribute these changes to the additional local constraints introduced when the extracted structures are periodically repeated, while the tilt distribution used as the principal electronic descriptor remains substantially more robust.

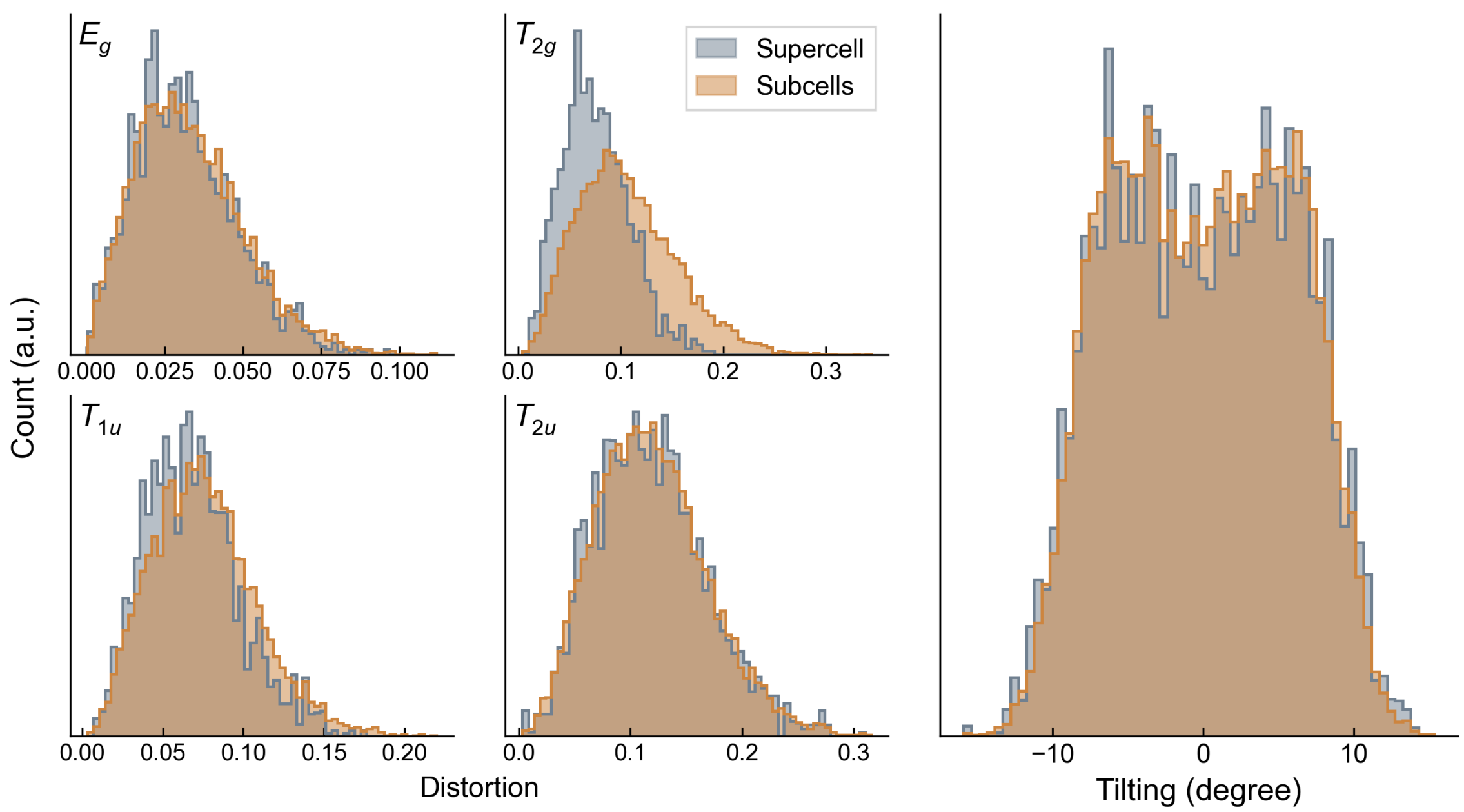


Figure S28: Comparison of structural descriptor distributions evaluated in the full MD supercell and in the extracted $2 \times 2 \times 2$ subcells used for the electronic analysis, from the 50 K $\gamma'$ structure. The right panel shows that the tilting distribution is largely unaffected by the subcell treatment. The left panels show that the octahedral distortion distributions are only modestly modified, with slight upward shifts most visible in the $T_{2g}$ and $T_{1u}$ modes, reflecting the periodic-boundary constraint imposed on the smaller cells.

To assess the dependence of the structure–electronic relationship on the spatial coarse-graining scale, the same parent MD configuration was additionally partitioned into $4 \times 4 \times 2$ subcells. The band gaps and octahedron-averaged tilt norms were then evaluated using the same electronic and structural analysis applied to the $2 \times 2 \times 2$ subdivision.

The comparison in Fig. S29 distinguishes the robustness of the tilt–gap relationship

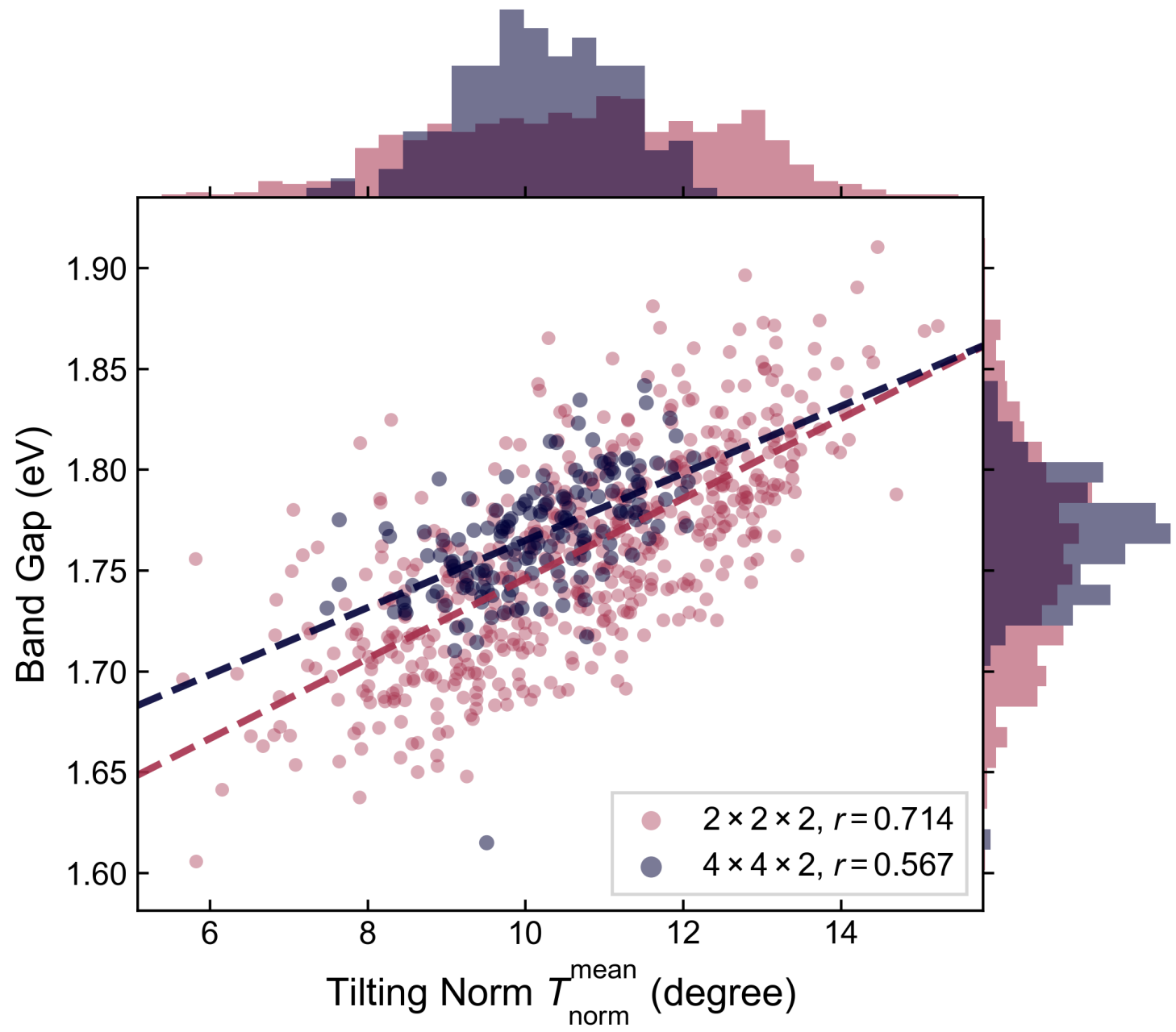


Figure S29: Sensitivity of the local structure–electronic relationship to subcell size. The same parent MD configuration is partitioned into $2 \times 2 \times 2$ and $4 \times 4 \times 2$ subcells. The marginal histograms show the distributions of the octahedron-averaged tilt norm and local band gap, while the central panel shows their correlation. Both subdivisions retain a positive tilt–gap relationship, with Pearson correlation coefficients of 0.714 and 0.567 for the $2 \times 2 \times 2$ and $4 \times 4 \times 2$ constructions, respectively. The larger subcells produce narrower distributions because the structural and electronic environments are averaged over a larger volume. The persistence of the correlation supports the use of the tilt metric as a local electronic descriptor, whereas the change in distribution width demonstrates the dependence of the absolute broadening on the spatial coarse-graining scale.

from the subcell size dependence of the absolute band gap distribution. The $2 \times 2 \times 2$ construction retains stronger local contrast and gives a Pearson correlation coefficient of 0.714. Increasing the subcell size to $4 \times 4 \times 2$ narrows both the tilt and band gap distributions and reduces the correlation coefficient to 0.567, as expected from averaging over a larger structural volume. The positive correlation nevertheless remains, showing that the tilt–gap relationship is not created solely by the periodicity of the smaller subcells. At the same time, the change in the marginal widths confirms that the absolute band-edge broadening depends on the selected spatial coarse-graining scale.

Across the full non-SOC dataset, the local band gap spans approximately 1.65–1.90 eV. These absolute values are not compared quantitatively with experiment because both spin–orbit coupling and the semilocal exchange–correlation treatment substantially affect the calculated gap.[21–23] The SOC validation in Fig. S27 confirms the expected reduction of the absolute gap while showing that the relative ordering of local-gap environments is retained. The larger non-SOC dataset is therefore used to resolve relative variations across the sampled subcell population, with the SOC subset providing an explicit sensitivity test of the principal electronic trends.

This analysis is based on one representative equilibrated MD snapshot at each temperature, subdivided into several hundred $2 \times 2 \times 2$ subcells. This choice is justified by the fact that, at fixed temperature, the octahedral tilt distributions are statistically stationary across equilibrated snapshots of the same trajectory. Although the instantaneous tilt of any given octahedron fluctuates in time, the ensemble distribution of local tilts is unchanged within statistical uncertainty from one representative frame to another. Because the electronic analysis is performed over the full set of subcells extracted from a large supercell, the resulting distributions of local band gaps and band-edge energies are therefore expected to be robust with respect to the particular frame selected. In this sense, the dominant source of variation is the equilibrium structural distribution at a given temperature rather than the choice of one specific equilibrated snapshot.

The resulting electronic bands were unfolded onto the primitive Brillouin zone to enable direct comparison across the distinct local environments generated by the MD trajectories. Each extracted $2 \times 2 \times 2$ subcell contains eight $PbI_6$ octahedra and therefore 24 local tilt components, so a reduced structural representation is required to relate the electronic variation to the local geometry in a physically interpretable way. We therefore tested multiple structural featurisations based on both octahedral tilts and distortions, and compared their correlations with the computed local band gap. As

discussed below, the strongest correlation is obtained for the octahedron-averaged tilt norm, defined by first taking the norm of the three tilt components for each octahedron and then averaging this scalar quantity over all octahedra in the subcell.

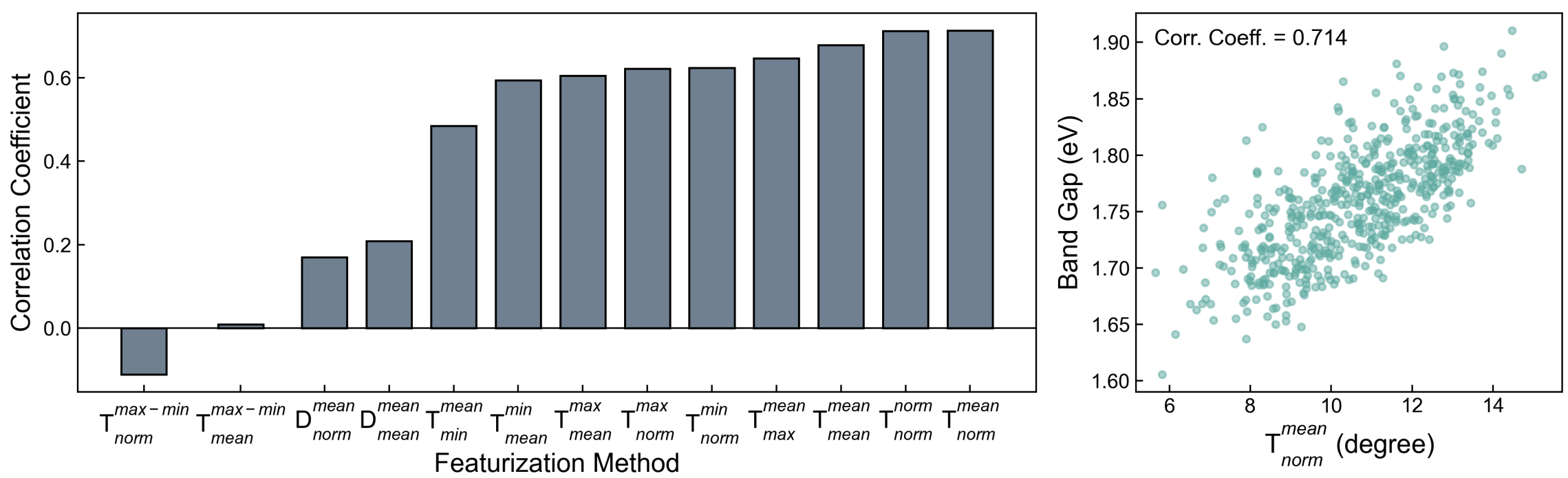


Figure S30: Correlation coefficients obtained for different structural featurisation strategies that map the local $2 \times 2 \times 2$ subcell geometry to the DFT band gap. In the notation $X_{\mathrm{sub}}^{\mathrm{sup}}$, $X$ denotes the underlying quantity ($T$: octahedral tilt, $D$: octahedral distortion), the subscript denotes the reduction at the single-octahedron level, and the superscript the reduction across all octahedra in the subcell. The strongest correlation is obtained for the octahedron-averaged tilt norm $T_{\mathrm{norm}}^{\mathrm{mean}}$, whose corresponding band gap distribution is shown in the right panel.

The comparison of candidate structural descriptors is summarised in Fig. S30. To relate the local band gap to the local structure, several reduced structural descriptors were tested. Each $2 \times 2 \times 2$ subcell contains eight octahedra, each described either by tilt components or by octahedral distortion measures. In the notation used in Fig. S30, the capital letter specifies the underlying quantity ($T$ for tilt-based features, $D$ for distortion-based features), the subscript denotes the reduction applied at the level of a single octahedron, and the superscript denotes the reduction applied across all octahedra in the subcell. For example, $T_{\mathrm{norm}}^{\mathrm{mean}}$ means first computing the norm of the three tilt components for each octahedron and then averaging this scalar quantity over all octahedra in the subcell. Among the tested featurisation strategies, the octahedron-averaged tilt norm $T_{\mathrm{norm}}^{\mathrm{mean}}$ gave the strongest correlation with the computed local band gap and was therefore adopted in the main text.

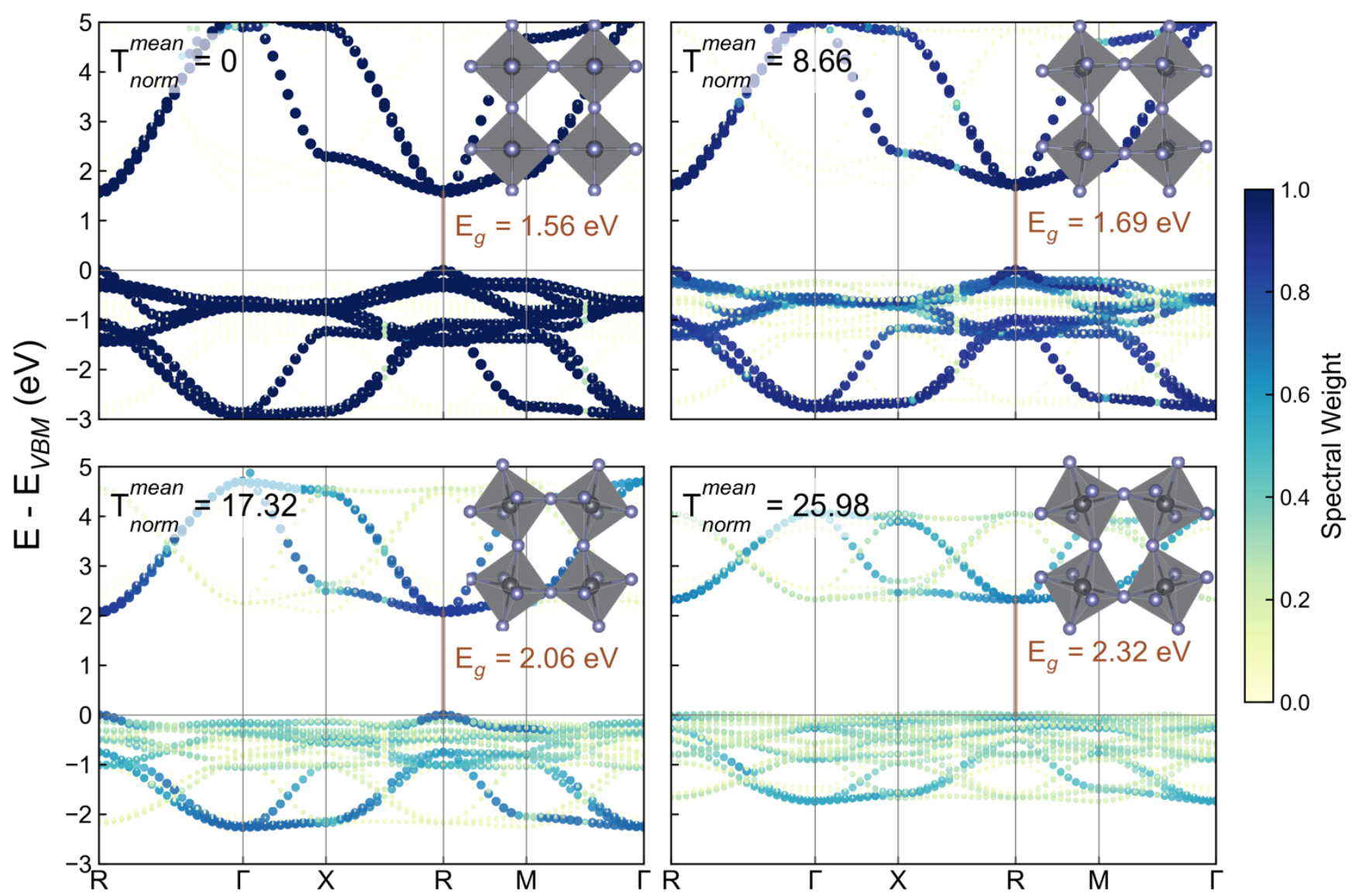


Figure S31: Idealised inorganic $a^+a^+a^+$ $FAPbI_3$ band structures used to define the reference band gap–tilt relation. The four structures shown have identical Pb−I bond lengths (3.25 Å) and single-axis tilt angles of 0, 5, 10, and 15 degrees, respectively.

To further define the structural baseline used for the band gap–tilt relation, we constructed an idealised $a^+a^+a^+$ series in which the tilt angle was varied systematically from zero to finite physical values. For this reference model, the A-site cations were removed so that the electronic structure is determined solely by the inorganic $PbI_3$ framework. This does not qualitatively alter the band gap trend of interest here, because the band-edge states in lead halide perovskites are derived predominantly from Pb and I orbitals rather than from direct A-site contributions. Within this idealised framework, the band structure is therefore governed only by the octahedral tilt amplitude and the Pb–I bond length. Fig. S31 shows four representative structures with the same bond length and $a^+a^+a^+$ tilt angles of 0, 5, 10, and 15 degrees, corresponding to $T_{\mathrm{norm}}^{\mathrm{mean}}$ values of 0, 8.66, 17.32, and 25.98 degrees. The band gaps obtained from this series define one of the ideal reference lines plotted in Fig. 5c.

# References


[1] Jinnouchi, R.; Lahnsteiner, J.; Karsai, F.; Kresse, G.; Bokdam, M. Phase Transitions of Hybrid Perovskites Simulated by Machine-learning Force Fields Trained on the Fly with Bayesian Inference. *Physical Review Letters* **2019**, *122*, 225701.

[2] Biswas, M.; Desai, R.; Bidna, G.; Mannodi-Kanakkithodi, A. Unified graph-based interatomic potential for perovskite structure optimization. *Journal of Chemical Information and Modeling* **2026**, *66*, 1353–1370.

[3] Fransson, E.; Wiktor, J.; Erhart, P. Phase transitions in inorganic halide perovskites from machine-learned potentials. *The Journal of Physical Chemistry C* **2023**, *127*, 13773–13781.

[4] Bučko, T.; Hafner, J.; Ángyán, J. G. Geometry optimization of periodic systems using internal coordinates. *The Journal of Chemical Physics* **2005**, *122*, 124508.

[5] Francis, G.; Payne, M. Finite Basis Set Corrections to Total Energy Pseudopotential Calculations. *Journal of Physics: Condensed Matter* **1990**, *2*, 4395.

[6] Liang, X.; Klarbring, J.; Baldwin, W. J.; Li, Z.; Csányi, G.; Walsh, A. Structural Dynamics Descriptors for Metal Halide Perovskites. *The Journal of Physical Chemistry C* **2023**, *127*, 19141–19151.

[7] Fransson, E.; Rahm, J. M.; Wiktor, J.; Erhart, P. Revealing the free energy landscape of halide perovskites: metastability and transition characters in CsPbBr3 and MAPbI3. *Chemistry of Materials* **2023**, *35*, 8229–8238.

[8] Bechtel, J. S.; Van der Ven, A. Octahedral tilting instabilities in inorganic halide perovskites. *Physical Review Materials* **2018**, *2*, 025401.

[9] Kingsbury, R.; Gupta, A. S.; Bartel, C. J.; Munro, J. M.; Dwaraknath, S.; Horton, M.; Persson, K. A. Performance comparison of r 2 SCAN and SCAN metaGGA density functionals for solid materials via an automated, high-throughput computational workflow. *Physical Review Materials* **2022**, *6*, 013801.

[10] Liang, X.; Klarbring, J.; Walsh, A. Phase stability and transformations in lead mixed halide perovskites from machine learning force fields. *Chemistry of Materials* **2025**, *37*, 9392–9405.

[11] Deng, B.; Choi, Y.; Zhong, P.; Riebesell, J.; Anand, S.; Li, Z.; Jun, K.; Persson, K. A.; Ceder, G. Systematic Softening in Universal Machine Learning Interatomic Potentials. *npj Computational Materials* **2025**, *11*, 9.

[12] Chmiela, S.; Sauceda, H. E.; Müller, K.-R.; Tkatchenko, A. Towards exact molecular dynamics simulations with machine-learned force fields. *Nature communications* **2018**, *9*, 3887.

[13] Musaelian, A.; Batzner, S.; Johansson, A.; Sun, L.; Owen, C. J.; Kornbluth, M.; Kozinsky, B. Learning Local Equivariant Representations for Large-scale Atomistic Dynamics. *Nature Communications* **2023**, *14*, 579.

[14] Jinnouchi, R.; Miwa, K.; Karsai, F.; Kresse, G.; Asahi, R. On-the-fly Active Learning of Interatomic Potentials for Large-scale Atomistic Simulations. *The Journal of Physical Chemistry Letters* **2020**, *11*, 6946–6955.

[15] Fabini, D. H.; Siaw, T. A.; Stoumpos, C. C.; Laurita, G.; Olds, D.; Page, K.; Hu, J. G.; Kanatzidis, M. G.; Han, S.; Seshadri, R. Universal Dynamics of Molecular Reorientation in Hybrid Lead Iodide Perovskites. *Journal of the American Chemical Society* **2017**, *139*, 16875–16884.

[16] Laven, R.; Koza, M. M.; Malavasi, L.; Perrichon, A.; Appel, M.; Karlsson, M. Rotational dynamics of organic cations in formamidinium lead iodide perovskites. *The Journal of Physical Chemistry Letters* **2023**, *14*, 2784–2791.

[17] Chen, T.; Foley, B. J.; Park, C.; Brown, C. M.; Harriger, L. W.; Lee, J.; Ruff, J.; Yoon, M.; Choi, J. J.; Lee, S.-H. Entropy-driven structural transition and kinetic trapping in formamidinium lead iodide perovskite. *Science advances* **2016**, *2*, e1601650.

[18] Weadock, N. J. et al. The Nature of Dynamic Local Order in $CH_3NH_3PbI_3$ and $CH_3NH_3PbBr_3$. *Joule* **2023**, *7*, 1051–1066.

[19] Dubajic, M. et al. Dynamic Nanodomains Dictate Macroscopic Properties in Lead Halide Perovskites. *Nature Nanotechnology* **2025**, *20*, 755–763.

[20] Wang, S.; Xiao, W.-b.; Wang, F. Structural, electronic, and optical properties of cubic formamidinium lead iodide perovskite: a first-principles investigation. *RSC advances* **2020**, *10*, 32364–32369.

[21] De Wolf, S.; Holovsky, J.; Moon, S.-J.; Loper, P.; Niesen, B.; Ledinsky, M.; Haug, F.-J.; Yum, J.-H.; Ballif, C. Organometallic halide perovskites: sharp optical absorption edge and its relation to photovoltaic performance. *The journal of physical chemistry letters* **2014**, *5*, 1035–1039.

[22] Min, H.; Kim, M.; Lee, S.-U.; Kim, H.; Kim, G.; Choi, K.; Lee, J. H.; Seok, S. I. Efficient, stable solar cells by using inherent bandgap of $\alpha$-phase formamidinium lead iodide. *Science* **2019**, *366*, 749–753.

[23] Jeon, N. J.; Noh, J. H.; Yang, W. S.; Kim, Y. C.; Ryu, S.; Seo, J.; Seok, S. I. Compositional Engineering of Perovskite Materials for High-performance Solar Cells. *Nature* **2015**, *517*, 476–480.
